# Zebrafish gonad mutant models reveal neuroendocrine mechanisms of brain sexual dimorphism and male mating behaviors of different brain regions

**DOI:** 10.1186/s13293-023-00534-7

**Published:** 2023-08-21

**Authors:** Xiangyan Dai, Ajay Pradhan, Jiao Liu, Ruolan Liu, Gang Zhai, Linyan Zhou, Jiyan Dai, Feng Shao, Zhiyong Yuan, Zhijian Wang, Zhan Yin

**Affiliations:** 1 Key Laboratory of Freshwater Fish Reproduction and Development (Ministry of Education), Key Laboratory of Aquatic Science of Chongqing, School of Life Sciences, Southwest University, Chongqing, 400715 China; 2 Biology, The Life Science Center, School of Science and Technology, Örebrorebro University, 70182 Örebro, Sweden; 3 State Key Laboratory of Freshwater Ecology and Biotechnology, Institute of Hydrobiology, Chinese Academy of Sciences, Wuhan, 430072 China

**Keywords:** Mating behaviors, Sex characteristics, Hormones, Brain dimorphism, Brain transcriptomes, Zebrafish

## Abstract

**Background:**

Sexually dimorphic mating behaviors differ between sexes and involve gonadal hormones and possibly sexually dimorphic gene expression in the brain. However, the associations among the brain, gonad, and sexual behavior in teleosts are still unclear. Here, we utilized germ cells-free *tdrd12* knockout (KO) zebrafish, and steroid synthesis enzyme *cyp17a1-*deficient zebrafish to investigate the differences and interplays in the brain–gonad–behavior axis, and the molecular control of brain dimorphism and male mating behaviors.

**Methods:**

*Tdrd12*^+/−^; *cyp17a1*^+/−^ double heterozygous parents were crossed to obtain *tdrd12*^*−/−*^; *cyp17a1*^+*/*+^ (*tdrd12 KO*), *tdrd12*^+*/*+^; *cyp17a1*^*−/−*^ (*cyp17a1 KO*), and *tdrd12*^*−/−*^; *cyp17a1*^*−/−*^ (double KO) homozygous progenies. Comparative analysis of mating behaviors were evaluated using Viewpoint zebrafish tracking software and sexual traits were thoroughly characterized based on anatomical and histological experiments in these KOs and wild types. The steroid hormone levels (testosterone, 11-ketotestosterone and 17β-estradiol) in the brains, gonads, and serum were measured using ELISA kits. To achieve a higher resolution view of the differences in region-specific expression patterns of the brain, the brains of these KOs, and control male and female fish were dissected into three regions: the forebrain, midbrain, and hindbrain for transcriptomic analysis.

**Results:**

Qualitative analysis of mating behaviors demonstrated that *tdrd12*^*−/−*^ fish behaved in the same manner as wild-type males to trigger oviposition behavior, while *cyp17a1*^*−/−*^ and double knockout (KO) fish did not exhibit these behaviors. Based on the observation of sex characteristics, mating behaviors and hormone levels in these mutants, we found that the maintenance of secondary sex characteristics and male mating behavior did not depend on the presence of germ cells; rather, they depended mainly on the 11-ketotestosterone and testosterone levels secreted into the brain–gonad regulatory axis. RNA-seq analysis of different brain regions revealed that the brain transcript profile of *tdrd12*^*−/−*^ fish was similar to that of wild-type males, especially in the forebrain and midbrain. However, the brain transcript profiles of *cyp17a1*^*−/−*^ and double KO fish were distinct from those of wild-type males and were partially biased towards the expression pattern of the female brain. Our results revealed important candidate genes and signaling pathways, such as synaptic signaling/neurotransmission, MAPK signaling, and steroid hormone pathways, that shape brain dimorphism and modulate male mating behavior in zebrafish.

**Conclusions:**

Our results provide comprehensive analyses and new insights regarding the endogenous interactions in the brain–gonad–behavior axis. Moreover, this study revealed the crucial candidate genes and neural signaling pathways of different brain regions that are involved in modulating brain dimorphism and male mating behavior in zebrafish, which would significantly light up the understanding the neuroendocrine and molecular mechanisms modulating brain dimorphism and male mating behavior in zebrafish and other teleost fish.

**Graphical Abstract:**

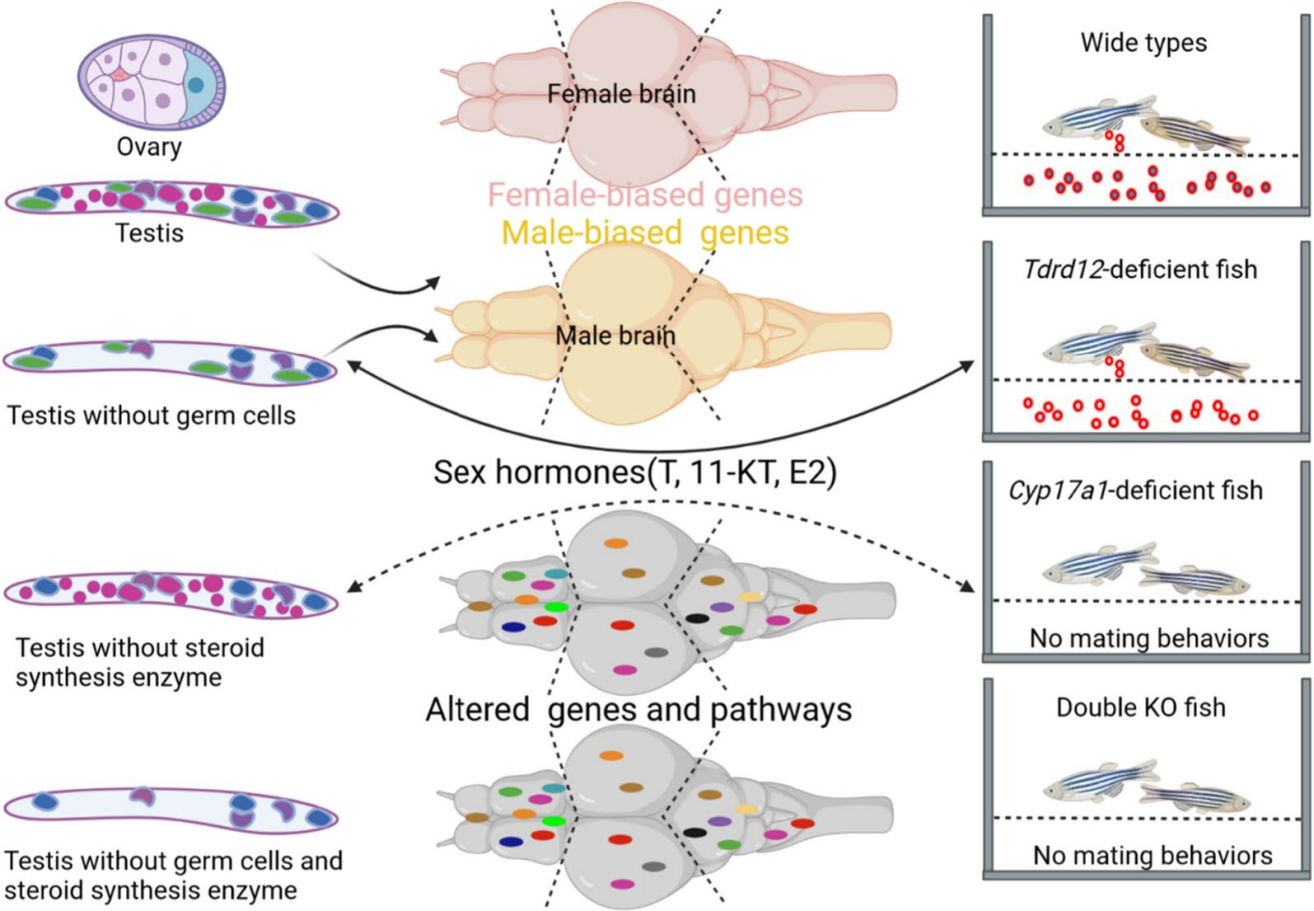

**Supplementary Information:**

The online version contains supplementary material available at 10.1186/s13293-023-00534-7.

## Highlights

1. Germ cells disruption does not change androgen levels and male mating behavior.
2. The maintenance of SSCs and male mating behavior are depended mainly on the concentrations of 11-KT and T secreted into the brain–gonad regulatory axis.
3. Germ cells loss does not have severe effects on brain functions, but disruption of homeostasis in gonadal somatic cells can have a more significant impact. Moreover, a proper interaction between germ cells and gonadal somatic cells is not only crucial for gonads but also for gene regulation in the brain.
4. This study revealed the crucial candidate genes and neural signaling pathways of different brain regions that are involved in modulating brain dimorphism and male mating behavior in zebrafish.

**Supplementary Information:**

The online version contains supplementary material available at 10.1186/s13293-023-00534-7.

## Background

All sexually reproducing animals display sex differences in behavior to ensure attraction between partners for mating. Sexual behavior constitutes a chain of mostly sex-specific behavioral acts performed by sexually mature males and females, including actions for gamete release and fusion towards producing offspring [1]. Dominant males usually display elaborate courtships to attract females for mating, and females of reproductive age evaluate the male behavior to decide whether to mate.

Secondary sexual characteristics (SSCs) are crucial for sexual selection processes and increased reproductive success during the reproduction of many animal species. In “The Origin of Species”, Darwin (1859, 1971) recognized that these traits were unlikely to contribute to survival but somehow increased reproductive success. The Descent of Man [2] provided an explanation of the evolution of SSCs as characteristics affecting success in reproductive competition or breeding. SSCs are important features that have evolved in many species (including fish species) as a consequence of inter-individual competition for mates. SSCs are features that appear at sexual maturity and consist mainly of phenotypic differences between males and females, such as body shape, body color, and specialized accessory structures. In the reproductive system, the progressive and sequential development of the reproductive tract and SSCs occurs in response to gonadal hormones; however, the neuroendocrine molecules and pathways underlying the sex differences in these traits and behaviors remain largely unknown.

The development of SSCs and sexual behavior are tightly related to gonadal hormones. The gonad is composed of germ cells and somatic cells. Leydig cells, a type of gonadal somatic cells, are the predominant cells involved in androgen production. Cytochrome P450, family 11, subfamily C member 1 (Cyp11c1) and Cytochrome P450 family 17 subfamily A member 1 (Cyp17a1) are typically expressed in these Leydig cells, Cyp11c1 and Cyp17a1 KO fish possessed mature gametes but showed low androgens in the serum, compromised mating behaviors and SSCs [3, 4]. Although the role of germ cells in sexual differentiation has been widely studied among species, there is little evidence on whether the complete loss of germ cells could affect the formation of SSCs and sexual behaviors. In most animals, gonadal sex is determined by signals from the somatic cells, such as mice [5]. Unlike in most mammals, zebrafish gonadal sex differentiation is mainly under the control of multiple genes and the number of germ cells [6–8]. Although the complete loss of germ cells lead to all-male phenotype along with sterility; interestingly, we and our collaborators found that those sterile males could behave like wild-type males to induce the female oviposition [9, 10]. Therefore, how gonadal germ cells and somatic cells affect the formation of SSCs and male-typical mating behaviors warrant deep investigation.

The activation and organization of brain sexual differentiation and sex-typical mating behaviors are highly dependent on the sex steroid hormone milieu [11, 12]. The high and steady level of circulating testosterone (T) of adult males typically activate the masculinized neural substrate to actuate male-typical phenotypes. T can both activate and organize neuronal pathways through androgen receptor (AR) activation, or it can be aromatized to 17β-estradiol (E2) by steroid biosynthesis enzymes in the brain to amplify male-typical behavior [13]. Male mice develop testicles and male SSCs when lacking AR; however, they exhibit diminished male-typical behavior [13, 14]. Steroids can modulate the transcription of a multitude of genes in the brain and ultimately influence numerous aspects of reproductive behaviors; however, the downstream targets of sex hormone signaling remain to be identified in teleost brain.

In recent years, the zebrafish has rapidly become an attractive model for studying behavioral disorders [15]. We previously produced all-male lines, Tudor domain-related protein 12 (Tdrd12) and Cyp17a1 KO fish. PIWI-interacting proteins (TDRDs) have been demonstrated to be involved in spermatogenesis and the PIWI-interacting RNA (piRNA) pathway. *Tdrd12* is specifically expressed in the gonadal germ cells, and *tdrd12* KO fish had no gonad germ cells but with gonadal somatic cells, and behaved like wild-type males capable of inducing female oviposition [9]. Though *cyp17a1* is expressed in a variety of tissues, including the brain, gill, intestine, and liver, it is predominantly expressed in the gonads of zebrafish, notably in males [16, 17]. Cyp17a1 has been acknowledged as an important enzyme in steroid hormone synthesis that catalyzes the oxidations of progesterone and pregnenolone and is the major source of androgens. *cyp17a1* KO zebrafish were all male, showed normal germ cell development but compromised male mating behaviors and lower plasma androgen levels [3]. Our mutant lines are excellent models to unravel the contribution of gonadal germ cells and gonadal somatic cells in male SSCs and mating behaviors through brain–gonad axis. Importantly, our results could provide new insights into how internal regulators of hormones from the gonad to produce brain sexual differences in mating behaviors.

## Materials and methods

### Animals and ethics

The culture of zebrafish and all experimental procedures were conducted according to the zebrafish handbook [18] and with the regulations of the Care and Use of Laboratory Animals under the approval by the Ethics Committee of Laboratory Animal Experimentation of Southwest University (IACUC-20180303-01).

Zebrafish embryos were obtained by natural spawning from laboratory domesticated lines and were cultured in egg water. Zebrafish larvae and adults were maintained with dechlorinated tap water in a circulating system at 26 ± 1 °C under a 14 h light and 10 h dark photoperiod light on time. Fish were fed 3 times per day with live brine shrimp and commercial pellet food (Petna, Xiamen, china). Sexually mature, spawning adults (3–6 months) were randomly assigned to experimental groups and used for the following analyses. Analysis was usually performed in parallel; hence, the same animal was used for behavioral experiments, blood or tissues sample collection or gene expression. All sampling was conducted under 1–4 h after onset of the light period in the morning.

### Production of mutant lines

*Tdrd12*^+/−^; *cyp17a1*^+/−^ double heterozygous parents were crossed to obtain *tdrd12*^+*/*+^; *cyp17a1*^+*/*+^ (Con males and females)*, tdrd12*^*−/−*^; *cyp17a1*^+*/*+^ (*tdrd12*^*−/−*^), *tdrd12*^+*/*+^; *cyp17a1*^*−/−*^ (*cyp17a1*^*−/−*^), *tdrd12*^*−/−*^; *cyp17a1*^*−/−*^ (double KO) homozygous progenies. The progenies were raised to adulthood for genotyping and further analysis.

For genotyping, DNA was isolated using a previously described method [19] with minor modification. Briefly, a small caudal fin was clipped from juveniles or adult fish, then lyzed with ~ 50 μL 10 mM NaOH for 20–40 min with intermittent vortexing for proper lysis at 95 °C and then 2 μL DNA was directly used for PCR. The primers used in this assay were in Additional file 1: Table S2.

### Behavioral analysis

The courtship behaviors of zebrafish during mating usually consist of the male chasing or swimming closely alongside, around, or in front of the female in a tight circle or figure of eight with his fins raised; these behaviors trigger oviposition of 5 to 20 eggs at a time by the female, and the male simultaneously releases sperm. These sequential behaviors are repeated throughout the mating/spawning period [20, 21].

In this assay, females were paired with wild-type males before the mating experiments to test whether the females engaged in normal mating behavior and oviposition. After spawning successfully for 3 times, the female zebrafish were randomly mated with the wild-type male, *tdrd12*^*−/−*^, *cyp17a1*^*−/−*^, double KO fish for mating behavioral analysis. Then, 12 individuals were measured for each genotype, and each experiment was repeated for 3 times. All behavioral procedures were conducted in 2-l rectangular spawning chamber with dechlorinated tap water.

On the day before behavioral analysis, each female was randomly mated with one male in the dark, separated by a transparent partition. The following morning, the spawning chambers were placed under light period for 1 h, and then the partition was removed to allow the fish to interact for reproduction for 30 min. In this assay, intimate contact distance or chasing distance was defined as less than 1.5 cm between a male and a female during the courtship, and this distance was used to define both the frequency and the duration of intimate contact. The full mating process lasted for 30 min, of which the first 8 min were recorded using Viewpoint zebrafish tracking software (ViewPoint Life Sciences, Zebra001, Lyon, France) for analysis. Egg releasing behaviors and fertility were observed and calculated. To allow recovery time for males and females, a period of 1 week was set as a buffer time for consecutive experiments.

### Sex ratios

The sex ratios of homozygous progenies from the in-cross of *tdrd12*^*+/−*^ and *cyp17a1*^*+/−*^ heterozygous mutant fish were calculated at 4 mpf (month post fertilization). The gonads were observed under the microscope and histological analysis was performed to determine the sex.

### Analysis of sexual traits

In this assay, about 10 fish of each genotype were sacrificed for identification of sexual traits. The SSCs were identified based on the coloration on the fins, body shape, presence or absence of anal papilla and presence or absence of BTs (epidermal tubercles) in pectoral fins, respectively. In general, females are characterized by a bluish-white coloration on the body or anal fin and possess a protruding egg-filled abdomen, with the presence of anal papilla, whereas males have a pinkish-gold body or anal fin coloration, a more streamlined body profile with the presence of BTs in pectoral fins, respectively. Anatomical examinations were conducted after the fish were anaesthetized in 0.10–0.20 mg/ml tricaine methanesulfonate (Aldrich, St Louis, MO, USA).

### Anatomy and histology

For the preparation of tissue samples, the adults were first euthanized with tricaine. Tissue samples were dissected and snap frozen in liquid nitrogen and then stored at − 80 °C until further use. For staining, the samples were fixed in 4% PFA (paraformaldehyde) for at least 2 days, washed in PBS, and then gradient dehydration in alcohol, paraffin embedding, sectioning at 6 μm. H&E (hematoxylin and eosin) staining was performed according to the standard procedures.

### Detection of hormone levels

Individuals of each genotype (6–10) were grouped for sampling for the detection of steroid hormone levels in the gonads and brains. The tissue samples were homogenized in ELISA dilution buffer and kept on ice with intermittent vortexing for 15 min. The samples were centrifuged at 13,000 rpm for 15 min at 4 °C, and then the supernatants were transferred to new tubes. Blood from 3 individual fish was pooled to obtain one serum sample [9]. The steroid hormone levels (testosterone (T), 11-ketotestosterone (11-KT), 17β-estradiol (E2)) were measured using ELISA kits (Cayman Chemical Company, 582701/582281/582751, Michigan, USA) according to the manufacturer’s instructions. For T and 11-KT assay, the detection range were from 3.9 to 500 pg/ml and 0.78 to 100 pg/ml, while for E2, it was a range from 0.9 to 2000 pg/ml. The absorbance was measured using spectrophotometry.

### RNA-seq analysis

In this assay, 9 fish were divided into 3 groups for each genotype and samples were performed in biological triplicates and each sample consisted 3 fish. The brain was dissected into three regions: (1) forebrain contains olfactory bulb and telencephalon; (2) midbrain contains tectum, hypothalamus; (3) hindbrain contains cerebellum and medulla. The tissue samples were snap frozen in liquid nitrogen and stored at − 80 °C until further use. RNA isolation and purification were conducted following the standard protocols according to the RNA extraction kit (Promega, Shanghai, China). Total RNA of various brain regions were sequenced using Illumina2000 Hi-Seq technology at OmicShare (Guangzhuo, China). The remaining RNAs were used for cDNAs synthesis using the Reverse Aid First-Strand cDNA Synthesis Kit (Thermo Scientific, Waltman, MA, USA).

The differentially expressed genes (DEGs) were subsequently used for functional gene ontology (GO) term enrichment and kyoto encyclopedia of genes and genomes (KEGG) pathway analysis at metascape (http://metascape.org/).

### Real time quantitative PCR

Quantitative real-time PCR (RT-qPCR) primers were designed using primer blast from the National Center for Biotechnology Information (NCBI) database. Primers used in qPCR assay are summarized in Additional file 1: Table S2. qPCR was performed using TB Green Premix Ex Taq (Takara, Ohtsu, Japan). Each sample was performed in biological triplicates. The relative mRNA level was calculated by the normalized CT values (2^−ΔΔCt^). *gapdh*, *β-actin2*, *eef1a1* were run for all the samples and *β-actin2* was selected as the most suitable and stable reference gene. To facilitate comparisons in qPCR analysis, the expression level of each target gene (normalized to *β-actin2*) was arbitrarily set to 1, and the relative difference was calculated.

### Statistical analysis

Statistical analyses were performed using GraphPad Prism (San Diego, CA). Briefly, continuous variables were compared between two groups by the unpaired two-tailed Student’s *t* test; Welch’s correction to the Student’s *t* test was employed when the variance differed significantly between groups by the *F* test. For more than two comparison groups, continuous variables were compared by one-way or two-way analysis of variance (ANOVA), followed by either Bonferroni’s post hoc test for comparisons among groups or Dunnett’s post hoc test for comparisons of experimental groups versus control groups. *P < 0.05, significant difference; **P < 0.01, significant difference; ***P < 0.001.

## Results

### Comparative analysis of sexual behaviors and sexual traits among single and compound t*drd12;cyp17a1* KO

The single *tdrd12* and *cyp17a1* KO fish showed male phenotypes (Additional file 1: Fig. S1) but somehow different male mating behaviors [3, 9]. To dissect the cellular basis responsible for the differential sexual behaviors in these two mutants, we generated *tdrd12;* *cyp17a1* double KOs and further did behavioral tracking analyses to resolve their mating behaviors better, and sexual traits of these KOs were thoroughly characterized.

**Fig. 1:**
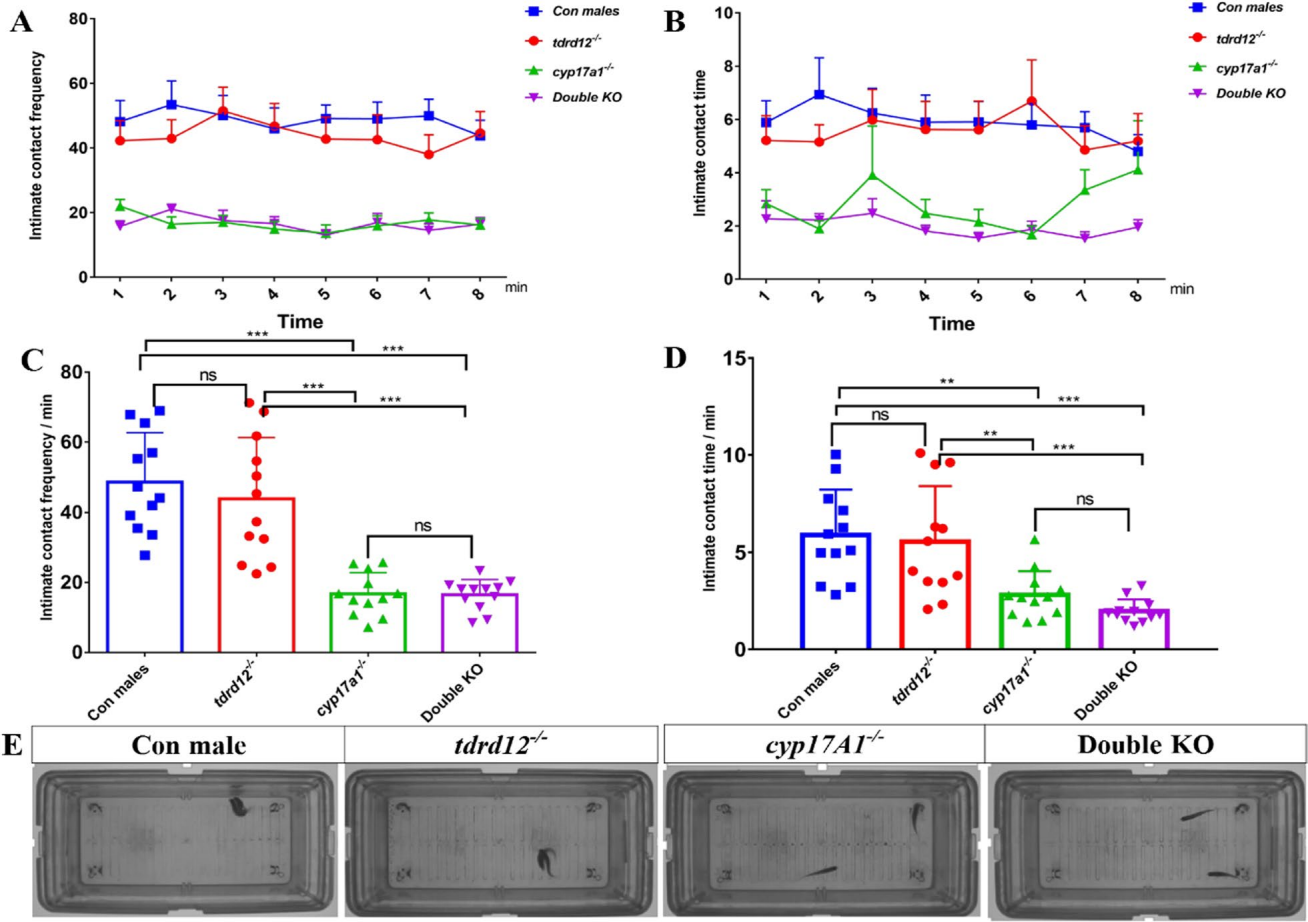
Courtship parameters of wild-type male, *tdrd12*^*−/−*^, *cyp17a1*.^*−/−*^ and double knockout (KO) fish with wild-type females. Twelve individuals of each genotype were selected and randomly paired with wild-type females, and all pairs were exposed to light for 20 min before the mating partition was removed. **A** Frequency of intimate contact with wild-type females during the courtship period. **B** Duration of intimate contact time with females during the courtship period. **C** Average frequency of intimate contact with females per minute during the courtship period. **D** Average duration of intimate contact time with females per minute during the courtship period. **E** Representative mating behaviors of males of four genotypes with wild-type females. *Significant difference (*P* < 0.05); **significant difference (*P* < 0.01); ***significant difference* P* < 0.001

First, 12 individuals of each genotype were selected and randomly paired with wild-type females, the parameters such as the frequency and duration of intimate contact with wildtype females were recorded during the courtship, and the oviposition and fertilization rates were recorded after mating. As shown in Fig. 1, The results showed that in *tdrd12*^*−/−*^ mutants, similar male mating behavior parameters could be observed as in wild-type males, they engaged in intense courtship behaviors towards females (chasing the female or swimming closely beside, around or in front of her in a tight circle or figure of eight, etc.), resulting in a high frequency (44 frequency/min) and duration of intimate contact time with the females (Fig. 1). *tdrd12*^*−/−*^ fish successfully induced the female to spawn during the process of courtship. Although there was no difference in the number of eggs released by the female when mated with wild-type or *tdrd12*^*−/−*^ males; however, the eggs were all unfertilized when the females were mated with *tdrd12*^*−/−*^ males (Additional file 1: Table S1). In contrast, in *cyp17a1*^*−/−*^ and double KO fish, the courtship parameters were both significantly lower (2–3 times) than that of wild-type males and *tdrd12* KO fish. These compromised male mating behaviors in *cyp17a1*^*−/−*^ and double KO fish resulted in a failure to court and induce the females to spawn (Fig. 1 and Additional file 1: Table S1). These results demonstrated that *tdrd12*^*−/−*^ mutants behaved in the same manner as wild-type males to induce female oviposition behavior, while *cyp17a1*^*−/−*^ and double KO fish did not. These differences in male mating behavior imply that there is a strong relationship between mating behaviors and gonads.

**Fig. 2:**
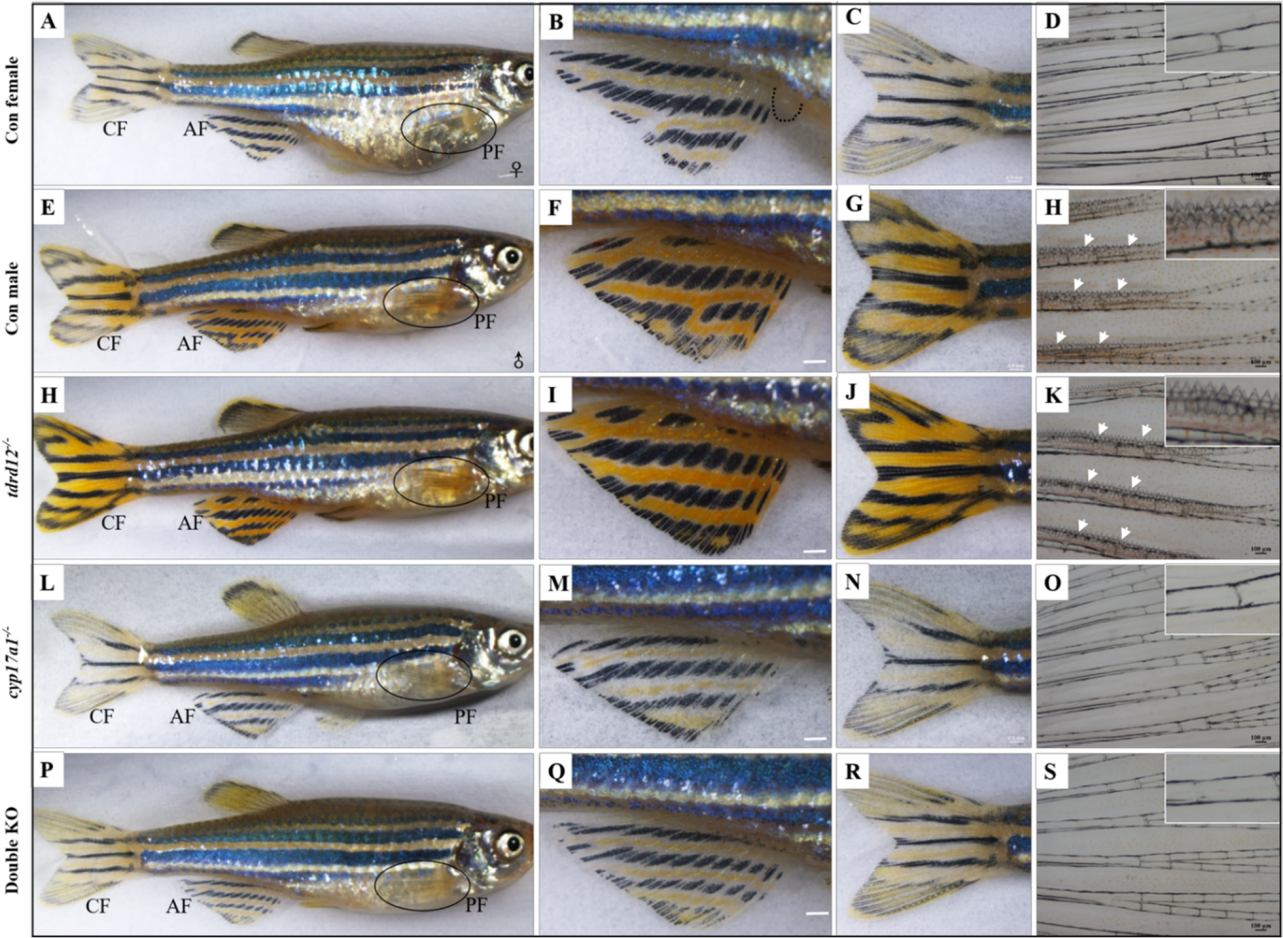
Identification of secondary sex characteristics (SSCs). **A**–**D** SSCs of wild-type females: body shape/body color (**A**), anal fin and anal papilla (**B**), caudal fin (**C**) and pectoral fin (**D**) without breeding tubercles. **E**–**H** SSCs of wild-type males: body shape/color (**E**), anal fin (**F**), caudal fin (**G**) and pectoral fin (**H**). The arrow indicates a large number of breeding tubercles, which occur specifically in males. **H**–**K** SSCs of *cyp17a1*^+*/*+^*; tdrd12*^*−/−*^: body shape/body color (H), anal fin (**I**), caudal fin (**J**) and pectoral fin (**K**) with breeding tubercles. **L**–**O** SSCs of *cyp17a1*^*−/−*^: body shape/body color (**L**), anal fin (**M**), caudal fin (**N**) and pectoral fin (**O**) without breeding tubercles. **P**–**S** SSCs of double KO mutants: body shape/body color (**P**), anal fin (**Q**), caudal fin (**R**) and pectoral fin (S) without breeding tubercles. mpf: months post-fertilization; PF: pectoral fin; AF: anal fin; CF: caudal fin. 6 individuals of each genotype were detected in this assay

Then, SSCs of 6 individuals of each genotype were thoroughly observed. Absolutely different from wild-type females (Fig. 2A–D), the *tdrd12*^*−/−*^ fish exhibited similar SSCs as wild-type males; with typically pinkish-gold color and a notable streamlined body and large numbers of breeding tubercles (BTs) specifically on the pectoral fins (Fig. 2E–K). *cyp17a1*^*−/−*^ and double KO fish, despite having a streamlined body shape without anal papilla, were more inclined with wild-type females with bluish-white coloration on the body or fins. Most importantly, no BTs could be observed on their pectoral fins (Fig. 2L–S). These results suggest that the signals from the gonadal somatic cells are critical for the maintenance of SSCs in males, not the germ cells.

**Fig. 3:**
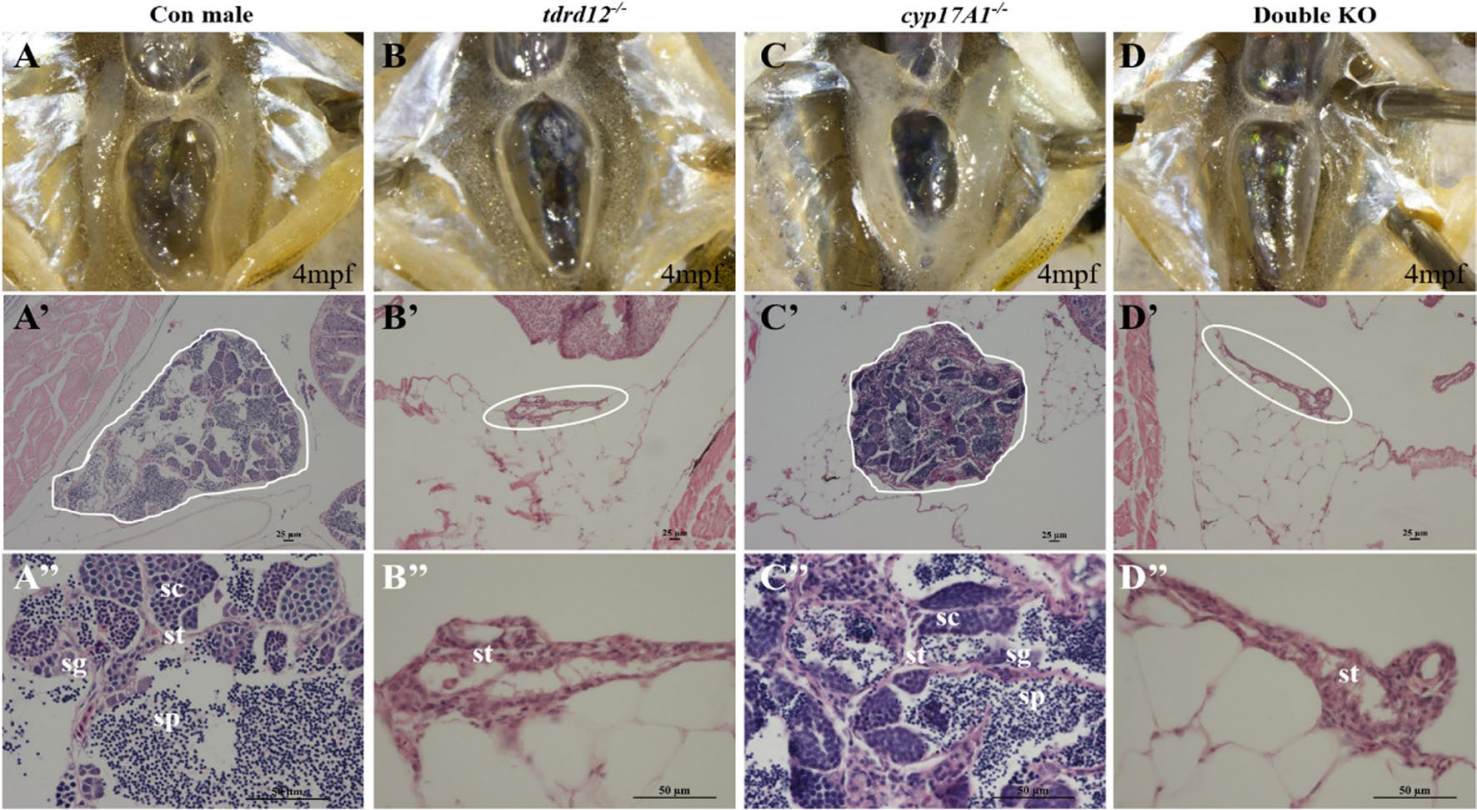
Morphology of primary sex characteristics (PSCs) of the mutants. **A**–**A**″) Normal testis of a control male. Anatomical view (A); histological view of the whole testis (**A**′, testis outlined in white); enlarged view shows a well-developed testis with various developmental stages of germ cells, such as spermatocytes (SC), spermatogonia (SG), sperm (SP) and somatic cells (ST) in the testis (**A**″). **B**–**B**″ Atrophic testis of a *tdrd12*^*−/−*^ fish; the testis lacks germ cells and is surrounded by fatty layers. Anatomical view (**B**), histological view (**B**′, outlined in white) and enlarged view (**B**″). **C**–**C**″ Testis of a *cyp17a1*^*−/−*^ fish. Anatomical view; **C** histological view (**C**′, testis outlined in white); enlarged view shows the testis with various developmental stages of germ cells, such as SC, SG, sperm and some ST (**C**″). **D**–**D**″ Atrophic testis of a double knockout (KO) fish. Anatomical view (**D**); histological view (**D**′, testis outlined in white); enlarged view (**D**″) shows an atrophic testis similar to that of the *tdrd12*^*−/−*^ fish. 6 fish of each genotype were used in this assay

Further analysis of the gonads showed that *tdrd12*^*−/−*^ fish exhibited atrophic testes surrounded by fatty layers (Fig. 3B–B″). Haematoxylin and eosin (HE) staining showed that the testes lacked sperm-producing cells but had some visible gonad somatic cells. In contrast, *cyp17a1*^*−/−*^ fish had the complete testicular structure as wild-type males; spermatids, spermatocytes, spermatogonia, and somatic cells could be observed (Fig. 3A–A″, C–C″). The testes of double KO mutants were similar to those of *tdrd12*^*−/−*^ fish (Fig. 3D–D″). Together, these results demonstrate that the development of male SSCs and normal male mating behaviors are mainly dependent on the signals from gonadal steroid producing cells, not on germ cells.

### A deficit of the sex hormone correlates with abnormal male-typical mating behaviors through the brain–gonad regulatory axis

To investigate the role of sex hormones on SSCs and sexual behaviors in teleosts, T, E2 and 11-KT levels were determined in the gonad, brain and serum of these KOs and wild types.

**Fig. 4:**
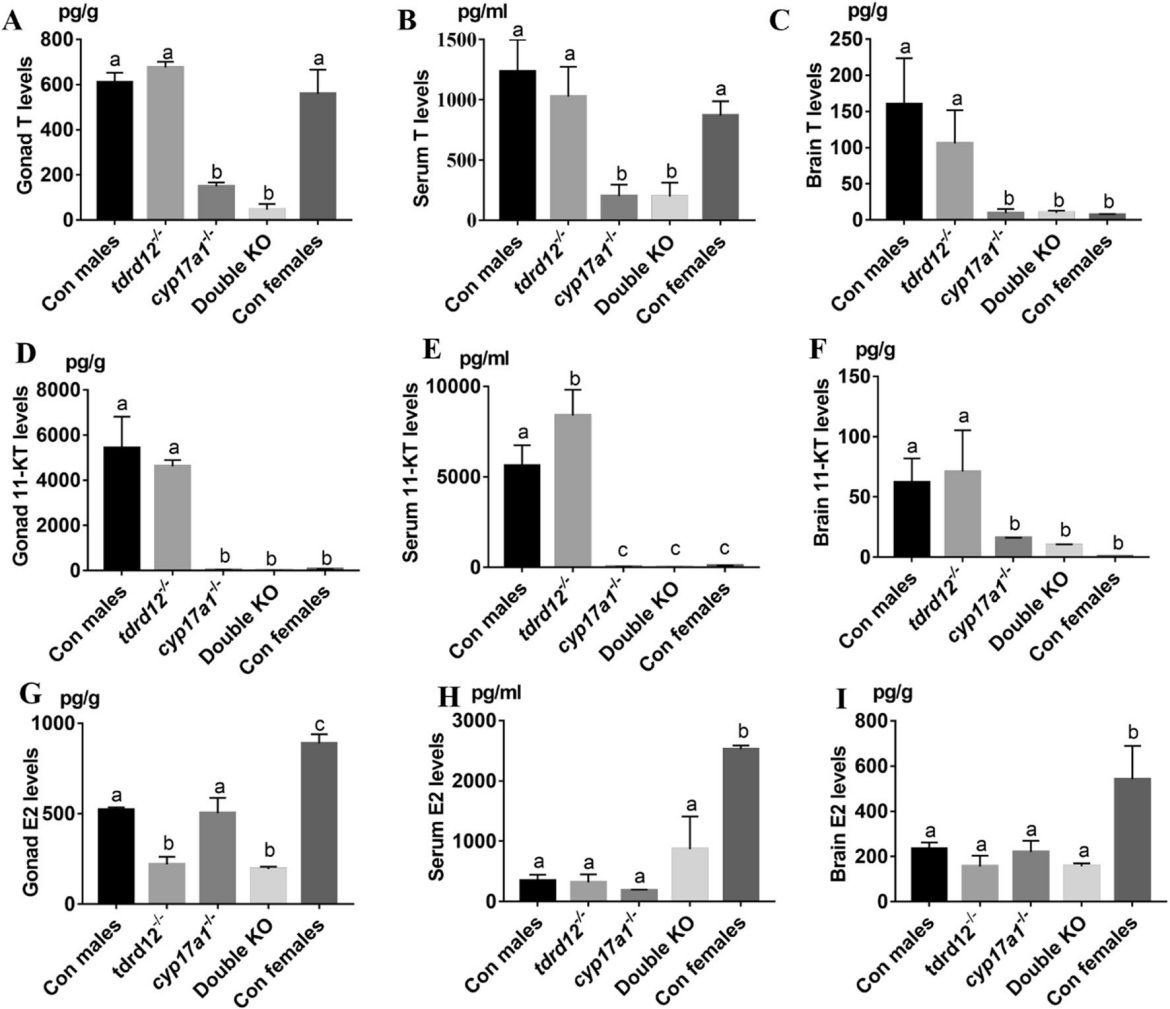
Hormone concentrations of testosterone (T), 11-ketotestosterone (11-KT) and 17β-estradiol (E2) in the gonads, serum, and brain of control males, *tdrd12*^*−/−*^ fish, *cyp17a1*.^*−/−*^ fish, double knockout (KO) fish and control females. **A**–**C** T levels in the gonads, blood, and brain samples. **D**–**F** 11-KT levels in the gonads, blood, and brain samples. **C** E2 levels in the gonads, blood, and brain samples. For this assay, the numbers of gonadal, blood, and brain samples were *n* = 4, *n* = 6 and *n* = 8; Error bars represent the mean ± SEM. Bars with different letters are significantly different from each other, *P* < 0.05

Wild-type males and females showed significantly different T, E2 and 11-KT levels in the brain–gonad axis. Wild-type females had similar T levels as that in the wild-type males in the gonad and serum (Fig. 4A, B), but had significantly lower levels of T in the brain (Fig. 4C). 11-KT levels in the brain, serum and gonad of wild-type females were all significantly lower than in that of males (Fig. 4D–F). The E2 levels in the brain, serum and gonad of wild-type females were all significantly higher than in that of males (Fig. 4G–I).

In the gonad and serum, the concentration of T in *cyp17a1*^*−/−*^ and double KO fish was significantly lower than that in *tdrd12*^*−/−*^*,* wild-type males and wild-type females (Fig. 4A, B). In the brain, the T level in *tdrd12*^*−/−*^ remained the same as that in the wild-type males but was significantly higher than that in *cyp17a1*^*−/−*^ fish, double KO fish and wild-type females (Fig. 4C). The 11-KT level in *tdrd12*^*−/−*^ fish was similar to that of wild-type male siblings in gonads and brains but was higher than wild-type males in the serum. In addition, both *tdrd12*^*−/−*^ fish and wild-type males, the 11-KT level was significantly higher than *cyp17a1*^*−/−*^, double KO fish and wild-type females (Fig. 4D–F). The levels of E2 in the serum, gonads and brain of these mutants and wild-type males were significantly lower than wild-type females, and no significant difference was found among the other three mutants and wild-type males (Fig. 4G–I). E2 levels in wild-type males and the mutants was lower than wild-type females in all the three analyzed samples (Fig. 4G–I). Interestingly, gonadal E2 levels in *tdrd12*^*−/−*^ and double KO fish was lower than wild-type and *cyp17a1*^*−/−*^ males (Fig. 4G).

Previously, we showed that the administration of T or 11-KT could restore the phenotype of SSCs and male mating behavior defects in *cyp17a1* KO fish [22]. Together with the behavioral results and sexual traits in these KOs, we conclude that the maintenance of SSCs and male mating behavior of zebrafish do not depend on the presence of germ cells in the gonadal tissue; rather, they rely mainly on the presence of a certain level of 11-KT and T in the brain–gonad regulatory axis.

### Significant transcriptome change in different brain regions of mutant fish

**Fig. 5:**
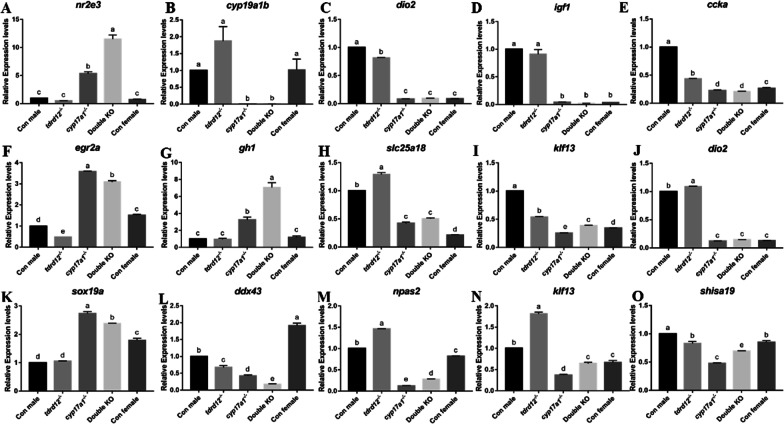
RT‒qPCR was performed to validate the RNA transcriptome data in different brain region samples. **A**–**D** *nr2e3, dio2, cyp19a1b,* and *igf1* in the forebrain; **E**–**H** *egr2b, gh1, slc25a18, klf3,* and *dio2* in the midbrain; **I**–**L** *sox19a, ddx43, npas2, klf3,* and *shisa19* in the hindbrain. Error bars represent the mean ± SD; bars with different letters are significantly different from each other,* P* < 0.05. Brain samples were *n* = 5 in biological triplicates

To achieve a higher resolution view of the differences in region-specific expression patterns of the brain, the brains of *tdrd12*^*−/−*^, *cyp17a1*^*−/−*^, double KO, and control male and female fish were dissected into three regions: the forebrain, midbrain, and hindbrain. Transcriptome analysis was performed for each region and the differentially expressed genes (DEGs) were filtered under the following parameters: |log_2_ fold change|> 1; *P* value < 0.05. The number of DEGs were summarized in Additional file 1: Fig. S3, the qPCR verification of 5 genes for each brain region are shown in Fig. 5 and all these transcripts exhibited a similar expression pattern to that observed in the RNA-seq data (Additional file 1: Fig. S4).

First, we found that the transcript profile of wild-type males and females showed distinct differences, with an overall higher number of altered genes in the forebrain, midbrain, and hindbrain (Additional file 1: Figs. S3 and 6). These female-upregulated genes (female-biased) and male-upregulated (male-biased) genes were screened as the sex-biased candidate genes constantly involved in the brain differentiation.

**Fig. 6:**
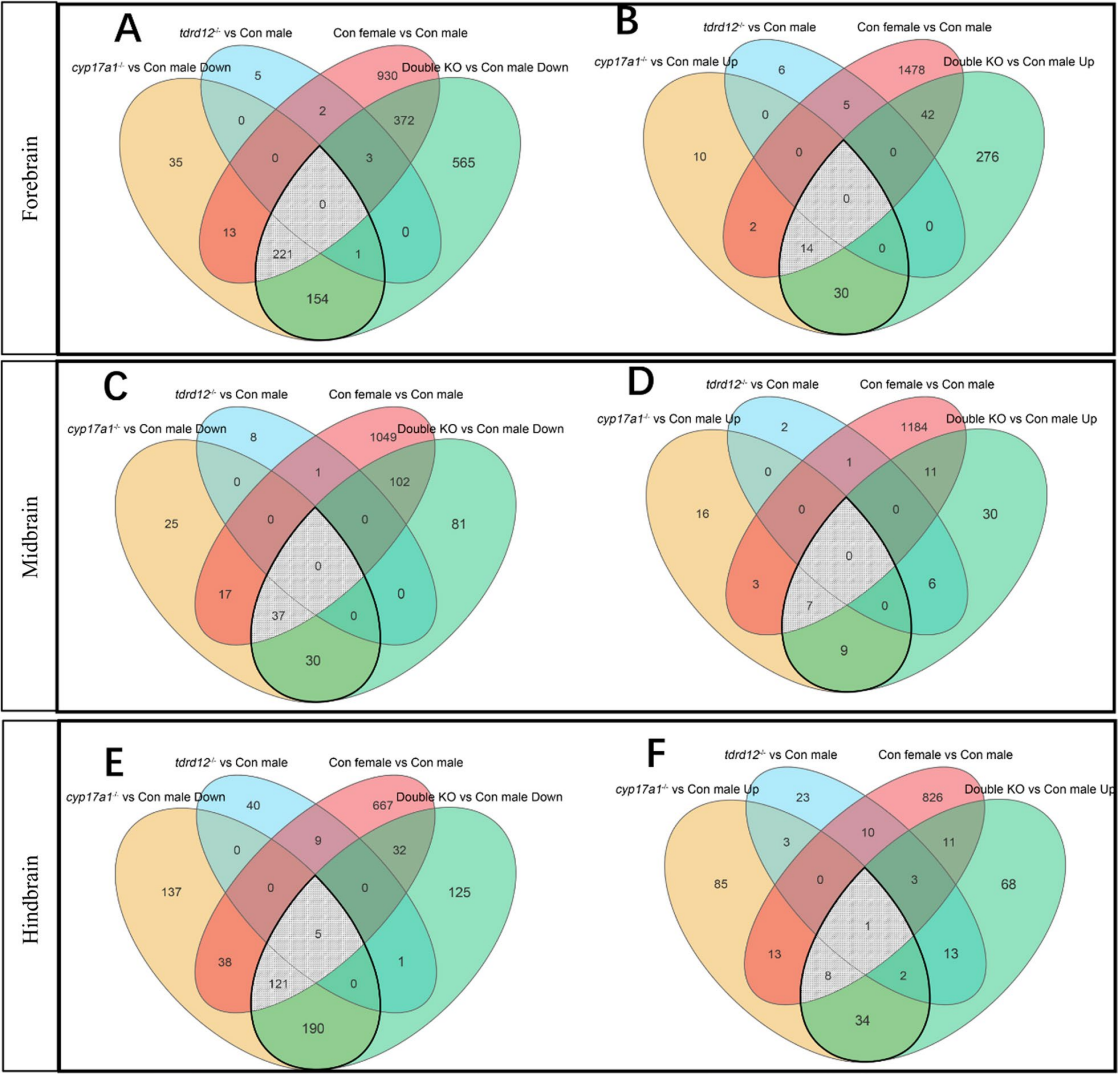
Venn diagram for significantly differentially expressed genes (DEGs) in *tdrd12*^*−/−*^ fish, *cyp17a1*^*−/−*^ fish, double knockout (KO), and wild-type female fish compared with wild-type males in the forebrain (**A**, **B**), midbrain (**C**, **D**), and hindbrain (**E**, **F**) (*P* < 0.05, |fold change|> 2). The drop-shaped regions with thick black outlines shows those shared genes are shown in in *cyp17a1*^*−/−*^ and double KO fish compared with wild-type male brains; grey gridline areas shows those co-DEGs shared by *cyp17a1*^*−/−*^, double KO fish, and females compared with wild-type male brains

We then determined the transcriptomic differences in *tdrd12*^*−/−*^, *cyp17a1*^*−/−*^ and double KO fish brains with wild types in different brain regions (Fig. 6). The transcriptomic results showed very few genes were altered in *tdrd12*^*−/−*^ fish when compared to wild-type males, especially in the forebrain and midbrain. In contrast, in *cyp17a1*^*−/−*^ fish, there were 424, 109, and 474 downregulated genes and 56, 35, 140 upregulated genes in forebrains, midbrains, and hindbrains; in double KO fish, there were 1316, 250, 491 downregulated genes and 362, 57, 140 upregulated genes in forebrains, midbrains, and hindbrains compared to wild-type males. Notably, *cyp17a1*^*−/−*^ and double KO fish shared many altered genes when compared with wild-type males: 379 co-downregulated and 44 co-upregulated genes in the forebrain, 67 co-downregulated and 16 co-upregulated genes in the midbrain, 321 co-downregulated and 45 co-upregulated genes in the hindbrain (shown by water droplets in Fig. 6). Moreover, we found that most of those DEGs in *cyp17a1*^*−/−*^ fish and double KO fish (with wild-type males) were male-biased genes, especially in double KO fish (Figs. 6 and Additional file 1: Fig. S5). These results revealed that the brain transcript profile of *tdrd12*^*−/−*^ fish was similar to that of wild-type males; however, Cyp17a1 deficiency induces a significant transcriptome change in different brain regions, especially in the forebrain and hindbrain.

### Male-specific gene signature in the brain is partially lost in mutant fish with behavior defects

**Fig. 7:**
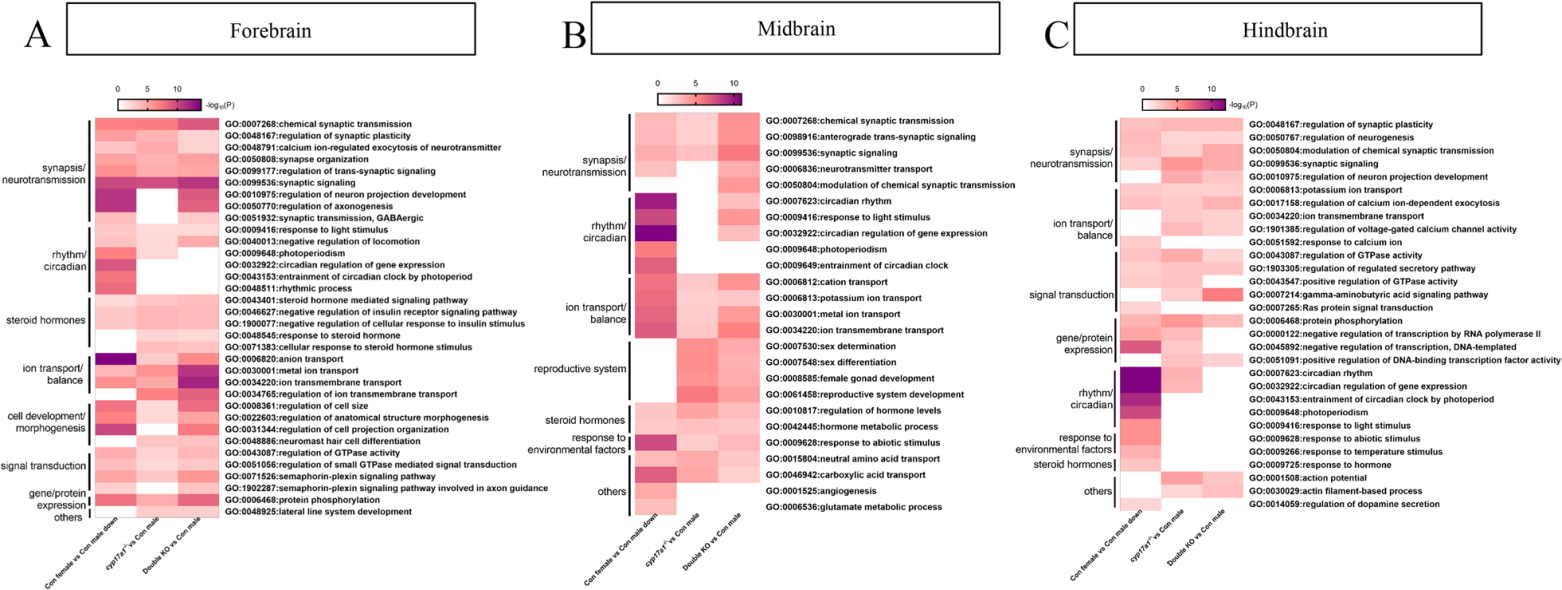
Heatmap shows the top Gene Ontology (GO) terms of male-biased genes, differentially expressed genes (DEGs) in *cyp17a1*.^*−/−*^ fish, and DEGs in double knockout (KO) fish in comparison with wild-type males in the forebrain (**A**), midbrain (**B**) and hindbrain (**C**)

To further probe the similarities and differences in male mating behavior between defective males and wild types. We performed GO analysis and found that most of the affected pathways in *cyp17a1*^*−/−*^ and double KO mutants were highly overlapped with those male-biased genes (Fig. 7 and Additional file 1: Fig. S5), such as synaptic signaling pathways and ion transport in the three brain regions, steroid hormone pathways in the forebrain and midbrain, and signal transduction pathways in the forebrain and hindbrain. We also found that there is a large difference in the regulation process of rhythmic/circadian between males and females in the zebrafish brain, and the brain transcript profiles of *cyp17a1*^*−/−*^ and double KO fish were more biased to male brain rhythms or circadian cycles (Fig. 7A–C). These transcriptomic analyses provide new insights into the discovery of genes and signaling pathways in zebrafish brain sex differentiation. Combined with the deficiency in male-typical SSCs and mating behavior in *cyp17a1*^*−/−*^ and double KO fish, the above results further suggest that the male specific gene signature in the brain is partially lost and the molecular pathways could be partially biased towards the female brain in *cyp17a1*^*−/−*^ and double KO fish.

### Analysis of DEGs uncovers putative genes and signaling pathways in the brain that may modulate male-typical mating behaviors

To identify the main candidate genes and molecular pathways involved in shaping brain neural networks of male-typical mating behaviors mediated by T or 11-KT, we analyzed the co-DEGs (shown by water droplets in Fig. 6) in *cyp17a1*^*−/−*^ and double KO fish against wild-type males.

**Fig. 8:**
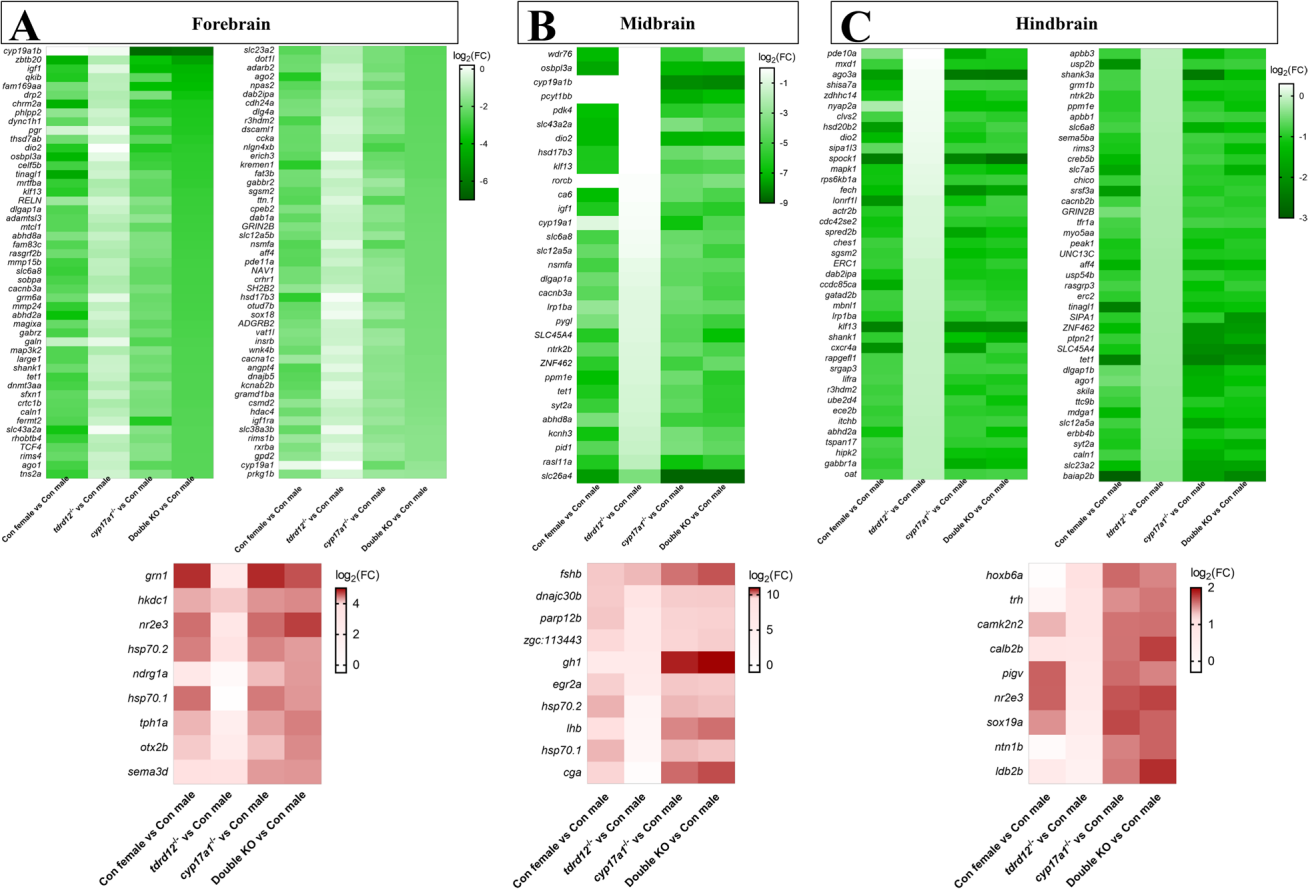
Heat map shows the representative co-downregulated (upper) and co-upregulated (down) gene expression in wild-type females, *tdrd12*^*−/−*^ fish, *cyp17a1*.^*−/−*^ fish or double KO fish when compared with wild-type males in the forebrain (**A**), midbrain (**B**) and hindbrain (**C**)

In the forebrain, 376 downregulated and 44 upregulated genes were analyzed, the most affected genes, such as brain aromatase (*cyp19a1b*), zinc finger and BTB domain containing 20 (*zbtb20)*, progesterone receptor (*pgr*), iodothyronine deiodinase 2 (*dio2)*, synaptotagmin IIa (*syt2a*), galanin (*galn*), insulin-like growth factor 1 (*igf1*) (Fig. 8A and Additional file 1: Table S3). The affected genes were enriched in MAPK signaling, GnRH, GABAergic synapse, Calcium signaling, Oxytocin signaling, Progesterone-mediated and Estrogen signaling pathways shown in Additional file 1: Fig. S6A, B.

In the midbrain, only 67 downregulated and 16 upregulated genes were analyzed, respectively; for example, WD repeat domain 76 (*wdr76*), *cyp19a1b*, carbonic anhydrase VI (*ca6*), *dio2*, pyruvate dehydrogenase kinase (*pdk4*), *igf1* (Fig. 8B and Additional file 1: Table S4). The affected genes were mainly distributed in MAPK, GnRH signaling, Sex differentiation or determination, Steroid or hormone biosynthesis, Synaptic signaling pathways (Additional file 1: Fig. S6C, D).

In the hindbrain, we found 316 downregulated and 45 upregulated genes, such as MAX dimerization protein 1 (*mxd1*), *pgr*, *dio2*, calneuron 1(*caln1*), *igf1*, *syt2a* (Fig. 8C and Additional file 1: Table S5). These affected genes were enriched in ErbB signaling, MAPK signaling, Insulin and Estrogen signaling, FoxO signaling, Synaptic signaling, glutamatergic and GABAergic synaptic transmission, Negative regulation of transcription pathways (Additional file 1: Fig. S6E, F).

## Discussion

If and to what extent germ cells regulate sexual behaviors, SSCs formation and brain dimorphism are elusive in mammals. Our previous study and colleagues showed that sterile fish without primordial germ cells (PGCs) or germ cells could behave like wild-type males to induce female oviposition upon visual observation in zebrafish [9, 10]. Here, we further characterized the mating behaviors and SSCs of these mutant fish, and conclude that male germ cells are not required for male mating behavior, SSCs formation, as well as brain masculinization in zebrafish. Transcriptomic data indicate that *tdrd12*^*−/−*^ fish brain exhibit a mostly male gene expression pattern, which is somehow different from the incomplete brain masculinization in *dnd* morphant (MO) fish by analyzing a limited number of genes in the whole brain [10]. Although the gonadal and brain androgen levels were not different in *dnd* MO fish compared to wild-type males [10], we speculate that the early PGC loss together with the interaction between PGCs and gonadal somatic cells could impair the early steroidogenesis process or other critical process in brain–gonad axis which may cause the brain phenotype in *dnd* MO fish, while *tdrd12*^*−/−*^ fish had normal early germ cells development until 18 days post fertilization. Although similar compromised male mating behaviors, SSCs and hormone levels were observed in *cyp17a1*^*−/−*^ and double KO, more DEGs were found in the brains of double KO fish than in those of *cyp17a1*^*−/−*^ fish compared to wild-type males. Further studies are needed to determine whether the stage-dependent interaction between germ cells and gonadal somatic cells, or gonadal steroidogenesis process contributes to brain masculinization in zebrafish.

Previously [22] and in this study, we show that *cyp17a1* KO fish has disrupted steroidogenesis process and the administration of T or 11-KT could restore the phenotype of SSCs and male mating behavior defects in *cyp17a1*^*−/−*^ fish [22]. Here, the introduction of the *cyp17a1* mutation in *tdrd12*^*−/−*^ fish showed that *tdrd12*^*−/−*^ fish harbor normal steroid-producing cells in gonadal somatic cells. Therefore, the similar T and 11-KT levels in the brain–gonad axis in *tdrd12*^*−/−*^ males compared to wild-type males could lead to the observed brain masculinization and normal male mating behaviors. This indicates that the compromised male-typical mating behaviors could be due to reduced androgen levels and disruption of androgen-mediated signaling or other neural signaling pathways in the brain. Both E2 and T are needed for masculinization of the brain and are responsible for regulating sexual activity in mammals [23, 24]. To our surprise, E2 levels were unchanged in *cyp17a1*^*−/−*^ and double KO fish brains compared to wild-type males and *tdrd12*^*−/−*^ fish and were significantly lower than those in wild-type females. These differences in brain E2 levels between teleost and mammals suggest that E2 is important for masculinization of the mammalian brain, while it mostly feminizes the teleost brain.

In the subcortical social brain (SSB) in teleosts, the supracommissural nucleus of the ventral telencephalon (Vs) is homologous to the mouse basal nucleus of the stria terminalis (BNST) and medial basolateral amygdala (MeA) areas, the medial preoptic area (mPOA) is homologous to the mouse preoptic hypothalamus (POA), and the anterior tuberal nucleus (ATN) is homologous to the ventromedial hypothalamus (VMH) [15]. The brain regions of the MeA, VMH, POA and BNST have previously been implicated in the control of reproductive and territorial behaviors, and more aromatase-positive neurons and higher levels of steroid receptors could be found in males in these regions than in females, which is a necessary and critical site of steroid action for the activation of male copulatory behavior [24–27]. These social brain regions indicate that reproductive behavioral regions are mainly located in the forebrain and some in the midbrain, which is consistent with our finding that more DEGs were detected in the forebrain compared with hindbrain and midbrain. Interestingly, the cerebellum is also important for integrating sensory perception and motor control (motor and emotional responses and spatial cognition), and Purkinje and granule cells serve as the major gamma-aminobutyric acid (GABA)-ergic and glutamatergic neurons in the cerebellum, which has critical roles in male mating behaviors in mammals [28]. Together, it is of interest to determine sex differences in these brain regions and how these regions would affect sexual mating behaviors in teleosts.

Sexual behavior differs between sexes and involves possibly sexually dimorphic gene expression in the brain. Our current study extends previous report (Shu et al. [22]) and we identified additional candidate genes and regulatory pathways that were involved in brain dimorphism and mating behavior in different brain regions. In the present study, we found that MAPK signaling pathway was significantly altered in three brain regions, while FOXO and AGE/RAGE signaling pathways were mainly enriched in the hindbrain. Moreover, we further found that *igf1* and *dio2* were male-biased genes and altered in different brain regions; *igf1* was downregulated in the forebrain and midbrain, while *dio2* was downregulated in three brain regions of single and double KOs. The brain specific aromatase (*cyp19a1b*) was downregulated in both forebrain and midbrain, while *pgr* was only downregulated in the forebrain.

IGF1 is suggested to play diverse roles in brain development, testicular function, and growth by activating MAPK and phosphoinositide 3 kinase (PI3K) signaling in mammals; for example, IGF-1 primarily increases the release of gamma-aminobutyric acid (GABA) in neurons [29, 30]. Igf1 is also involved in the organization of GnRH neurons in juvenile zebrafish [31], and a previous study proposed that GnRH signaling together with Igf1 may thus be involved in zebrafish sexual behavior [32]. Dio2 is involved in thyroid hormones (THs) signaling pathway, and is the predominant deiodinase in zebrafish which can catalyze the conversion of the prohormone T4 into the transcriptionally active T3. Mutation of zebrafish *dio2* leads to defective locomotor activities and reproductive dysfunction with relatively normal gamete development [33, 34]. Thyroid exposure can lead to male biased populations of zebrafish [35] and in thyroid-stimulating hormone subunit beta a (*tshba*) mutant fish impaired development of the SSCs and lower T3 and T4 level were observed [36], suggesting TH is important for SSCs formation. Together with the finding that *dio2* was significantly downregulated in three brain regions of female, *cyp17a1*^*−/−*^ and double KO fish and was enriched in hormonal regulation and developmental pigmentation pathways, we suggest that *dio2* could also be a key player in brain dimorphism, and involved in male SSCs formation in zebrafish.

Steroids can modulate the transcription of a multitude of genes in the brain and ultimately influence reproductive behaviors. The brain's neuroendocrine networks play key roles in appropriate male-specific reproductive physiology and behavior in mammals [27, 37]. Androgens strongly affect brain cell phenotypes and modulate the expression of specific enzymes, such as aromatase; nuclear receptors, such as *ar* and *pgr*; neuropeptides or neurotransmitters, such as galanin, somatostatin, neurotensin, oxytocin and dopamine; and calbindin in various brain regions in mammals [25, 38–42]. Interestingly, our results showed the significant downregulation of the brain aromatase *cyp19a1b*; *pgr;* neuropeptide cholecystokinin (*ccka*); synaptotagmin (*syt2a*); galanin (*galn*); and transcription factors, such as myocardin-related transcription factor Ba (*mrtfba*), transcription factor 4 (*tcf4*), and CREB regulated transcription coactivator 1b (*crtc1*b); the GABAergic synapse receptors *gabrz*, *gabrb2*, which could be responsible for mating behavior-defects. This indicates that these regulated genes could be master candidate genes in regulating male-typical mating behavior. The aromatase *cyp19a1b* promoter has an estrogen response element (ERE) and an androgen response element (ARE) [23, 24]. *Pgr* could be upregulated by estrogens in the brain radial glial cells and neurons of adult zebrafish [43]. We suggest that both *cyp19a1b* and *pgr* could respond to estrogen or androgen in the teleost brain to regulate male mating behavior, which extends our previous report [22]. In mice, sexually dimorphic genes encoding intracellular signaling proteins *Sytl4* have been implicated in regulating sexual behaviors [44]. A recent study showed that Syt2a localizes to synapses which is implicated in social behavior in the ventral forebrain and co-localizes with a biosynthetic enzyme in the dopamine pathway in zebrafish [45]. This suggests *syt2a* is also an important component in male mating behaviors through synaptic signaling pathway in teleost. Galanin regulates color pattern formation in the zebrafish, and mutations in the genes coding either for galanin receptor 1A (*galr1A*) or for its ligand (*galn*) result in fewer stripes and a pale appearance [46]. Those findings, together with our results, suggest that *galn* may respond to androgens to affect male body color formation in zebrafish. Together, our detailed analysis of these mutants demonstrates that the compromised male-typical mating behaviors in *cyp17a1*^*−/−*^ and double KO fish may be due to reduced androgen levels and disruption of crucial genes and androgen-mediated signaling or other neural signaling pathways in the brain.

Aside from the crucial pathways and genes mentioned above, other candidate genes and pathways are also involved in brain function and mating behaviors. The genes distributed in complex brain neural networks may directly or indirectly respond to hormone levels to influence SSCs and mating behavior. Gene deletion or genetically targeted functional manipulation of restricted neurons could help us generate specific deficits in one or more components of sexual displays while leaving other components intact in the future.

## Summary

Sexually dimorphic mating behaviors differ between sexes and involve gonadal hormones and possibly sexually dimorphic gene expression in the brain. However, the associations among the brain, gonad, and sexual behavior in teleosts are still unclear. Here, utilizing germ cells-free *tdrd12* knockout (KO) fish, we show that germ cells disruption does not change androgen levels, male mating behavior and secondary sex characteristics (SSCs). However, in steroid synthesis enzyme *cyp17a1* KO and double KO fish, the androgen was reduced, resulting in disrupted male mating behavior and compromised male SSCs. Germ cells disruption does not appear to influence mating behavior, and the brain transcriptomic analysis of *tdrd12* KO fish indicates that it can only impact different brain processes in the hindbrain. Most importantly, in *cyp17a1* KO and double KO fish, the brain transcript profiles were distinct from those of wild-type males and were partially biased towards the expression pattern of the female brain in different brain regions. Based on this, it can be emphasized that germ cells loss does not have severe effects on brain functions, but disruption of homeostasis in gonadal somatic cells can have a more significant impact. Moreover, a proper interaction between germ cells and gonadal somatic cells is not only crucial for gonads but also for gene regulation in the brain. At this stage, it is not clear how germ cells loss impacts brain transcript profiles. Understanding this regulation could reveal new genes and pathways in gonad–brain axis control.

## Perspectives and significance

PGCs and gonadal somatic cells are known to be critical in the development of proper gonads and brain functions. The androgens secreted by the gonadal somatic cells have been identified as an important regulator of the brain–gonad axis. Our study shows that germ cells disruption does not change androgen levels and male-typical mating behavior. The maintenance of secondary sex characteristics and male mating behavior are depended mainly on the concentrations of 11-KT and T secreted into the brain–gonad regulatory axis. Germ cells loss does not have severe effects on brain functions, but disruption of homeostasis in gonadal somatic cells can have a more significant impact. Accordingly, a proper interaction between germ cells and gonadal somatic cells is not only crucial for gonads but also for gene regulation in the brain. Moreover, this study revealed the crucial candidate genes and neural signaling pathways of different brain regions that are involved in modulating brain dimorphism and male mating behavior in zebrafish, which would significantly light up the understanding the neuroendocrine and molecular mechanisms modulating brain dimorphism and male mating behavior in other vertebrate animals.

### Supplementary Information

**Additional file 1.** Supplementary figures and tables.

## Data Availability

The authors declare that all data supporting the findings of this study are available upon reasonable request. The transcriptomic data were deposited at Genome Sequence Archive (GSA, NO: PRJCA011140).

## Abbreviations

11-KT: 11-Ketotestosterone
ATN: Anterior tuberal nucleus
AR: Androgen receptor
ARE: Androgen response element
BNST: Bed nucleus of the stria terminalis
BTs: Breeding tubercles
Ccka: Cholecystokinin
Crtc1b: CREB regulated transcription coactivator 1b
Cyp17a1: Cytochrome P450 17 A1
DEGs: Differentially expressed genes
Dio2: Iodothyronine deiodinase 2
ERE: Estrogen response element
GABA: Gamma-aminobutyric acid
Galn: Galanin
Galr1A: Galanin receptor 1A
Igf1: Insulin-like growth factor 1
KO: Knockout
MPF: Month post fertilization
Pdk4: Pyruvate dehydrogenase kinase 4
PGC: Primordial germ cells
Pgr: Progesterone receptor
PI3K: Phosphoinositide 3 kinase
POA: Preoptic hypothalamus
SSB: Subcortical social brain
SSCs: Secondary sexual characteristics
Syt2a: Synaptotagmin
T: Testosterone
Tdrd12: Tudor domain-related protein 12
TH: Thyroid hormone
VMH: Ventromedial hypothalamus
Wdr76: WD repeat domain 76
WT: Wild-type
