# Supplementary material for: Zebrafish gonad mutant models reveal neuroendocrine mechanisms of brain sexual dimorphism and male mating behaviors of different brain regions
Source: Biol Sex Differ. 2023 Aug 21;14:53. doi: 10.1186/s13293-023-00534-7 (PMC10440941; doi:10.1186/s13293-023-00534-7)
Supplement: Supplementary file 1 — Additional file 1. Supplementary figures and tables. [file 13293_2023_534_MOESM1_ESM.docx]

**Zebrafish Gonad Mutant Models Reveal Neuroendocrine Mechanisms of Brain Sexual Dimorphism and Male Mating Behaviors of Different Brain Regions**


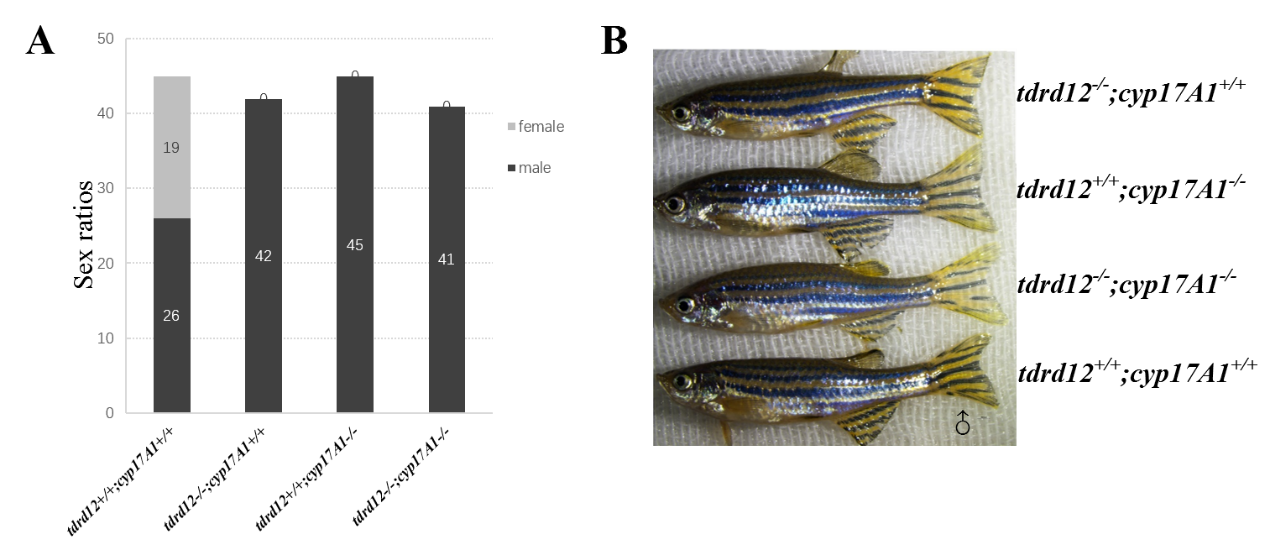


Figure S1. The male phenotypes of homozygous progenies from the in-cross of *tdrd12^+/-^* ;*cyp17a1^+/-^*. (A) The sex ratios of the homozygous progenies from the in-cross of *tdrd12^+/-^* ;*cyp17a1^+/-^* heterozygous mutant fish (90 dpf). Total number of progenies for this analysis is 674, among these progenies, all homozygous developed into males, mixed sex ratios of males and females were observed in WT. (B) The male phenotype of homozygous progenies. From the top to bottom, there were *tdrd12^-/-^;cyp17a1^+/+^* (*tdrd12^-/-^*), *tdrd12^+/+^;cyp17a1^-/-^*( *cyp17a1^-/-^*) , *tdrd12 ^-/-^;cyp17a1^-/-^* (*double KO*) and wild type male.


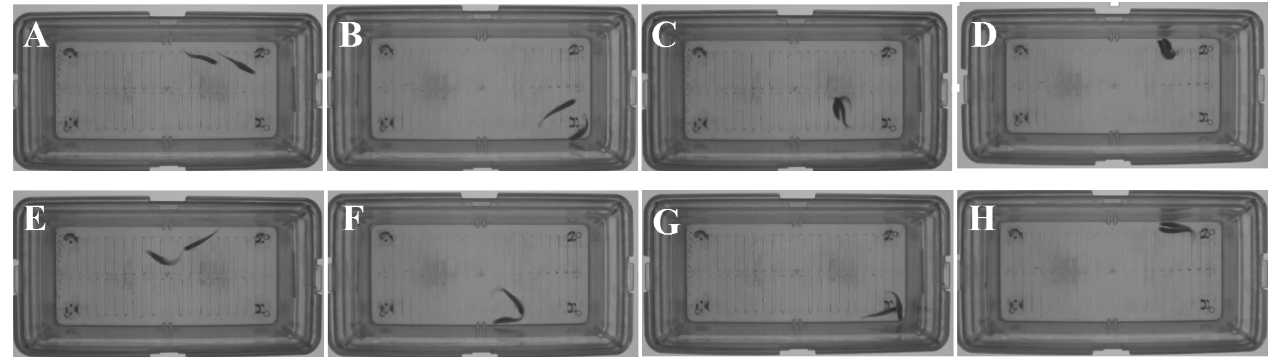


Figure S2. The representative courtship behaviors of control male individuals and *tdrd12^-/-^* individuals with wild type female individuals. (A -D) Chasing the female, swimming around or in front of her in a tight circle, collision and parallel behaviors between wide type males and wide type females; (E-F) Chasing the female, swimming around or in front of her in a tight circle, collision and parallel behaviors between *tdrd12^-/-^* fish and wide type females.


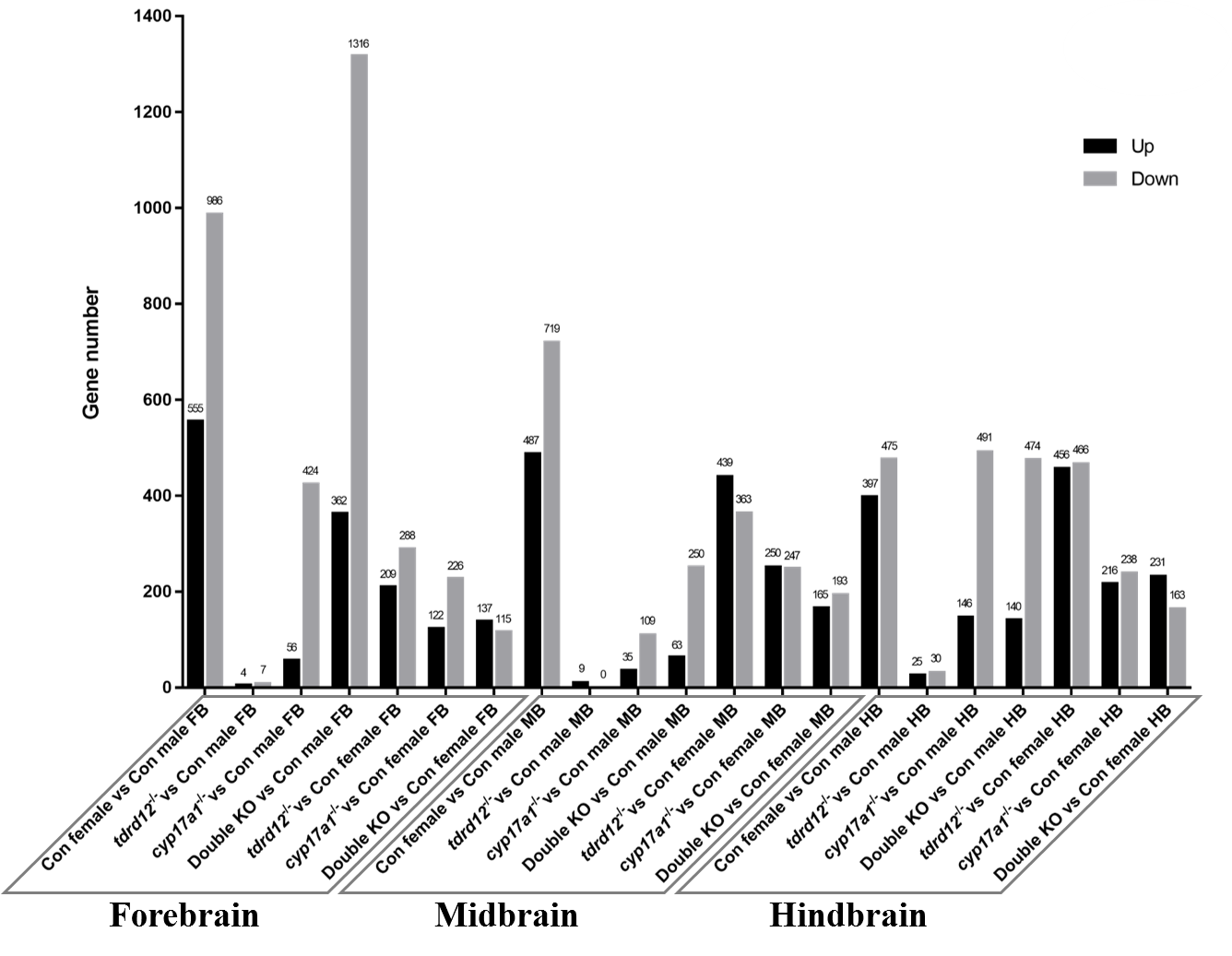


Figure S3. Statistics of significantly differentially expressed genes (DEGs) in different brain regions of *tdrd12^-/-^* fish, *cyp17a1^-/-^* fish, and double knockout (KO) fish compared with wild-type males and females at 4 months post-fertilization (*P*<0.05, |fold change|>2). FB: Forebrain; MB: Midbrain HB: Hindbrain


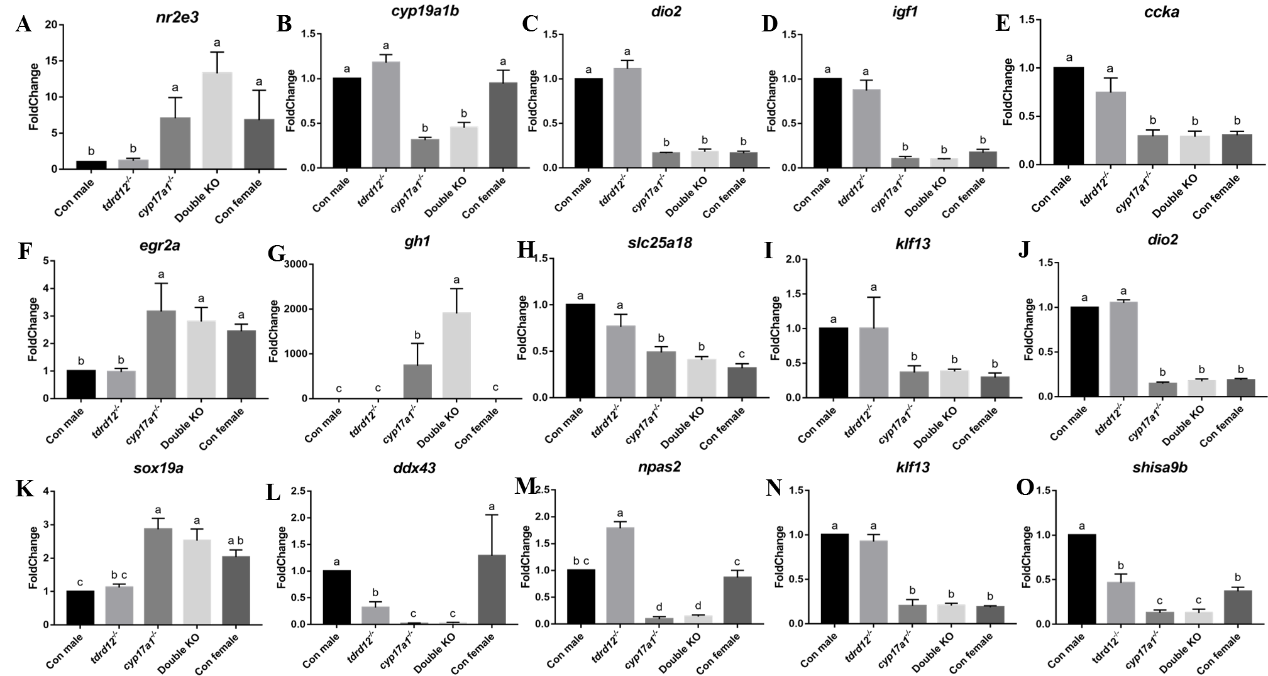


Figure S4. The fold changes (RNA-seq data) in the expression of genes of interest, as measured by RT‒qPCR. (A-D) *nr2e3, dio2, cyp19a1b,* and *igf1* in the forebrain*;* (E-H) *egr2b, gh1, slc25a18, klf3,* and *dio2* in the midbrain*;* (I-L) *sox19a, ddx43, npas2, klf3,* and *shisa19* in the hindbrain.


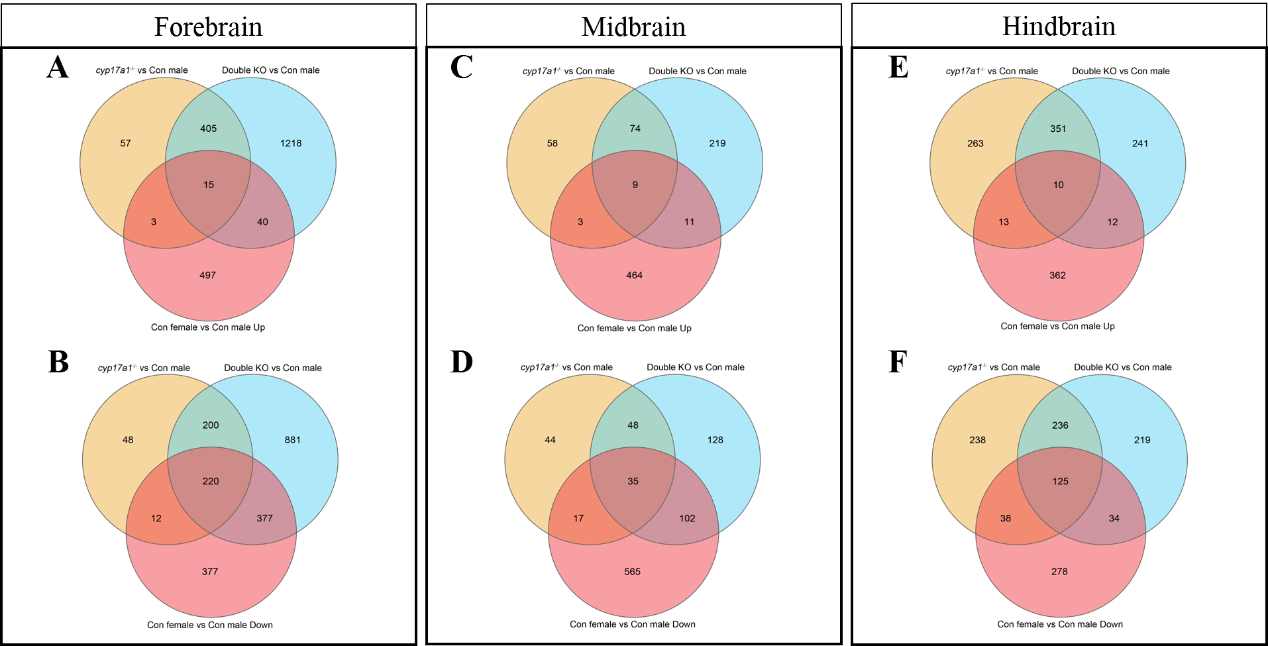


Figure S5. Venn diagram of DEGs in *cyp17a1^-/-^* fish and double KO fish when compared with wild-type males, with female-biased or male-biased genes in the forebrain (A and B), midbrain (C and D), and hindbrain (E and F).


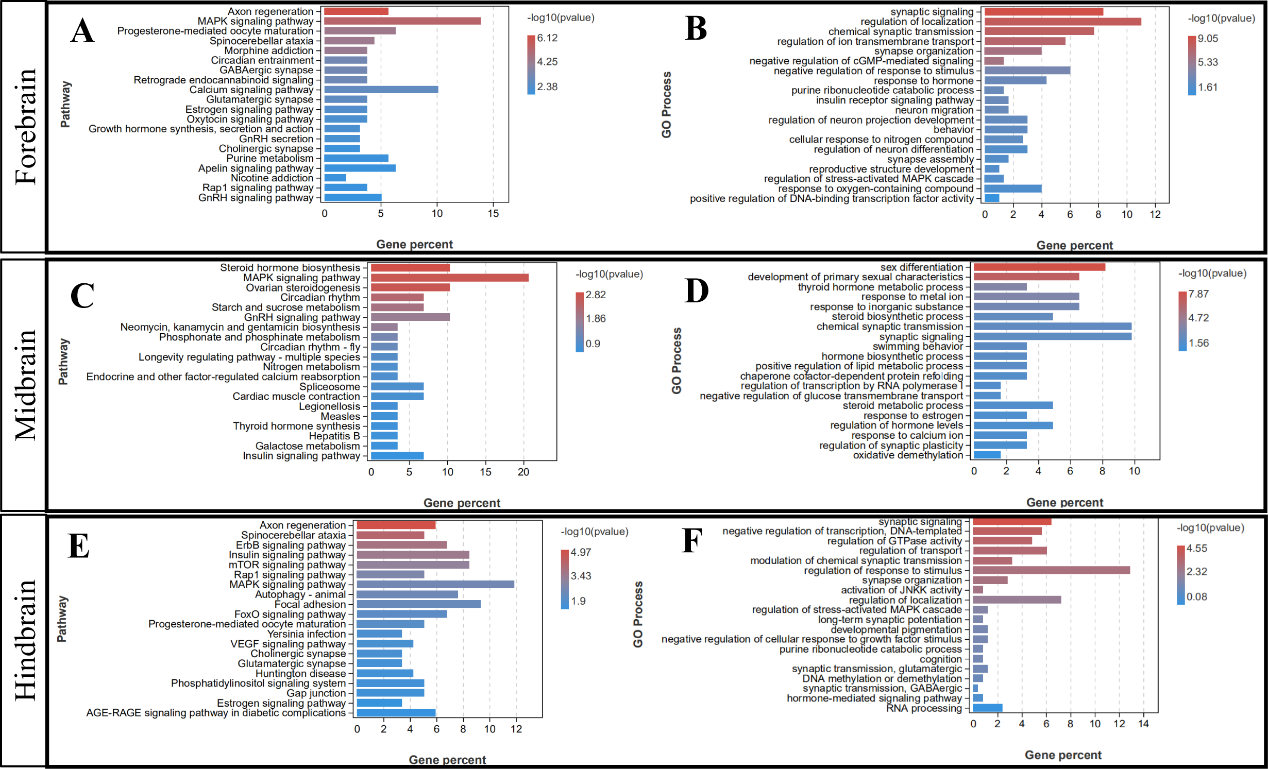


Figure S6. The molecular pathways involved in male mating behavior. Gene Ontology (GO) and Kyoto Encyclopedia of Genes and Genomes (KEGG) analyses were performed on altered genes in different brain regions of both *cyp17a1^-/-^* and double KO fish compared with those of wild-type males. The top 20 KEGG pathways altered in the forebrain (A), midbrain (C) and hindbrain (E). Representative affected GO terms for biological processes in the forebrain (B), midbrain (D) and hindbrain (F).

Table 1 Oviposition of these mating females and fertilization rates from these mating groups.

| **Con male** | | | ***tdrd12^-/-^*** | | | ***cyp17a1^-/-^*** | | | **Double KO** | | |
| --- | --- | --- | --- | --- | --- | --- | --- | --- | --- | --- | --- |
| Total eggs | Fertilized eggs | Fertilization rate | Total eggs | Fertilized eggs | Fertilization rate | Total eggs | Fertilized eggs | Fertilization rate | Total eggs | Fertilized eggs | Fertilization rate |
| 289 | 256 | 89% | 231 | 0 | 0% | 0 | 0 |  | 0 | 0 |  |
| 219 | 199 | 91% | 258 | 0 | 0% | 0 | 0 |  | 0 | 0 |  |
| 213 | 195 | 92% | 198 | 0 | 0% | 0 | 0 |  | 0 | 0 |  |
| 193 | 181 | 94% | 263 | 0 | 0% | 0 | 0 |  | 0 | 0 |  |
| 388 | 364 | 94% | 347 | 0 | 0% | 0 | 0 |  | 0 | 0 |  |
| 305 | 280 | 92% | 326 | 0 | 0% | 0 | 0 |  | 0 | 0 |  |
| 312 | 293 | 94% | 319 | 0 | 0% | 0 | 0 |  | 0 | 0 |  |
| 362 | 336 | 93% | 272 | 0 | 0% | 0 | 0 |  | 0 | 0 |  |
| 214 | 195 | 91% | 302 | 0 | 0% | 0 | 0 |  | 0 | 0 |  |
| 336 | 317 | 94% | 196 | 0 | 0% | 0 | 0 |  | 0 | 0 |  |
| 261 | 248 | 95% | 254 | 0 | 0% | 0 | 0 |  | 0 | 0 |  |
| 187 | 172 | 92% | 233 | 0 | 0% | 0 | 0 |  | 0 | 0 |  |
| 273.25 | 253 | 92% | 266.58 | 0 | 0% | 0 | 0 | None | 0 | 0 | None |

Note: the data shown in the table represent the average values after three mating experiments. Though egg-laying behavior occurred in females from the mating between *tdrd12* mutant and wide type females, the fertilization rates were zero; on the contrary, *cyp17a1* and double KO mutants could not induce the females’ egg-laying behavior.

Table 2. Primers used in this manuscript.

| *genes* | Primer Sequence | Length |
| --- | --- | --- |
| *b-actin2* | F:CCAGCCTTCCTTCCTGGGTATG  R:GTGGAAGGAGCAAGAGAGGTG | 186bp |
| *dio2* | F:TGGCTTCTTCTCCAACTGCC  R:CTTCGCCCAATTTCACCTGTT | 188bp |
| *ccka* | F:TGCTGGACAAATCAACCTGCT  R:TCTGCGGTATGAGCCTTTGGT | 109bp |
| *igf1* | F:CCCAGGACACCAAAGAAACC  R:ATGCGATAGTTTCTGCCCCC | 107bp |
| *cyp19a1b* | F:AATCAGCTCTTCCAGGTCCC  R:GCTTAGTATAACAGTCTCTTCTCCA | 164bp |
| *nr2e3* | F:CCCCGAACAGGTAGAGAACC  R:ATAGTCTCCCAAACCTGGCAA | 101bp |
| *klf13* | F:TAAGGACGCACACAGGAGAG  R:GGCGTGCATGTTTCATCAGG | 180bp |
| *slc25a18* | F:GGCCGAAAAGAAACTGAGCC  R:TGCCAAACAGTCCAGCATTC | 157bp |
| *egr2a* | F:TCTGGATGAGATTCCCCCGT  R:CTGCTCAGGCCACCTGC | 102bp |
| *gh1* | F:GCGTGCTCATCAAGGGATGTCT  R:GACTGGTCTCCCCTACGGTC | 105bp |
| *sox19a* | F: AGGTGGAAGCCCGAGGAT  R: CTGCTATAGGACATGGGGTTGTAG | 195bp |
| *shisa9b* | F: TCGCTCAAGGCTGTTGCTGAA  R: CTGGGCTGTATGGGTTGGTG | 139bp |
| *npas2* | F:GAGGGAATGGGACACAAGCA  R: TCTCGCCTCTTCTTCTCCGA | 105bp |
| *trh* | F:GTTCGTCTTGGCCTGCGTG  R:CCAGTCCGGCTGATCGTTCTC | 160bp |
| *ddx43* | F: TGGGTGAACTGGAGGAACAGA  R: TTTCGCACCACCTCGACCTATT | 195bp |
| *cyp17a1* | F-genotyping: GCAGTGCTGTTCAGAAGAGCT  R-genotyping: GGCAGTTCATTCTGCTCTGA | 580bp |
| *tdrd12* | F-genotyping: TCCGTGTTTCAGATCGAGGA  R-genotyping: CAGTCCCTCTTTCAGCTCCG | 177bp |

Table 3. Expression of Co-DEGs in the forebrain of behavior- defective mutants.

| ID | Symbol | Description | Log_2_(fc) of double KO vs WT male | Log_2_(fc) of *cyp17a1^-/-^* vs WT male | Log_2_(fc) of *tdrd12^/-^* vs WT male | Log_2_(fc) of WT female vs WT male |
| --- | --- | --- | --- | --- | --- | --- |
| ENSDARG00000094857 | *dio2* | iodothyronine deiodinase 2 | -2.467351 | -2.607247 | 0.155573 | -2.6032 |
| MSTRG.26937 | *cyp19a1b* | cytochrome P450 19A1B | -6.122433 | -6.383161 | 0.03328 | 0.194388 |
| ENSDARG00000091377 | *galn* | galanin/GMAP prepropeptide | -2.04218 | -1.97625 | -0.112948 | -0.541917 |
| ENSDARG00000023287 | *hsd17b3* | hydroxysteroid (17-beta) dehydrogenase 3 | -1.547689 | -1.66322 | 0.088053 | -2.423648 |
| ENSDARG00000098290 | *unc80* | unc-80 homolog (C. elegans) | -1.369823 | -1.420413 | -0.390258 | -0.874142 |
| ENSDARG00000094132 | *igf1* | insulin-like growth factor 1 | -3.408025 | -3.273539 | -0.19902 | -2.52904 |
| ENSDARG00000079348 | *GRIN2B* | glutamate receptor ionotropic, NMDA 2B-like | -1.621188 | -1.46489 | -0.554448 | -1.417438 |
| ENSDARG00000059738 | *ptprsa* | protein tyrosine phosphatase receptor type Sa | -1.152611 | -1.024626 | -0.189101 | -0.581284 |
| ENSDARG00000087709 | *fat3b* | FAT atypical cadherin 3b | -1.765218 | -1.396679 | -0.305786 | -1.564521 |
| ENSDARG00000014717 | *dync1h1* | dynein, cytoplasmic 1, heavy chain 1 | -2.635649 | -1.879402 | -0.572146 | -1.811159 |
| ENSDARG00000014280 | *dlgap1a* | discs, large (Drosophila) homolog-associated protein 1a | -2.293788 | -1.530903 | -0.490419 | -2.003502 |
| ENSDARG00000041926 | *dlg4a* | discs, large homolog 4a (Drosophila) | -1.821377 | -1.433972 | -0.550865 | -1.446456 |
| ENSDARG00000060025 | *nsmfa* | NMDA receptor synaptonuclear signaling and neuronal migration factor a | -1.599141 | -1.907773 | -0.205637 | -1.964003 |
| ENSDARG00000008398 | *cacna1c* | calcium channel, voltage-dependent, L type, alpha 1C subunit | -1.467329 | -1.279615 | -0.386127 | -1.038043 |
| ENSDARG00000061368 | *klf13* | Kruppel-like factor 13 | -2.325825 | -1.824743 | -0.747381 | -2.534131 |
| ENSDARG00000103739 | *bahcc1a* | BAH domain and coiled-coil containing 1a | -2.548527 | -1.63813 | -0.645151 | -2.103527 |
| ENSDARG00000096645 | *si:ch211-131k2.2* | si:ch211-131k2.2 | -4.500459 | -3.605952 | -0.002044 | -4.454944 |
| ENSDARG00000001857 | *aff4* | AF4/FMR2 family, member 4 | -1.598126 | -1.366506 | -0.598787 | -1.867787 |
| ENSDARG00000112909 | *ZNF462* | zinc finger protein 462 | -2.076351 | -1.701792 | -0.818553 | -1.879435 |
| ENSDARG00000074723 | *myo10l1* | myosin X, like 1 | -1.602948 | -1.339052 | -0.769968 | -1.221258 |
| ENSDARG00000098057 | *dscaml1* | Down syndrome cell adhesion molecule like 1 | -1.793935 | -1.306985 | -0.26811 | -1.703338 |
| ENSDARG00000076213 | *DOCK4* | si:dkey-81e3.1 | -1.983417 | -1.249753 | -0.78717 | -1.640212 |
| ENSDARG00000025108 | *magixa* | MAGI family member, X-linked a | -2.085117 | -1.79706 | -0.584963 | -1.726982 |
| ENSDARG00000098631 | *CABZ01044277.1* | XP_021336603.1 dystonin | -2.229913 | -2.514216 | -0.33165 | -1.326937 |
| ENSDARG00000001559 | *csmd2* | CUB and Sushi multiple domains 2 | -1.387978 | -1.070389 | -0.454474 | -1.21661 |
| ENSDARG00000010042 | *dnm1a* | dynamin 1a | -1.528124 | -1.054002 | -0.767234 | -1.305732 |
| ENSDARG00000070810 | *ccka* | cholecystokinin a | -1.788161 | -1.768324 | -0.424532 | -1.713843 |
| ENSDARG00000032197 | *klf12b* | Kruppel-like factor 12b | -2.6597 | -1.889488 | -0.685374 | -2.889488 |
| ENSDARG00000076697 | *SIPA1* | zmp:0000001168 | -2.730769 | -1.77613 | -0.80477 | -1.941189 |
| ENSDARG00000078902 | *rims1b* | regulating synaptic membrane exocytosis 1b | -1.258096 | -1.171987 | -0.444651 | -1.077621 |
| ENSDARG00000063307 | *sgsm2* | small G protein signaling modulator 2 | -1.676795 | -1.278528 | -0.48228 | -1.854739 |
| MSTRG.26936 | *cyp19a1* | cytochrome P450, family 19, subfamily A, polypeptide 1b isoform X1 | -1.14543 | -1.675251 | 0.23644 | -0.0813 |
| ENSDARG00000088898 | *caln1* | calneuron 1 | -1.95793 | -1.443956 | -0.687256 | -1.904963 |
| ENSDARG00000098683 | *zmiz2* | zinc finger, MIZ-type containing 2 | -1.059205 | -1.061001 | -0.430048 | -1.547267 |
| ENSDARG00000074258 | *gramd1bb* | GRAM domain containing 1Bb | -1.602014 | -1.467458 | -0.645544 | -2.143795 |
| ENSDARG00000009738 | *cacnb4a* | calcium channel, voltage-dependent, beta 4a subunit | -2.596886 | -1.959028 | -0.92106 | -2.130397 |
| ENSDARG00000004789 | *lrp1ba* | low density lipoprotein receptor-related protein 1Ba | -2.286304 | -1.643856 | -0.842956 | -1.483985 |
| ENSDARG00000016868 | *rhobtb4* | Rho related BTB domain containing 4 | -1.927991 | -1.488257 | -0.647297 | -2.335079 |
| ENSDARG00000078187 | *slc12a5b* | solute carrier family 12 member 5b | -1.612559 | -1.111715 | -0.538921 | -1.595779 |
| ENSDARG00000075230 | *tet1* | tet methylcytosine dioxygenase 1 | -2.008613 | -1.731727 | -0.538488 | -2.235813 |
| ENSDARG00000088307 | *mrtfba* | myocardin related transcription factor Ba | -2.395767 | -1.818887 | -0.865563 | -2.363883 |
| ENSDARG00000009459 | *slc35e4* | solute carrier family 35 member E4 | -1.419034 | -1.600006 | -0.755542 | -1.710766 |
| MSTRG.33980 | *Insyn2b* | PREDICTED: mucin-5AC-like isoform X1 | -1.348742 | -1.1065 | -0.589021 | -1.473231 |
| ENSDARG00000060941 | *ANKFN1* | si:ch211-277e21.2 | -2.188114 | -1.932587 | -0.520689 | -3.173215 |
| ENSDARG00000087260 | *mtss1lb* | MTSS I-BAR domain containing 2b | -2.730834 | -1.823943 | -0.678654 | -1.773317 |
| ENSDARG00000097017 | *mical3a* | microtubule associated monooxygenase, calponin and LIM domain containing 3a | -1.923121 | -1.28844 | -0.741775 | -1.368834 |
| ENSDARG00000016348 | *dip2bb* | disco-interacting protein 2 homolog Bb | -1.62242 | -1.289357 | -0.575581 | -1.075913 |
| ENSDARG00000059888 | *ago3a* | argonaute RISC catalytic component 3a | -2.099217 | -2.080234 | -1.024739 | -2.67941 |
| ENSDARG00000074819 | *si:dkeyp-23e4.3* | si:dkeyp-23e4.3 | -1.368114 | -1.53906 | 0.409493 | -2.844996 |
| MSTRG.6383 | *LBH* | protein LBH | -2.266028 | -1.988831 | -1.302438 | -1.575649 |
| ENSDARG00000077656 | *arfgef3* | ARFGEF family member 3 | -1.638056 | -1.096118 | -0.713442 | -1.268681 |
| ENSDARG00000054253 | *sobpa* | sine oculis binding protein homolog (Drosophila) a | -2.165748 | -1.745357 | -0.836331 | -2.087463 |
| ENSDARG00000077905 | *csmd3a* | CUB and Sushi multiple domains 3a | -1.488993 | -1.467725 | -0.369128 | -1.240314 |
| ENSDARG00000060875 | *pde1a* | phosphodiesterase 1A, calmodulin-dependent | -1.677876 | -1.283665 | -0.629996 | -1.292455 |
| ENSDARG00000098511 | *ntrk2b* | neurotrophic tyrosine kinase, receptor, type 2b | -3.204801 | -1.861162 | -0.915531 | -2.373099 |
| ENSDARG00000060539 | *shank1* | SH3 and multiple ankyrin repeat domains 1 | -2.013806 | -1.521953 | -0.462763 | -1.661677 |
| ENSDARG00000068286 | *slc7a4* | solute carrier family 7 member 4 | -2.021342 | -1.783977 | -1.045189 | -1.845457 |
| ENSDARG00000005670 | *ttbk2a* | tau tubulin kinase 2a | -2.006848 | -1.395504 | -0.863231 | -1.561554 |
| ENSDARG00000029668 | *crim1* | cysteine rich transmembrane BMP regulator 1 (chordin-like) | -1.383329 | -1.254183 | -0.470339 | -0.799975 |
| ENSDARG00000076702 | *CABZ01068356.1* | PH and SEC7 domain-containing protein 1-like | -2.047148 | -1.456134 | -1.024931 | -1.674352 |
| ENSDARG00000061992 | *dot1l* | DOT1-like histone H3K79 methyltransferase | -1.859325 | -1.502724 | -0.788008 | -1.646714 |
| ENSDARG00000052642 | *shisa9b* | shisa family member 9b | -1.958363 | -1.811842 | -0.814091 | -1.360195 |
| ENSDARG00000102406 | *si:ch211-165e15.1* | si:ch211-165e15.1 | -2.694721 | -2.275567 | -0.533064 | -1.218154 |
| ENSDARG00000006923 | *cacna1ab* | calcium channel, voltage-dependent, P/Q type, alpha 1A subunit, b | -1.386214 | -1.207392 | -0.652524 | -0.604151 |
| ENSDARG00000020718 | *slc25a22a* | solute carrier family 25 member 22a | -1.969812 | -1.273 | -0.994888 | -1.791962 |
| ENSDARG00000002816 | *rasgrf2b* | Ras protein-specific guanine nucleotide-releasing factor 2b | -2.193036 | -1.443607 | -0.838311 | -1.621699 |
| ENSDARG00000061541 | *unc5db* | unc-5 netrin receptor Db | -1.522546 | -1.230755 | -0.564472 | -2.18028 |
| ENSDARG00000043332 | *prkcz* | protein kinase C, zeta | -1.695413 | -1.317477 | -0.488534 | -1.521335 |
| ENSDARG00000073822 | *IQSEC1* | IQ motif and Sec7 domain ArfGEF 1a | -2.033349 | -1.689103 | -0.772459 | -2.240295 |
| ENSDARG00000112431 | *LO018598.1* | NP_001233205.1 reelin precursor | -2.2226 | -2.057855 | -0.45912 | -1.171172 |
| ENSDARG00000043226 | *nfixa* | nuclear factor I/Xa | -2.627347 | -1.846218 | -1.119134 | -2.640095 |
| ENSDARG00000043646 | *slc6a8* | solute carrier family 6 member 8 | -2.171439 | -1.755039 | -0.63454 | -2.305674 |
| MSTRG.21108 | *SHC3* | SHC-transforming protein 3 | -1.488541 | -1.030882 | -0.571141 | -1.710544 |
| ENSDARG00000061042 | *gabbr2* | gamma-aminobutyric acid (GABA) B receptor, 2 | -1.703238 | -1.022543 | -0.588192 | -1.57981 |
| ENSDARG00000058284 | *CT990561.1* | EPH receptor A5 | -1.818582 | -1.561194 | -0.787915 | -1.973092 |
| ENSDARG00000102082 | *nr3c2* | nuclear receptor subfamily 3, group C, member 2 | -1.643035 | -1.038829 | -1.487618 | -1.386855 |
| ENSDARG00000055565 | *cacnb2b* | calcium channel, voltage-dependent, beta 2b | -1.184525 | -1.035968 | -0.71122 | -1.308634 |
| ENSDARG00000102067 | *grm5b* | glutamate receptor, metabotropic 5b | -2.73543 | -1.876391 | -1.018556 | -2.267651 |
| ENSDARG00000104810 | *akt3a* | v-akt murine thymoma viral oncogene homolog 3a | -1.228075 | -1.034433 | -0.809057 | -1.425662 |
| ENSDARG00000045453 | *f13a1a.1* | coagulation factor XIII, A1 polypeptide a, tandem duplicate 1 | -9.291554 | -9.291554 | -1.773229 | 3.743474 |
| ENSDARG00000017365 | *slc23a2* | solute carrier family 23 member 2 | -1.88907 | -1.50462 | -0.755852 | -1.88907 |
| ENSDARG00000005834 | *gatad2b* | GATA zinc finger domain containing 2B | -2.055322 | -1.088936 | -0.75773 | -1.721204 |
| ENSDARG00000094512 | *gabrz* | gamma-aminobutyric acid (GABA) A receptor, zeta | -2.075733 | -1.606456 | -0.683594 | -2.119483 |
| ENSDARG00000060597 | *zgc:158659* | zgc:158659 | -1.2205 | -1.64291 | -0.260764 | -1.40874 |
| ENSDARG00000063180 | *dock3* | dedicator of cytokinesis 3 | -1.840073 | -1.138071 | -0.631389 | -1.837366 |
| ENSDARG00000068582 | *rnf44* | ring finger protein 44 | -1.54407 | -1.23588 | -0.529246 | -2.029146 |
| ENSDARG00000110950 | *CU929544.1* | receptor-type tyrosine-protein phosphatase delta-like | -1.424768 | -1.401976 | 0.199474 | -0.331372 |
| ENSDARG00000053326 | *gna11b* | guanine nucleotide binding protein (G protein), alpha 11b (Gq class) | -1.680233 | -1.245697 | -0.86605 | -1.763792 |
| ENSDARG00000005586 | *zbtb20* | zinc finger and BTB domain containing 20 | -4.18133 | -2.944291 | -0.789012 | -3.596367 |
| ENSDARG00000060148 | *sh3pxd2aa* | SH3 and PX domains 2Aa | -1.397335 | -1.049831 | -0.717798 | -1.00446 |
| ENSDARG00000098906 | *herc7* | hect domain and RLD 7 | -3.525628 | -3.729642 | -1.278535 | -2.085056 |
| ENSDARG00000008575 | *lyst* | lysosomal trafficking regulator | -1.466293 | -1.061729 | -0.463687 | -1.334255 |
| ENSDARG00000075271 | *rapgef5a* | Rap guanine nucleotide exchange factor (GEF) 5a | -1.747234 | -1.37468 | -0.86509 | -1.567528 |
| ENSDARG00000105178 | *erc2* | ELKS/RAB6-interacting/CAST family member 2 | -2.375039 | -1.547488 | -0.865577 | -2.08187 |
| ENSDARG00000109726 | *PLCB1* | si:ch1073-140o9.2 | -2.039369 | -1.527162 | -0.586402 | -0.827999 |
| ENSDARG00000079741 | *CSRNP3* | cysteine-serine-rich nuclear protein 3 | -1.986809 | -1.623461 | -0.575277 | -1.67613 |
| ENSDARG00000098349 | *hdac4* | histone deacetylase 4 | -1.358531 | -1.202056 | -0.592815 | -1.631485 |
| ENSDARG00000074064 | *rem2* | RAS (RAD and GEM)-like GTP binding 2 | -1.375367 | -1.120294 | -0.834746 | -1.41329 |
| ENSDARG00000075539 | *snphb* | syntaphilin b | -1.480122 | -1.006905 | -0.41635 | -0.639618 |
| ENSDARG00000032039 | *mxd1* | MAX dimerization protein 1 | -2.487731 | -1.500417 | -1.008532 | -2.298598 |
| ENSDARG00000027497 | *mast1a* | microtubule associated serine/threonine kinase 1a | -1.964587 | -1.219922 | -0.796271 | -1.570203 |
| ENSDARG00000016866 | *fam102ab* | family with sequence similarity 102 member Ab | -1.298416 | -1.205222 | -0.511348 | -1.781946 |
| ENSDARG00000003989 | *crhr1* | corticotropin releasing hormone receptor 1 | -1.594111 | -1.139694 | -0.699756 | -1.556072 |
| ENSDARG00000063264 | *pcdh9* | protocadherin 9 | -1.609625 | -1.138482 | -0.617106 | -1.778306 |
| ENSDARG00000079756 | *peak1* | pseudopodium-enriched atypical kinase 1 | -1.719892 | -1.231006 | -0.93523 | -1.410139 |
| ENSDARG00000071230 | *lrfn5a* | leucine rich repeat and fibronectin type III domain containing 5a | -1.70056 | -1.129734 | -0.693543 | -1.622969 |
| ENSDARG00000077920 | *csmd3a* | CUB and Sushi multiple domains 3a | -1.378512 | -1.442642 | -0.616671 | -1.281487 |
| ENSDARG00000100007 | *arhgap39* | Rho GTPase activating protein 39 | -1.006789 | -1.009266 | -0.544885 | -1.385177 |
| ENSDARG00000092260 | *si:ch73-290k24.6* | si:ch73-290k24.6 | -1.074859 | -1.055373 | -0.285718 | -1.029796 |
| ENSDARG00000063732 | *pde11a* | phosphodiesterase 11a | -1.59761 | -1.204515 | -0.453933 | -1.885907 |
| ENSDARG00000088967 | *wnk4b* | WNK lysine deficient protein kinase 4b | -1.494987 | -1.217109 | -0.227083 | -1.651204 |
| ENSDARG00000008372 | *spred2b* | sprouty related EVH1 domain containing 2b | -1.663737 | -1.202972 | -0.768522 | -1.573687 |
| ENSDARG00000067815 | *CABZ01060891.1* | leucine-rich repeat and immunoglobulin-like domain-containing nogo receptor-interacting protein 3 | -3.666542 | -2.33918 | -1.400109 | -2.383235 |
| ENSDARG00000098612 | *chrm2a* | cholinergic receptor, muscarinic 2a | -2.685657 | -2.617752 | -0.7164 | -3.568843 |
| ENSDARG00000069262 | *erich3* | glutamate-rich 3 | -1.774565 | -1.715023 | -0.034765 | -1.811507 |
| ENSDARG00000091408 | *mtcl1* | microtubule crosslinking factor 1 | -2.249809 | -1.759281 | -0.826597 | -1.911284 |
| ENSDARG00000012823 | *dlgap4b* | discs, large (Drosophila) homolog-associated protein 4b | -1.359013 | -1.111156 | -0.704275 | -1.665044 |
| ENSDARG00000003449 | *pde10a* | phosphodiesterase 10A | -1.776638 | -1.427053 | -0.583993 | -1.143726 |
| ENSDARG00000003544 | *adarb1b* | adenosine deaminase RNA specific B1b | -2.139064 | -1.851901 | -0.820575 | -1.858956 |
| ENSDARG00000091130 | *kcnq2b* | potassium voltage-gated channel, KQT-like subfamily, member 2b | -1.907259 | -1.226081 | -0.761488 | -1.469599 |
| ENSDARG00000013020 | *dtnbb* | dystrobrevin, beta b | -1.868094 | -1.199831 | -0.767735 | -1.809612 |
| ENSDARG00000060372 | *plxna2* | plexin A2 | -2.193788 | -1.145794 | -0.629305 | -1.60781 |
| ENSDARG00000016536 | *npas2* | neuronal PAS domain protein 2 | -1.852652 | -1.63026 | -0.704056 | -1.880133 |
| ENSDARG00000075685 | *tlr7* | toll-like receptor 7 | -1.587848 | -1.048363 | -0.705915 | -1.671075 |
| ENSDARG00000071524 | *insrb* | insulin receptor b | -1.497141 | -1.036146 | -0.44643 | -1.448805 |
| ENSDARG00000027423 | *igf1ra* | insulin-like growth factor 1a receptor | -1.323276 | -1.076058 | -0.494887 | -1.098958 |
| ENSDARG00000114067 | *tspan17* | tetraspanin 17 | -1.097483 | -1.014451 | -0.779663 | -1.218669 |
| ENSDARG00000102584 | *cpne7* | copine VII | -1.854617 | -1.211138 | -0.625505 | -1.395471 |
| ENSDARG00000069026 | *adcy1a* | adenylate cyclase 1a | -2.22896 | -1.380176 | -1.092102 | -2.009102 |
| ENSDARG00000058598 | *sox18* | SRY-box transcription factor 18 | -1.52934 | -1.108765 | -0.003368 | -1.659925 |
| ENSDARG00000098215 | *BICD2* | zgc:171572 | -2.081233 | -1.299739 | -0.965567 | -1.784594 |
| ENSDARG00000036826 | *ankrd52a* | ankyrin repeat domain 52a | -2.683411 | -1.771638 | -0.765154 | -2.271433 |
| ENSDARG00000036998 | *arf3b* | ADP-ribosylation factor 3b | -2.711163 | -1.488082 | -0.895561 | -2.164501 |
| ENSDARG00000042518 | *hipk2* | homeodomain interacting protein kinase 2 | -2.027415 | -1.455195 | -0.807992 | -2.243479 |
| ENSDARG00000036848 | *slc43a2a* | solute carrier family 43 member 2a | -1.931229 | -1.341728 | 0.085042 | -2.778681 |
| MSTRG.15506 | *AKAP9* | A-kinase anchor protein 9 | -1.206243 | -1.578211 | 0.041356 | -0.366248 |
| ENSDARG00000075296 | *fam83c* | family with sequence similarity 83 member C | -2.217052 | -1.383871 | -0.732218 | -1.973495 |
| ENSDARG00000011809 | *mical1* | microtubule associated monooxygenase, calponin and LIM domain containing 1 | -1.263701 | -1.019005 | -0.760103 | -0.906474 |
| ENSDARG00000070620 | *grin2db* | glutamate receptor, ionotropic, N-methyl D-aspartate 2D, b | -1.617811 | -1.303247 | -0.952915 | -0.894551 |
| ENSDARG00000009493 | *MAP3K13* | si:ch211-45c16.2 | -2.536571 | -1.755211 | -0.670322 | -2.144254 |
| ENSDARG00000078327 | *CEP170B* | si:ch73-212j7.1 | -1.578205 | -1.1814 | -0.802987 | -1.608866 |
| ENSDARG00000075139 | *hdac5* | histone deacetylase 5 | -1.83662 | -1.041011 | -0.703047 | -2.079254 |
| ENSDARG00000061635 | *myo5aa* | myosin VAa | -2.418501 | -1.323226 | -0.919269 | -2.224123 |
| ENSDARG00000020057 | *bmpr2b* | bone morphogenetic protein receptor, type II b (serine/threonine kinase) | -1.920131 | -1.212625 | -0.889757 | -1.529276 |
| ENSDARG00000035966 | *pgr* | progesterone receptor | -2.600464 | -2.389567 | -0.044738 | -0.366666 |
| ENSDARG00000076292 | *tns2a* | tensin 2a | -1.893085 | -1.358048 | -0.4168 | -2.33787 |
| ENSDARG00000104005 | *slc4a3* | solute carrier family 4 member 3 | -2.122995 | -1.261174 | -1.021883 | -1.731804 |
| MSTRG.14233 | *UNC13C* | protein unc-13 homolog C-like isoform X2 | -2.062736 | -1.237176 | -0.459192 | -2.103378 |
| MSTRG.18936 | *Hcn2* | potassium/sodium hyperpolarization-activated cyclic nucleotide-gated channel 2-like | -1.564905 | -1.1976 | -0.617056 | -2.145605 |
| ENSDARG00000104283 | *cdc42bpaa* | CDC42 binding protein kinase alpha (DMPK-like) a | -2.707274 | -1.66342 | -1.547278 | -1.857299 |
| ENSDARG00000061231 | *tinagl1* | tubulointerstitial nephritis antigen-like 1 | -2.413703 | -1.662298 | -0.434121 | -3.919765 |
| ENSDARG00000055672 | *map3k10* | mitogen-activated protein kinase kinase kinase 10 | -1.576238 | -1.133509 | -1.015081 | -1.610185 |
| MSTRG.12484 | *Dync1h1* | cytoplasmic dynein 1 heavy chain 1 | -1.86342 | -1.166615 | -0.476487 | -1.612579 |
| ENSDARG00000017108 | *kcna1b* | potassium voltage-gated channel, shaker-related subfamily, member 1b | -1.737974 | -1.446634 | -0.527158 | -1.631059 |
| ENSDARG00000061718 | *znf704* | zinc finger protein 704 | -2.779341 | -3.57289 | -2.309855 | -1.738313 |
| ENSDARG00000098857 | *lifra* | LIF receptor subunit alpha a | -1.905028 | -1.128584 | -0.703394 | -1.919057 |
| ENSDARG00000073769 | *magi2b* | membrane associated guanylate kinase, WW and PDZ domain containing 2b | -2.112768 | -1.671578 | -0.821663 | -1.411426 |
| ENSDARG00000059523 | *rims4* | regulating synaptic membrane exocytosis 4 | -1.918062 | -1.314094 | -0.477281 | -1.71703 |
| ENSDARG00000052604 | *cpeb2* | cytoplasmic polyadenylation element binding protein 2 | -1.659783 | -1.285579 | -0.444116 | -1.402351 |
| ENSDARG00000077582 | *ank3b* | ankyrin 3b | -1.873376 | -1.587152 | -0.934776 | -0.583869 |
| ENSDARG00000055223 | *ablim2* | actin binding LIM protein family, member 2 | -1.334602 | -1.070389 | -0.437172 | -1.012674 |
| ENSDARG00000000563 | *ttn.1* | titin, tandem duplicate 1 | -1.662965 | -1.925999 | -0.247928 | -1.925999 |
| ENSDARG00000089292 | *adgrl1a* | adhesion G protein-coupled receptor L1a | -1.716207 | -1.289507 | -0.833246 | -1.156445 |
| ENSDARG00000069560 | *kcnh3* | potassium voltage-gated channel, subfamily H (eag-related), member 3 | -1.875542 | -1.146152 | -0.829074 | -1.906569 |
| ENSDARG00000062134 | *kcnab2b* | potassium voltage-gated channel, shaker-related subfamily, beta member 2 b | -1.421087 | -1.231982 | -0.151556 | -1.829404 |
| ENSDARG00000100032 | *GABRB2* | gamma-aminobutyric acid type A receptor beta2 subunit | -2.509298 | -2.139725 | -1.388162 | -2.223626 |
| ENSDARG00000099841 | *prkcab* | protein kinase C, alpha, b | -1.748193 | -1.036698 | -0.826947 | -1.739204 |
| ENSDARG00000078954 | *rxrba* | retinoid x receptor, beta a | -1.256648 | -1.004932 | -0.212476 | -1.408487 |
| ENSDARG00000101019 | *ext1b* | exostosin glycosyltransferase 1b | -1.792022 | -1.38247 | -0.675071 | -2.349865 |
| ENSDARG00000075383 | *gramd1ba* | GRAM domain containing 1Ba | -1.396488 | -1.274851 | -0.23259 | -1.671141 |
| ENSDARG00000027903 | *adamtsl3* | ADAMTS-like 3 | -2.266787 | -1.200697 | -0.669851 | -1.916289 |
| ENSDARG00000101687 | *CU929259.1* | microtubule-associated protein 4-like | -2.174912 | -1.237556 | -0.537774 | -1.681334 |
| ENSDARG00000060309 | *srgap3* | SLIT-ROBO Rho GTPase activating protein 3 | -1.736605 | -1.033947 | -0.852007 | -1.258928 |
| ENSDARG00000063730 | *osbpl6* | oxysterol binding protein-like 6 | -1.543543 | -2.516576 | -0.724569 | -1.82852 |
| ENSDARG00000060741 | *syt17* | synaptotagmin XVII | -1.357261 | -1.40576 | -1.064236 | -1.744743 |
| ENSDARG00000098984 | *pid1* | phosphotyrosine interaction domain containing 1 | -1.771449 | -1.216985 | -0.78834 | -1.997009 |
| ENSDARG00000100620 | *taok2b* | TAO kinase 2b | -1.572062 | -1.172627 | -0.558215 | -1.619939 |
| ENSDARG00000005394 | *dnmt3aa* | DNA (cytosine-5-)-methyltransferase 3 alpha a | -2.004558 | -1.325345 | -0.749918 | -2.13175 |
| ENSDARG00000052766 | *EVI5L* | si:ch211-239f4.1 | -1.306941 | -1.459432 | -1.068972 | -1.302563 |
| ENSDARG00000052881 | *dnajb5* | DnaJ heat shock protein family (Hsp40) member B5 | -1.452739 | -1.186975 | -0.466246 | -1.889803 |
| ENSDARG00000005783 | *ncanb* | neurocan b | -1.993746 | -1.220305 | -0.487434 | -1.3581 |
| ENSDARG00000058248 | *si:dkeyp-77h1.4* | si:dkeyp-77h1.4 | -1.89072 | -1.231833 | -0.801384 | -1.283179 |
| MSTRG.12423 | *RYR3* | PREDICTED: ryanodine receptor 3-like | -1.150429 | -1.284789 | -0.568697 | -0.59152 |
| ENSDARG00000102142 | *ece2b* | endothelin converting enzyme 2b | -2.130876 | -1.231277 | -0.663749 | -1.976934 |
| ENSDARG00000025206 | *syt2a* | synaptotagmin IIa | -2.271688 | -2.302882 | -1.094068 | -2.752493 |
| ENSDARG00000092624 | *sptbn4b* | spectrin, beta, non-erythrocytic 4b | -1.78182 | -1.310929 | -0.350267 | -0.853159 |
| ENSDARG00000076014 | *ERC1* | si:dkey-222l13.1 | -2.559792 | -1.356975 | -1.006942 | -2.221623 |
| ENSDARG00000104981 | *SLC45A4* | solute carrier family 45 member 4-like | -3.045643 | -1.829151 | -1.080408 | -2.759888 |
| ENSDARG00000075532 | *slc4a11* | solute carrier family 4 member 11 | -1.244188 | -1.027721 | -0.808603 | -1.643544 |
| ENSDARG00000069608 | *palm2akap2* | palm2 and akap2 fusion | -1.741688 | -1.350497 | -0.541671 | -2.189761 |
| MSTRG.29660 | *SIFaR* | neuropeptide FF receptor 2-like | -1.502902 | -1.46245 | -0.580145 | -1.957215 |
| ENSDARG00000058015 | *ano3* | anoctamin 3 | -2.192983 | -1.169728 | -0.900201 | -2.023058 |
| MSTRG.15433 | *RELN* | reelin precursor | -2.304087 | -1.331206 | -0.347725 | -1.04981 |
| ENSDARG00000005216 | *zgc:158328* | zgc:158328 | -3.512568 | -1.982053 | -1.340101 | -3.559111 |
| ENSDARG00000018896 | *mmp24* | matrix metallopeptidase 24 | -2.13593 | -1.640835 | -0.597185 | -2.399477 |
| MSTRG.16736 | *ADGRB2* | adhesion G protein-coupled receptor B2-like isoform X2 | -1.523365 | -1.500928 | -0.416612 | -1.496482 |
| ENSDARG00000023914 | *mark4a* | MAP/microtubule affinity-regulating kinase 4a | -1.79185 | -1.030362 | -0.618222 | -2.116233 |
| ENSDARG00000035308 | *grb10b* | growth factor receptor-bound protein 10b | -1.418373 | -1.510296 | -0.76387 | -1.234661 |
| ENSDARG00000037069 | *zdhhc14* | zinc finger DHHC-type containing 14 | -1.59323 | -1.136515 | -0.767515 | -1.338511 |
| ENSDARG00000020242 | *fermt2* | fermitin family member 2 | -1.953206 | -2.577057 | -0.479889 | -1.984233 |
| ENSDARG00000075815 | *slc12a5a* | solute carrier family 12 member 5a | -2.819624 | -1.545524 | -0.825516 | -2.229444 |
| ENSDARG00000018096 | *scap* | SREBF chaperone | -1.406715 | -1.171611 | -0.493539 | -1.700199 |
| ENSDARG00000078011 | *NAV1* | neuron navigator 1a | -1.596734 | -1.113525 | -0.735013 | -1.553078 |
| ENSDARG00000062550 | *rc3h1a* | ring finger and CCCH-type domains 1a | -1.6537 | -1.099388 | -0.703282 | -1.643985 |
| ENSDARG00000031702 | *prkg1b* | protein kinase cGMP-dependent 1b | -1.096488 | -1.056457 | -0.305413 | -1.392021 |
| ENSDARG00000053804 | *osbpl2a* | oxysterol binding protein-like 2a | -2.252463 | -1.913375 | -1.051366 | -2.258642 |
| ENSDARG00000007398 | *lrrk1* | leucine-rich repeat kinase 1 | -1.32061 | -1.627791 | -0.777984 | -0.64007 |
| ENSDARG00000086977 | *atxn1l* | ataxin 1-like | -3.115477 | -3.06301 | -1.128898 | -2.089005 |
| ENSDARG00000022614 | *ddx43* | DEAD (Asp-Glu-Ala-Asp) box polypeptide 43 | -4.336283 | -3.995246 | -1.024005 | -0.093427 |
| ENSDARG00000070504 | *trpc5a* | transient receptor potential cation channel, subfamily C, member 5a | -2.10172 | -1.391227 | -0.71387 | -2.010954 |
| ENSDARG00000059939 | *dab1a* | DAB adaptor protein 1a | -1.641106 | -1.272383 | -0.487122 | -1.788419 |
| ENSDARG00000042492 | *si:dkey-250d21.1* | si:dkey-250d21.1 | -2.551796 | -1.647823 | -1.003733 | -1.573822 |
| ENSDARG00000017742 | *grm6a* | glutamate receptor, metabotropic 6a | -2.140481 | -1.683436 | -0.140481 | -1.608958 |
| ENSDARG00000061248 | *xylt1* | xylosyltransferase I | -1.79023 | -1.103311 | -0.942515 | -1.081428 |
| ENSDARG00000012833 | *foxn3* | forkhead box N3 | -1.65238 | -1.185681 | -0.848847 | -1.749056 |
| MSTRG.15448 | *RELN* | reelin precursor | -1.379493 | -1.10164 | -0.548621 | -1.171766 |
| ENSDARG00000043835 | *rab3ab* | RAB3A, member RAS oncogene family, b | -2.146299 | -1.014114 | -0.895853 | -2.018145 |
| ENSDARG00000041239 | *wdr20b* | WD repeat domain 20b | -2.079289 | -1.726715 | -0.067114 | -0.742763 |
| ENSDARG00000058848 | *mcoln1b* | mucolipin 1b | -1.67553 | -1.492569 | -0.911191 | -1.460704 |
| ENSDARG00000080006 | *tmem41b* | transmembrane protein 41B | -1.409032 | -1.594844 | -0.482803 | -1.522332 |
| ENSDARG00000102610 | *r3hdm2* | R3H domain containing 2 | -1.810649 | -1.367042 | -0.292617 | -2.075993 |
| ENSDARG00000011703 | *clocka* | clock circadian regulator a | -1.48134 | -1.045034 | -0.738672 | -1.216719 |
| ENSDARG00000078839 | *LRRTM4* | si:dkey-28o19.1 | -2.450329 | -1.454873 | -1.189565 | -1.768859 |
| ENSDARG00000017220 | *otud7b* | OTU deubiquitinase 7B | -1.534963 | -1.219367 | -0.13714 | -1.732128 |
| ENSDARG00000093091 | *iqsec3b* | IQ motif and Sec7 domain ArfGEF 3b | -1.675423 | -1.181184 | -0.660842 | -1.294159 |
| ENSDARG00000036626 | *sema6ba* | sema domain, transmembrane domain (TM), and cytoplasmic domain, (semaphorin) 6Ba | -2.452512 | -1.607683 | -0.833161 | -2.208587 |
| ENSDARG00000090421 | *SH2B2* | SH2B adaptor protein 2 | -1.563235 | -1.152246 | -0.440807 | -1.18174 |
| ENSDARG00000032761 | *pde4d* | phosphodiesterase 4D, cAMP-specific | -2.006826 | -1.344478 | -1.002921 | -1.938113 |
| ENSDARG00000013828 | *snx13* | sorting nexin 13 | -1.388442 | -1.137309 | -0.578187 | -1.091106 |
| ENSDARG00000005126 | *large1* | LARGE xylosyl- and glucuronyltransferase 1 | -2.025535 | -1.597674 | -0.484162 | -1.991588 |
| ENSDARG00000116353 | *lrp4* | low density lipoprotein receptor-related protein 4 | -1.395833 | -1.080119 | -0.729734 | -1.797421 |
| ENSDARG00000090617 | *ctif* | CBP80/20-dependent translation initiation factor | -1.843791 | -1.483322 | -0.866011 | -1.244203 |
| ENSDARG00000019963 | *sfxn1* | sideroflexin 1 | -1.996238 | -1.457079 | -0.460963 | -1.796566 |
| ENSDARG00000103420 | *si:dkey-56m15.5* | si:dkey-56m15.5 | -1.832123 | -1.922321 | -0.327053 | -1.467126 |
| ENSDARG00000077686 | *aak1b* | AP2 associated kinase 1b | -2.344019 | -1.057629 | -0.917257 | -2.268949 |
| ENSDARG00000077761 | *nlgn4xb* | neuroligin 4 X-linked b | -1.775568 | -2.103623 | -0.429393 | -1.692427 |
| ENSDARG00000101280 | *vat1l* | vesicle amine transport 1-like | -1.499791 | -1.088259 | -0.462265 | -1.371594 |
| ENSDARG00000018967 | *gabbr1a* | gamma-aminobutyric acid (GABA) B receptor, 1a | -1.443319 | -1.78001 | -0.9366 | -0.851843 |
| ENSDARG00000036985 | *plxnb2b* | plexin b2b | -1.177787 | -1.011998 | -0.442174 | -0.510738 |
| ENSDARG00000063332 | *shank3a* | SH3 and multiple ankyrin repeat domains 3a | -1.605721 | -1.838606 | -0.651236 | -1.035771 |
| ENSDARG00000079805 | *tagln3a* | transgelin 3a | -2.039803 | -1.324229 | -0.741926 | -1.386669 |
| ENSDARG00000025797 | *abhd2a* | abhydrolase domain containing 2a | -2.130288 | -1.580949 | -0.395083 | -2.902878 |
| ENSDARG00000041086 | *LO018380.1* | sodium/potassium/calcium exchanger 3-like | -1.655314 | -1.338624 | -0.502123 | -0.660075 |
| ENSDARG00000087596 | *si:ch73-95l15.5* | si:ch73-95l15.5 | -1.110703 | -1.745186 | 0.251375 | -1.29956 |
| ENSDARG00000094327 | *SPATA1* | si:dkey-205k8.5 | -1.223576 | -1.048134 | 0.118115 | -1.647856 |
| ENSDARG00000100163 | *tox4b* | TOX high mobility group box family member 4 b | -1.376902 | -1.593036 | -0.909509 | -1.779449 |
| ENSDARG00000019588 | *chico* | chico | -1.859908 | -1.328103 | -0.778378 | -2.174168 |
| MSTRG.4521 | *TMEM63B* | CSC1-like protein 2 isoform X3 | -3.104941 | -1.95099 | -0.902177 | -2.085576 |
| ENSDARG00000078775 | *RASA2* | RAS p21 protein activator 2 | -2.255712 | -1.364184 | -0.867896 | -1.438759 |
| ENSDARG00000091619 | *mvb12ba* | multivesicular body subunit 12Ba | -1.584146 | -1.379002 | -0.737783 | -1.29843 |
| ENSDARG00000063079 | *ago3b* | argonaute RISC catalytic component 3b | -1.651654 | -1.023896 | -0.897111 | -1.136615 |
| MSTRG.1594 | *Sstr5* | somatostatin receptor type 5-like isoform X2 | -1.556072 | -1.072945 | -0.673338 | -1.239517 |
| ENSDARG00000061471 | *sema5ba* | sema domain, seven thrombospondin repeats (type 1 and type 1-like), transmembrane domain (TM) and short cytoplasmic domain, (semaphorin) 5Ba | -1.697542 | -1.043186 | -0.424091 | -1.509609 |
| ENSDARG00000013491 | *map3k9* | mitogen-activated protein kinase kinase kinase 9 | -1.792684 | -1.190119 | -0.652582 | -0.736572 |
| ENSDARG00000062195 | *fbxo31* | F-box protein 31 | -1.168673 | -1.008933 | -0.519671 | -0.873264 |
| ENSDARG00000102095 | *si:ch73-233f7.5* | si:ch73-233f7.5 | -1.958015 | -1.03137 | -0.760652 | -2.124623 |
| ENSDARG00000062305 | *rims3* | regulating synaptic membrane exocytosis 3 | -2.484162 | -1.449186 | -1.164005 | -2.683143 |
| ENSDARG00000090496 | *thsd7ab* | thrombospondin, type I, domain containing 7Ab | -2.530515 | -1.86755 | -0.405819 | -1.591915 |
| ENSDARG00000009899 | *znf385a* | zinc finger protein 385A | -1.481996 | -1.205319 | -0.199672 | -1.859822 |
| ENSDARG00000100908 | *lcor* | ligand dependent nuclear receptor corepressor | -2.641106 | -1.989029 | -1.396798 | -2.59048 |
| ENSDARG00000075754 | *mri1* | methylthioribose-1-phosphate isomerase 1 | -1.480884 | -1.057727 | -0.602276 | -2.181934 |
| ENSDARG00000107408 | *TCF4* | transcription factor 4 | -1.924925 | -1.299698 | -0.73846 | -1.835861 |
| ENSDARG00000036546 | *pdxkb* | pyridoxal (pyridoxine, vitamin B6) kinase b | -1.864039 | -1.248163 | -0.99561 | -1.775366 |
| ENSDARG00000068421 | *ttc9b* | tetratricopeptide repeat domain 9B | -1.339052 | -1.26559 | -0.646252 | -1.36732 |
| ENSDARG00000075567 | *fbxl20* | F-box and leucine-rich repeat protein 20 | -1.30956 | -1.512293 | -0.566449 | -1.078397 |
| ENSDARG00000013072 | *mmp15b* | matrix metallopeptidase 15b | -2.182443 | -1.616464 | -0.606941 | -2.211012 |
| ENSDARG00000012699 | *bbx* | BBX high mobility group box domain containing | -1.768292 | -1.612774 | -0.719285 | -1.550935 |
| ENSDARG00000036999 | *DISP3* | si:dkey-32e23.6 | -1.289507 | -1.128226 | -0.546349 | -1.500739 |
| ENSDARG00000105389 | *mmel1* | membrane metallo-endopeptidase-like 1 | -2.469485 | -1.688798 | -0.830075 | -1.947533 |
| ENSDARG00000077710 | *nlgn1* | neuroligin 1 | -1.735018 | -1.005859 | -0.674276 | -1.713287 |
| ENSDARG00000059719 | *fam169aa* | family with sequence similarity 169 member Aa | -3.109918 | -3.062612 | -0.650486 | -1.509014 |
| ENSDARG00000102424 | *ARHGAP44* | si:ch211-114m9.1 | -1.611504 | -1.189785 | -0.598172 | -1.866144 |
| ENSDARG00000089297 | *si:dkeyp-115d7.2* | si:dkeyp-115d7.2 | -3.243703 | -2.413628 | -1.813715 | -3.506737 |
| ENSDARG00000043627 | *tbc1d9* | TBC1 domain family, member 9 (with GRAM domain) | -1.481471 | -1.023297 | -0.686681 | -1.37061 |
| ENSDARG00000090255 | *phlpp2* | PH domain and leucine rich repeat protein phosphatase 2 | -2.658211 | -2.468387 | -0.448758 | -1.215268 |
| ENSDARG00000079064 | *CABZ01079080.1* | XP_021322214.1 LOW QUALITY PROTEIN: cGMP-dependent 3',5'-cyclic phosphodiesterase, partial | -1.659141 | -1.084125 | -0.671684 | -1.340309 |
| ENSDARG00000068468 | *dnah3* | dynein axonemal heavy chain 3 | -1.064955 | -1.1836 | -0.479993 | -0.82103 |
| ENSDARG00000061268 | *ago2* | argonaute RISC catalytic component 2 | -1.856034 | -1.273761 | -0.454154 | -2.584963 |
| ENSDARG00000063361 | *si:ch211-1e14.1* | si:ch211-1e14.1 | -1.627273 | -1.274757 | -0.666444 | -1.326104 |
| ENSDARG00000101419 | *drp2* | dystrophin related protein 2 | -2.841302 | -1.444412 | -0.786261 | -1.667819 |
| ENSDARG00000069484 | *dab2ipa* | DAB2 interacting protein a | -1.822002 | -1.062034 | -0.59777 | -1.698619 |
| ENSDARG00000033251 | *osbpl3a* | oxysterol binding protein-like 3a | -2.438573 | -2.328949 | -0.155119 | -3.364572 |
| ENSDARG00000110874 | *ELFN2* | protein phosphatase 1 regulatory subunit 29-like | -2.018664 | -1.603626 | -0.818991 | -1.924146 |
| ENSDARG00000060865 | *scai* | suppressor of cancer cell invasion | -2.044854 | -1.28828 | -1.270414 | -1.893611 |
| ENSDARG00000091433 | *abhd17aa* | abhydrolase domain containing 17Aa | -2.504305 | -1.572394 | -1.277534 | -1.604937 |
| ENSDARG00000007561 | *cdh23* | cadherin-related 23 | -2.410284 | -2.014355 | -0.635844 | -1.613817 |
| ENSDARG00000043220 | *kcnh5a* | potassium voltage-gated channel, subfamily H (eag-related), member 5a | -1.929227 | -2.057551 | -0.83996 | -1.126264 |
| ENSDARG00000100167 | *CSDC2* | cold shock domain containing C2, RNA binding b | -1.725035 | -1.172494 | -0.858302 | -0.829125 |
| ENSDARG00000088842 | *KCNB2* | si:dkey-192j17.1 | -1.939535 | -1.074887 | -0.749416 | -1.997196 |
| ENSDARG00000090642 | *si:dkey-54n8.4* | si:dkey-54n8.4 | -1.43119 | -1.012842 | -0.548547 | -1.237882 |
| ENSDARG00000075972 | *csrnp2* | cysteine-serine-rich nuclear protein 2 | -1.854044 | -1.21874 | -0.849094 | -1.244503 |
| ENSDARG00000098702 | *zgc:165603* | zgc:165603 | -2.331206 | -2.263034 | -1 | -1.156119 |
| ENSDARG00000076787 | *angpt4* | angiopoietin 4 | -1.459432 | -1.259617 | -0.152214 | -1.975499 |
| ENSDARG00000092644 | *ago1* | argonaute RISC component 1 | -1.913412 | -1.858446 | -0.533824 | -2.156969 |
| ENSDARG00000075505 | *si:ch211-132g1.7* | si:ch211-132g1.7 | -1.039035 | -1.262442 | -0.480695 | -1.872496 |
| ENSDARG00000027564 | *pak6a* | p21 protein (Cdc42/Rac)-activated kinase 6a | -1.887973 | -1.359373 | -0.934312 | -1.871485 |
| ENSDARG00000112056 | *CABZ01066921.1* | XP_005172563.1 FERM, RhoGEF and pleckstrin domain-containing protein 1-like isoform X1 | -1.912733 | -2.219064 | -0.86948 | -1.085798 |
| ENSDARG00000099304 | *si:ch211-51f19.1* | si:ch211-51f19.1 | -1.661349 | -1.687158 | -1.02941 | -1.681959 |
| ENSDARG00000087247 | *kcnab2a* | potassium voltage-gated channel, shaker-related subfamily, beta member 2 a | -1.40408 | -1.226799 | -0.899321 | -1.428742 |
| ENSDARG00000078016 | *dtx4b* | deltex 4, E3 ubiquitin ligase b | -2.026575 | -1.01813 | -0.860105 | -1.618613 |
| ENSDARG00000011855 | *aak1a* | AP2 associated kinase 1a | -1.445 | -1.498638 | -0.526224 | -2.142152 |
| ENSDARG00000077994 | *b4galt2* | UDP-Gal:betaGlcNAc beta 1,4- galactosyltransferase, polypeptide 2 | -1.92208 | -1.110906 | -0.547005 | -1.732785 |
| ENSDARG00000009870 | *mapk8b* | mitogen-activated protein kinase 8b | -2.115477 | -1.061029 | -0.772848 | -1.900309 |
| ENSDARG00000102064 | *kcna2b* | potassium voltage-gated channel, shaker-related subfamily, member 2b | -1.422691 | -1.13179 | -0.73252 | -1.333424 |
| ENSDARG00000060103 | *cpeb3* | cytoplasmic polyadenylation element binding protein 3 | -1.750262 | -1.189246 | -0.614379 | -1.508912 |
| ENSDARG00000091061 | *slc38a3b* | solute carrier family 38 member 3b | -1.273952 | -1.297347 | -0.008019 | -1.734835 |
| MSTRG.18702 | *JCAD* | Junctional protein associated with coronary artery disease | -1.841302 | -1.178337 | -0.82338 | -1.915303 |
| ENSDARG00000075960 | *cdh24a* | cadherin 24, type 2a | -1.821887 | -1.124071 | -0.484142 | -1.502233 |
| ENSDARG00000090727 | *celf5b* | cugbp, Elav-like family member 5b | -2.427223 | -2.225589 | -0.520332 | -2.172802 |
| ENSDARG00000108787 | *TRIM67* | tripartite motif containing 67 | -2.95156 | -1.560539 | -0.904693 | -2.859301 |
| ENSDARG00000060085 | *kcnq3* | potassium voltage-gated channel, KQT-like subfamily, member 3 | -1.525527 | -1.021285 | -0.621447 | -0.854295 |
| ENSDARG00000017234 | *npy8ar* | neuropeptide Y receptor Y8a | -2.029519 | -1.589535 | -1.363614 | -2.067288 |
| ENSDARG00000079056 | *si:ch211-194c3.5* | si:ch211-194c3.5 | -1.151935 | -1.23517 | -0.44598 | 0.370135 |
| ENSDARG00000103518 | *abhd8a* | abhydrolase domain containing 8a | -2.237478 | -1.406197 | -0.532221 | -1.400784 |
| ENSDARG00000098625 | *efna5b* | ephrin-A5b | -2.400316 | -2.028014 | -1.189478 | -1.903427 |
| ENSDARG00000076041 | *wwc1* | WW and C2 domain containing 1 | -2.160647 | -1.385353 | -1.099536 | -2.066671 |
| ENSDARG00000076076 | *crtc1b* | CREB regulated transcription coactivator 1b | -1.983932 | -1.49981 | -0.85726 | -1.861448 |
| ENSDARG00000114444 | *CABZ01079081.1* | XP_021322214.1 LOW QUALITY PROTEIN: cGMP-dependent 3',5'-cyclic phosphodiesterase, partial | -1.886753 | -1.178642 | -0.644098 | -1.261876 |
| ENSDARG00000060551 | *rps6ka5* | ribosomal protein S6 kinase, polypeptide 5 | -2.019058 | -1.139019 | -0.67278 | -1.678829 |
| ENSDARG00000027777 | *tnfaip3* | tumor necrosis factor, alpha-induced protein 3 | -1.605948 | -1.198859 | -0.884923 | -1.159546 |
| ENSDARG00000012449 | *si:ch211-169p10.1* | si:ch211-169p10.1 | -2.197406 | -1.522806 | -1.064955 | -1.584963 |
| ENSDARG00000042123 | *nudt3b* | nudix (nucleoside diphosphate linked moiety X)-type motif 3b | -1.929391 | -1.257195 | -0.963513 | -2.174938 |
| ENSDARG00000079068 | *adam12* | ADAM metallopeptidase domain 12 | -1.387023 | -1.451874 | -0.387023 | -1.049328 |
| ENSDARG00000043210 | *nfic* | nuclear factor I/C | -1.973033 | -2.064955 | -0.986453 | -2.805088 |
| ENSDARG00000062579 | *kremen1* | kringle containing transmembrane protein 1 | -1.766292 | -1.619451 | -0.465123 | -2.486184 |
| ENSDARG00000015252 | *st3gal3b* | ST3 beta-galactoside alpha-2,3-sialyltransferase 3b | -1.31218 | -1.010799 | -0.625632 | -1.250375 |
| MSTRG.19005 | *Unc13a* | protein unc-13 homolog A-like isoform X2 | -4.049849 | -4.879924 | -2.119111 | -3.801921 |
| ENSDARG00000076768 | *reps2* | RALBP1 associated Eps domain containing 2 | -1.752232 | -2.191344 | -0.804321 | -1.009141 |
| ENSDARG00000026499 | *ppm1e* | protein phosphatase, Mg2+/Mn2+ dependent, 1E | -2.22784 | -1.320135 | -0.670133 | -2.411779 |
| ENSDARG00000001881 | *cacnb3a* | calcium channel, voltage-dependent, beta 3a | -2.154328 | -1.493815 | -0.775817 | -1.906891 |
| ENSDARG00000002013 | *grb10a* | growth factor receptor-bound protein 10a | -1.175969 | -1.24291 | -0.329184 | -1.742875 |
| MSTRG.17041 | *EPHA7* | ephrin type-A receptor 7-like | -1.798477 | -1.320656 | -0.623643 | -1.947533 |
| ENSDARG00000004931 | *slc8a3* | solute carrier family 8 member 3 | -2.12409 | -1.194912 | -1.163499 | -1.719892 |
| ENSDARG00000001776 | *stim2b* | stromal interaction molecule 2b | -1.750549 | -1.375039 | -0.218535 | -0.86197 |
| ENSDARG00000053389 | *jade2* | jade family PHD finger 2 | -1.683591 | -1.522768 | -0.834082 | -1.439967 |
| ENSDARG00000039901 | *sh3gl2b* | SH3 domain containing GRB2 like 2b, endophilin A1 | -2.232355 | -1.214522 | -0.68865 | -1.795944 |
| MSTRG.6364 | *CSMD1* | CUB and sushi domain-containing protein 1 isoform X1 | -1.696989 | -1.536053 | -0.599546 | -1.369695 |
| ENSDARG00000090398 | *PDE1C* | phosphodiesterase 1C, calmodulin-dependent b | -1.956057 | -1.488405 | -0.724273 | -1.97264 |
| ENSDARG00000040633 | *PRMT8* | protein arginine N-methyltransferase 8-B-like | -1.663764 | -1.026117 | -0.571129 | -1.441806 |
| ENSDARG00000062430 | *gpd2* | glycerol-3-phosphate dehydrogenase 2 (mitochondrial) | -1.256235 | -1.065853 | -0.337633 | -1.24273 |
| ENSDARG00000113609 | *TMEM164* | transmembrane protein 164 | -1.741203 | -1.929812 | -1.289507 | -1.424943 |
| ENSDARG00000102334 | *SH3RF3* | si:ch211-124n19.2 | -2.61891 | -2.104337 | -0.868269 | -1.776011 |
| ENSDARG00000018393 | *kank2* | KN motif and ankyrin repeat domains 2 | -1.762824 | -1.162595 | -0.986496 | -1.240597 |
| ENSDARG00000103609 | *mib2* | mindbomb E3 ubiquitin protein ligase 2 | -1.424777 | -1.257222 | -0.754722 | -1.00265 |
| ENSDARG00000103847 | *CR339059.1* | XP_009297443.1 adenylate cyclase type 9-like | -3.099536 | -4.141356 | -0.152003 | -1.334001 |
| ENSDARG00000008403 | *phospho1* | phosphoethanolamine/phosphocholine phosphatase 1 | -1.475883 | -1.045392 | -0.77033 | -1.250323 |
| ENSDARG00000061687 | *atxn1a* | ataxin 1a | -1.698225 | -1.045028 | -0.733592 | -1.535167 |
| ENSDARG00000115424 | *CABZ01083778.1* | XP_026100727.1 ryanodine receptor 3-like | -1.796194 | -1.675661 | -0.36257 | -1.090698 |
| ENSDARG00000003462 | *fech* | ferrochelatase | -2.133399 | -2.367864 | -0.818703 | -1.478047 |
| ENSDARG00000061014 | *kcnj5* | potassium inwardly rectifying channel subfamily J member 5 | -2.104337 | -2.118836 | -1.71477 | -1.940838 |
| ENSDARG00000071823 | *adarb2* | adenosine deaminase RNA specific B2 (inactive) | -1.857165 | -1.559084 | -0.508679 | -1.611314 |
| ENSDARG00000021909 | *VSTM2A* | zgc:110852 | -2.329602 | -1.823711 | -1.315247 | -2.034859 |
| ENSDARG00000033170 | *sult2st1* | sulfotransferase family 2, cytosolic sulfotransferase 1 | -2.398038 | -2.016254 | -1.090629 | -3.359662 |
| ENSDARG00000079102 | *PRRG3* | si:dkey-38k9.5 | -1.923197 | -1.998248 | -0.977389 | -1.225317 |
| ENSDARG00000042859 | *slc5a6a* | solute carrier family 5 member 6a | -2.462972 | -1.777608 | -0.820676 | -1.683741 |
| ENSDARG00000097869 | *si:dkey-1m11.6* | si:dkey-1m11.6 | -1.437974 | -1.098886 | -0.022937 | -1.75375 |
| ENSDARG00000039218 | *gpr101* | G protein-coupled receptor 101 | -1.78011 | -1.02268 | -0.777965 | -1.746163 |
| ENSDARG00000042114 | *bin1a* | bridging integrator 1a | -1.9841 | -1.30302 | -0.987115 | -1.990137 |
| ENSDARG00000062884 | *map3k2* | mitogen-activated protein kinase kinase kinase 2 | -2.029747 | -1.40226 | -0.815304 | -1.993221 |
| MSTRG.26783 | *Wnk3* | serine/threonine-protein kinase WNK2-like | -1.860597 | -1.297494 | -0.470865 | -1.187959 |
| ENSDARG00000101868 | *dennd1b* | DENN/MADD domain containing 1B | -2.25634 | -2.426265 | -0.801774 | -2.310788 |
| ENSDARG00000061040 | *pum2* | pumilio RNA-binding family member 2 | -1.86507 | -1.074863 | -0.737959 | -2.087463 |
| ENSDARG00000106989 | *ZNF821* | zinc finger protein 821 | -2.437686 | -1.571542 | -1.217905 | -1.061987 |
| ENSDARG00000002026 | *qkib* | QKI, KH domain containing, RNA binding b | -3.269259 | -1.852521 | -0.672999 | -2.62214 |
| ENSDARG00000093498 | *MACIR* | zgc:194281 | -1.803343 | -1.244584 | -0.84443 | -1.715697 |
| ENSDARG00000114363 | *CABZ01052743.1* | E3 ubiquitin-protein ligase RBBP6-like | 1.958605 | 2.085069 | 1.333678 | 1.24657 |
| ENSDARG00000089362 | *grn1* | granulin 1 | 3.367333 | 4.17428 | 0.15788 | 4.060659 |
| ENSDARG00000038703 | *hkdc1* | hexokinase domain containing 1 | 2.237564 | 2.037031 | 0.953079 | 1.559737 |
| ENSDARG00000045904 | *nr2e3* | nuclear receptor subfamily 2, group E, member 3 | 3.732059 | 2.824219 | 0.255097 | 2.766931 |
| ENSDARG00000023713 | *aqp1a.1* | aquaporin 1a (Colton blood group), tandem duplicate 1 | 1.261733 | 1.036994 | -0.054736 | -0.087541 |
| ENSDARG00000100803 | *zgc:113443* | zgc:113443 | 1.302788 | 1.113038 | 0.372727 | 0.509059 |
| ENSDARG00000056322 | *ldb3a* | LIM domain binding 3a | 1.60284 | 1.499746 | 1.03729 | 0.649458 |
| ENSDARG00000092362 | *hsp70.2* | heat shock cognate 70-kd protein, tandem duplicate 2 | 1.854988 | 2.312437 | 0.385167 | 2.432667 |
| ENSDARG00000032849 | *ndrg1a* | N-myc downstream regulated 1a | 1.893009 | 1.205661 | -0.328852 | 0.227739 |
| ENSDARG00000045898 | *si:ch211-152c2.3* | si:ch211-152c2.3 | 2.806762 | 1.621177 | 0.139389 | 0.607618 |
| ENSDARG00000042520 | *UTP14C* | si:dkey-251i10.3 | 1.628677 | 1.158388 | 0.32709 | 1.092018 |
| ENSDARG00000029688 | *hsp70.1* | heat shock cognate 70-kd protein, tandem duplicate 1 | 1.959358 | 2.537434 | -0.469485 | 2.740968 |
| ENSDARG00000015964 | *zgc:153311* | zgc:153311 | 5.813625 | 4.403999 | 3.085391 | 0.729911 |
| ENSDARG00000101481 | *rbp5* | retinol binding protein 1a, cellular | 1.85561 | 1.098235 | 0.152282 | 0.075508 |
| ENSDARG00000070683 | *dkk3b* | dickkopf WNT signaling pathway inhibitor 3b | 3.61629 | 2.082328 | 0.544044 | 1.384458 |
| ENSDARG00000029928 | *ap3m1* | adaptor related protein complex 3 subunit mu 1 | 1.081969 | 1.094496 | -0.197091 | 0.909552 |
| ENSDARG00000029432 | *tph1a* | tryptophan hydroxylase 1 (tryptophan 5-monooxygenase) a | 2.433426 | 1.747636 | 0.024982 | 1.345672 |
| ENSDARG00000096843 | *zgc:193593* | zgc:193593 | 5.942515 | 4.762501 | 0.716207 | 4.930737 |
| ENSDARG00000079055 | *si:dkey-85a20.4* | si:dkey-85a20.4 | 1.212202 | 1.148768 | 0.382359 | 1.156761 |
| ENSDARG00000069675 | *her8.2* | hairy-related 8.2 | 1.008027 | 1.051397 | 0.26631 | 0.149407 |
| MSTRG.20686 | *pol* | Retrovirus-related Pol polyprotein from transposon 412 | 2.09776 | 2.066328 | 0.161025 | 3.105203 |
| ENSDARG00000045808 | *rlbp1b* | retinaldehyde binding protein 1b | 2.232389 | 1.277853 | 1.07817 | 1.685963 |
| ENSDARG00000053744 | *dcdc2b* | doublecortin domain containing 2B | 1.485102 | 1.222176 | 0.777608 | 0.076726 |
| ENSDARG00000038770 | *zgc:103625* | zgc:103625 | 5.603626 | 5.098032 | 2.554589 | 4.584963 |
| ENSDARG00000089372 | *clk4a* | CDC-like kinase 4a | 1.427207 | 1.280847 | 0.140915 | 2.323948 |
| ENSDARG00000038386 | *ascl1a* | achaete-scute family bHLH transcription factor 1a | 1.080775 | 1.067877 | 0.534565 | 1.042505 |
| ENSDARG00000011235 | *otx2b* | orthodenticle homeobox 2b | 2.219937 | 1.146841 | 0.124258 | 0.929611 |
| ENSDARG00000017369 | *sema3d* | sema domain, immunoglobulin domain (Ig), short basic domain, secreted, (semaphorin) 3D | 1.967724 | 1.894067 | 0.427749 | 0.437209 |
| ENSDARG00000094012 | *si:dkey-192g7.3* | si:dkey-192g7.3 | 1.226303 | 1.401265 | 0.180169 | 0.739704 |
| ENSDARG00000099767 | *fhdc2* | FH2 domain containing 2 | 1.638074 | 1.566815 | 0.584963 | 1.217231 |
| ENSDARG00000015404 | *arl3l2* | ADP ribosylation factor like GTPase 3, like 2 | 2.595984 | 1.455403 | 0.525741 | 0.876881 |
| ENSDARG00000112944 | *LO018564.1* | XP_021336757.1 dedicator of cytokinesis protein 2 | 1.960472 | 1.602189 | 0.563136 | 1.419903 |
| ENSDARG00000002696 | *gnb3b* | guanine nucleotide binding protein (G protein), beta polypeptide 3b | 3.237039 | 2.316766 | -0.449803 | 2.532495 |
| ENSDARG00000013430 | *bhmt* | betaine-homocysteine methyltransferase | 1.218291 | 1.00463 | -0.440271 | 0.567715 |
| ENSDARG00000077641 | *thbs3a* | thrombospondin 3a | 1.346325 | 1.054862 | -0.028234 | 0.468553 |
| MSTRG.3560 | *Nim1k* | serine/threonine-protein kinase NIM1-like isoform X2 | 3.045324 | 1.478834 | 0.743092 | 1.131912 |

Table 4. Expression of Co-DEGs in the midbrain of behavior- defective mutants.

| ID | Symbol | Description | Log_2_(fc) of double KO vs WT male | Log_2_(fc) of *cyp17a1^-/-^* vs WT male | Log_2_(fc) of *tdrd12^/-^* vs WT male | Log_2_(fc) of WT female vs WT male |
| --- | --- | --- | --- | --- | --- | --- |
| MSTRG.26937 | *cyp19a1b* | cytochrome P450 19A1B | -5.940160468 | -5.61514969 | 0.284305576 | 0.239083387 |
| ENSDARG00000094857 | *dio2* | iodothyronine deiodinase 2 | -2.52986779 | -2.764006216 | 0.072151021 | -2.432068411 |
| ENSDARG00000112909 | *ZNF462* | zinc finger protein 462 | -1.192054656 | -1.510445287 | -0.294798384 | -1.678898901 |
| ENSDARG00000070717 | *slc25a18* | solute carrier family 25 member 18 | -1.303709131 | -1.039889157 | -0.38667058 | -1.655409125 |
| ENSDARG00000098631 | *CABZ01044277.1* | dystonin | -1.662641865 | -1.866733469 | -0.198196047 | -0.603993762 |
| ENSDARG00000023287 | *hsd17b3* | hydroxysteroid (17-beta) dehydrogenase 3 | -1.028693451 | -1.175373126 | 0.07144586 | -1.797401448 |
| ENSDARG00000033251 | *osbpl3a* | oxysterol binding protein-like 3a | -2.837670546 | -2.555270816 | 0.600334514 | -3.725195817 |
| MSTRG.26936 | *cyp19a1* | cytochrome P450, family 19, subfamily A, polypeptide 1b isoform X1 | -1.360474385 | -1.913243881 | -0.064130337 | -0.264369617 |
| ENSDARG00000078847 | *si:dkey-238o13.4* | si:dkey-238o13.4 | -1.632191884 | -1.672753181 | -0.017714162 | -1.734997052 |
| ENSDARG00000075230 | *tet1* | tet methylcytosine dioxygenase 1 | -1.644438979 | -1.302252045 | -0.338139419 | -1.806190049 |
| ENSDARG00000056499 | *ca6* | carbonic anhydrase VI | -1.426229325 | -1.17262017 | -0.037907385 | -2.202314271 |
| ENSDARG00000043646 | *slc6a8* | solute carrier family 6 member 8 | -1.272940882 | -1.187730493 | -0.099269592 | -1.452463186 |
| ENSDARG00000061368 | *klf13* | Kruppel-like factor 13 | -1.392261523 | -1.449737474 | 0.001787726 | -1.783625485 |
| ENSDARG00000025206 | *syt2a* | synaptotagmin IIa | -1.652939295 | -1.247705605 | -0.346729433 | -1.569422918 |
| ENSDARG00000014280 | *dlgap1a* | discs, large (Drosophila) homolog-associated protein 1a | -1.41255529 | -1.261970079 | -0.216725063 | -1.151411704 |
| ENSDARG00000035859 | *angptl4* | angiopoietin-like 4 | -1.172246313 | -1.322349874 | 0.460696216 | -1.842356933 |
| ENSDARG00000074819 | *si:dkeyp-23e4.3* | si:dkeyp-23e4.3 | -1.207580227 | -1.341845377 | 0.546815978 | -2.117605714 |
| ENSDARG00000004789 | *lrp1ba* | low density lipoprotein receptor-related protein 1Ba | -1.21480925 | -1.156445087 | -0.25507312 | -0.838335332 |
| ENSDARG00000102406 | *si:ch211-165e15.1* | si:ch211-165e15.1 | -1.08478042 | -1.763865098 | 0.262238656 | -0.184316094 |
| ENSDARG00000104207 | *pcyt1bb* | phosphate cytidylyltransferase 1, choline, beta b | -2.154328146 | -3.225881407 | 0.253419317 | 0.992137882 |
| ENSDARG00000054848 | *pdk4* | pyruvate dehydrogenase kinase, isozyme 4 | -1.534132556 | -1.697117661 | 0.221547505 | -2.035919574 |
| ENSDARG00000026499 | *ppm1e* | protein phosphatase, Mg2+/Mn2+ dependent, 1E | -1.7009501 | -1.138728743 | -0.306296123 | -2.107606225 |
| ENSDARG00000009493 | *MAP3K13* | si:ch211-45c16.2 | -1.506790887 | -1.326755857 | -0.290935382 | -1.380119083 |
| ENSDARG00000046013 | *rasl11a* | RAS-like, family 11, member A | -1.627460273 | -1.928514286 | -0.518467089 | -1.77014869 |
| ENSDARG00000075815 | *slc12a5a* | solute carrier family 12 member 5a | -1.52309167 | -1.126557492 | -0.111031312 | -1.200731048 |
| ENSDARG00000060941 | *ANKFN1* | si:ch211-277e21.2 | -1.461806426 | -1.306479696 | -0.015615613 | -2.231625701 |
| ENSDARG00000098511 | *ntrk2b* | neurotrophic tyrosine kinase, receptor, type 2b | -1.425107358 | -1.195625512 | -0.285908872 | -1.291566507 |
| ENSDARG00000019742 | *tlr4ba* | toll-like receptor 4b, duplicate a | -1.18592548 | -1.935459748 | 0.009398698 | -0.679959015 |
| ENSDARG00000076697 | *SIPA1* | zmp:0000001168 | -1.301989248 | -1.040723935 | -0.282671071 | -1.23632233 |
| ENSDARG00000094132 | *igf1* | insulin-like growth factor 1 | -1.701599286 | -1.558167182 | -0.06076234 | -1.776393793 |
| ENSDARG00000076702 | *CABZ01068356.1* | PH and SEC7 domain-containing protein 1-like | -1.318447526 | -1.466593144 | -0.488915813 | -1.216133431 |
| ENSDARG00000008912 | *slc8a2a* | solute carrier family 8 member 2a | -1.364129328 | -1.101680076 | -0.423242422 | -0.696184731 |
| ENSDARG00000073769 | *magi2b* | membrane associated guanylate kinase, WW and PDZ domain containing 2b | -1.477578965 | -1.108934372 | -0.636701471 | -1.683327631 |
| ENSDARG00000104981 | *SLC45A4* | solute carrier family 45 member 4-like | -1.989485778 | -1.347786263 | -0.2639392 | -1.732450113 |
| ENSDARG00000002197 | *pygl* | phosphorylase, glycogen, liver | -1.533114697 | -1.320420576 | -0.258666935 | -1.029715921 |
| ENSDARG00000102610 | *r3hdm2* | R3H domain containing 2 | -1.452924187 | -1.085996108 | -0.357918921 | -1.359591611 |
| ENSDARG00000036848 | *slc43a2a* | solute carrier family 43 member 2a | -1.271670009 | -1.016653071 | 0.209880909 | -2.44515283 |
| ENSDARG00000041239 | *wdr20b* | WD repeat domain 20b | -1.276517635 | -1.796908135 | -0.495527417 | -0.722361181 |
| ENSDARG00000112918 | *BX927362.2* | uncharacterized LOC101882029 | -1.083416008 | -1.416839742 | -0.513801472 | -0.30580843 |
| ENSDARG00000105389 | *mmel1* | membrane metallo-endopeptidase-like 1 | -1.78958022 | -2.526545814 | -0.896495424 | -2.163975735 |
| ENSDARG00000060025 | *nsmfa* | NMDA receptor synaptonuclear signaling and neuronal migration factor a | -1.263776342 | -1.240221614 | -0.189998416 | -1.481229714 |
| ENSDARG00000103518 | *abhd8a* | abhydrolase domain containing 8a | -1.588260091 | -1.533201073 | -0.369645596 | -1.044599998 |
| ENSDARG00000069560 | *kcnh3* | potassium voltage-gated channel, subfamily H (eag-related), member 3 | -1.31410859 | -1.064130337 | -0.415037499 | -1.890236434 |
| ENSDARG00000029668 | *crim1* | cysteine rich transmembrane BMP regulator 1 (chordin-like) | -1.276840205 | -1.017921908 | -0.395660866 | -0.81181457 |
| ENSDARG00000094491 | *si:ch211-202m22.1* | si:ch211-202m22.1 | -2.49057013 | -2.812498225 | 0.141698085 | -0.257909374 |
| ENSDARG00000053554 | *wdr76* | WD repeat domain 76 | -1.197821187 | -1.629859039 | 0.627203616 | -2.476612778 |
| ENSDARG00000087247 | *kcnab2a* | potassium voltage-gated channel, shaker-related subfamily, beta member 2 a | -1.331258083 | -1.139121802 | -0.572081209 | -1.149848209 |
| ENSDARG00000063706 | *pde8b* | phosphodiesterase 8B | -1.236339539 | -1.447236322 | -0.529698482 | -0.99755268 |
| ENSDARG00000091408 | *mtcl1* | microtubule crosslinking factor 1 | -1.316992049 | -1.206366359 | -0.128630765 | -0.897171754 |
| ENSDARG00000063332 | *shank3a* | SH3 and multiple ankyrin repeat domains 3a | -1.26101806 | -1.182591413 | 0.006710222 | -0.880647693 |
| ENSDARG00000098984 | *pid1* | phosphotyrosine interaction domain containing 1 | -1.246926607 | -1.036959532 | -0.443499855 | -1.318372468 |
| ENSDARG00000017780 | *rorcb* | RAR-related orphan receptor C b | -1.01115779 | -1.152250109 | -0.033735638 | 1.805130257 |
| ENSDARG00000076997 | *stxbp4* | syntaxin binding protein 4 | -1.327804661 | -1.030822923 | -0.516249751 | -0.842377834 |
| ENSDARG00000035732 | *arntl1b* | aryl hydrocarbon receptor nuclear translocator-like 1b | -1.124150973 | -1.028113636 | 0.710141806 | 1.048529894 |
| ENSDARG00000069431 | *slc26a4* | solute carrier family 26 member 4 | -8.26052755 | -8.26052755 | -1.03170886 | -4.523561956 |
| ENSDARG00000019963 | *sfxn1* | sideroflexin 1 | -1.386197816 | -1.080389387 | -0.547134035 | -0.959187675 |
| ENSDARG00000098906 | *herc7* | hect domain and RLD 7 | -1.887525271 | -1.868416448 | -0.203454625 | -0.27085391 |
| MSTRG.19005 | *Unc13a* | protein unc-13 homolog A-like isoform X2 | -1.824512043 | -1.75513834 | -0.789408242 | -1.950689149 |
| ENSDARG00000001881 | *cacnb3a* | calcium channel, voltage-dependent, beta 3a | -1.459926455 | -1.117457582 | -0.250031741 | -1.295455884 |
| ENSDARG00000098252 | *cpne9* | copine family member IX | -1.652995708 | -1.694022976 | -0.700975737 | -1.086123664 |
| ENSDARG00000038703 | *hkdc1* | hexokinase domain containing 1 | 2.315501826 | 2.264369617 | 1.429392792 | 1.166358386 |
| ENSDARG00000099363 | *lhb* | luteinizing hormone subunit beta | 5.788990155 | 4.680652546 | -0.540568381 | 0.459431619 |
| ENSDARG00000092362 | *hsp70.2* | heat shock cognate 70-kd protein, tandem duplicate 2 | 1.96749736 | 2.398877709 | -0.488286481 | 2.680896202 |
| ENSDARG00000079055 | *si:dkey-85a20.4* | si:dkey-85a20.4 | 1.421208402 | 1.397778735 | 0.820065974 | 0.937211047 |
| ENSDARG00000077728 | *supv3l1* | SUV3-like helicase | 1.288512683 | 1.183890141 | 0.427732572 | 1.078002512 |
| ENSDARG00000040479 | *cga* | glycoprotein hormones, alpha polypeptide | 7.414012494 | 6.016301812 | -0.874469118 | 1 |
| ENSDARG00000029688 | *hsp70.1* | heat shock cognate 70-kd protein, tandem duplicate 1 | 1.804147744 | 2.309918077 | -0.592457037 | 2.4695891 |
| ENSDARG00000057974 | *parp12b* | poly (ADP-ribose) polymerase family, member 12b | 1.160554053 | 1.070429763 | 0.112665774 | 1.699459604 |
| ENSDARG00000010841 | *fshb* | follicle stimulating hormone subunit beta | 7.030483225 | 5.618630213 | 2.341036918 | 1.584962501 |
| ENSDARG00000077471 | *hmces* | 5-hydroxymethylcytosine binding, ES cell specific | 1.435684045 | 1.425566927 | 0.858263093 | 1.10642812 |
| ENSDARG00000038185 | *gh1* | growth hormone 1 | 10.89431253 | 9.524868154 | 0 | 0 |
| ENSDARG00000100803 | *zgc:113443* | zgc:113443 | 1.356773487 | 1.005866325 | 0.103672517 | 0.825238322 |
| ENSDARG00000079415 | *dnajc30b* | DnaJ (Hsp40) homolog, subfamily C, member 30b | 1.44803307 | 1.404773009 | 0.301980686 | 1.491565964 |
| ENSDARG00000044098 | *egr2a* | early growth response 2a | 1.484996343 | 1.66219836 | -0.045398782 | 1.290556198 |

Table 5. Expression of Co-DEGs in the hindbrain of behavior- defective mutants.

| ID | Symbol | Description | Log_2_(fc) of double KO vs WT male | Log_2_(fc) of *cyp17a1^-/-^* vs WT male | Log_2_(fc) of *tdrd12^/-^* vs WT male | Log_2_(fc) of WT female vs WT male |
| --- | --- | --- | --- | --- | --- | --- |
| ENSDARG00000112909 | *ZNF462* | zinc finger protein 462 | -2.070575 | -2.162992 | -0.362967 | -1.573886 |
| ENSDARG00000079756 | *peak1* | pseudopodium-enriched atypical kinase 1 | -1.250444 | -1.47469 | -0.317355 | -1.358675 |
| ENSDARG00000027552 | *mapk1* | mitogen-activated protein kinase 1 | -1.539216 | -1.397881 | 0.111799 | -1.412878 |
| ENSDARG00000074777 | *ank1b* | ankyrin 1, erythrocytic b | -1.162618 | -1.244924 | -0.260057 | -0.92605 |
| ENSDARG00000001857 | *aff4* | AF4/FMR2 family, member 4 | -1.579505 | -1.70631 | -0.338886 | -1.692779 |
| ENSDARG00000094857 | *dio2* | iodothyronine deiodinase 2 | -1.138133 | -1.412811 | 0.163576 | -1.596213 |
| ENSDARG00000017006 | *dennd5a* | DENN/MADD domain containing 5A | -1.269507 | -1.248731 | -0.006264 | -0.940148 |
| ENSDARG00000016348 | *dip2bb* | disco-interacting protein 2 homolog Bb | -1.056116 | -1.409316 | -0.032351 | -0.525326 |
| ENSDARG00000077864 | *rasgrp3* | RAS guanyl releasing protein 3 (calcium and DAG-regulated) | -1.135878 | -1.496039 | -0.348951 | -1.030595 |
| ENSDARG00000062347 | *mtus2a* | microtubule associated tumor suppressor candidate 2a | -1.055617 | -1.15764 | -0.17585 | -0.692298 |
| ENSDARG00000075928 | *hivep3a* | HIVEP zinc finger 3a | -1.068191 | -1.148912 | -0.256671 | -0.975187 |
| ENSDARG00000017365 | *slc23a2* | solute carrier family 23 member 2 | -1.921883 | -1.955146 | -0.494567 | -1.563429 |
| ENSDARG00000014717 | *dync1h1* | dynein, cytoplasmic 1, heavy chain 1 | -1.475612 | -1.550862 | -0.247225 | -0.601143 |
| ENSDARG00000060875 | *pde1a* | phosphodiesterase 1A, calmodulin-dependent | -1.16709 | -1.446015 | -0.279014 | -0.440998 |
| ENSDARG00000077749 | *usp54b* | ubiquitin specific peptidase 54b | -1.296354 | -1.166901 | -0.34877 | -1.193923 |
| ENSDARG00000063332 | *shank3a* | SH3 and multiple ankyrin repeat domains 3a | -1.56525 | -2.525424 | -0.213529 | -1.023764 |
| ENSDARG00000054941 | *ldlrad4b* | low density lipoprotein receptor class A domain containing 4b | -1.851653 | -1.976251 | -0.005248 | -1.083167 |
| ENSDARG00000063307 | *sgsm2* | small G protein signaling modulator 2 | -1.423524 | -1.541723 | -0.017558 | -1.153885 |
| ENSDARG00000003544 | *adarb1b* | adenosine deaminase RNA specific B1b | -1.51076 | -1.825879 | -0.371168 | -0.670929 |
| ENSDARG00000061042 | *gabbr2* | gamma-aminobutyric acid (GABA) B receptor, 2 | -1.30998 | -1.545758 | -0.276444 | -0.877159 |
| ENSDARG00000103739 | *bahcc1a* | BAH domain and coiled-coil containing 1a | -1.483053 | -1.732134 | -0.272304 | -0.858063 |
| ENSDARG00000062801 | *erfl3* | Ets2 repressor factor like 3 | -1.09447 | -1.199897 | -0.001512 | -1.280216 |
| ENSDARG00000061635 | *myo5aa* | myosin VAa | -1.354568 | -1.335071 | -0.30545 | -1.076298 |
| ENSDARG00000102082 | *nr3c2* | nuclear receptor subfamily 3, group C, member 2 | -1.33534 | -1.325906 | -0.14789 | -0.691997 |
| ENSDARG00000008372 | *spred2b* | sprouty related EVH1 domain containing 2b | -1.454292 | -1.868847 | 0.015233 | -1.3416 |
| ENSDARG00000043646 | *slc6a8* | solute carrier family 6 member 8 | -1.502257 | -1.729314 | -0.236896 | -1.0299 |
| ENSDARG00000025108 | *magixa* | MAGI family member, X-linked a | -1.086816 | -1.584224 | -0.229431 | -0.795028 |
| ENSDARG00000063706 | *pde8b* | phosphodiesterase 8B | -1.735574 | -1.740798 | -0.613161 | -1.031662 |
| ENSDARG00000102406 | *si:ch211-165e15.1* | si:ch211-165e15.1 | -1.690854 | -2.367016 | -0.20304 | -0.354665 |
| ENSDARG00000104810 | *akt3a* | v-akt murine thymoma viral oncogene homolog 3a | -1.214319 | -1.332695 | -0.346526 | -0.878929 |
| ENSDARG00000063417 | *erf* | Ets2 repressor factor | -2.34645 | -2.185407 | -0.623984 | -2.342334 |
| ENSDARG00000105972 | *CABZ01050944.1* | protein shisa-9-like | -3.053901 | -2.400633 | -0.178836 | -1.863717 |
| ENSDARG00000046150 | *b4galnt4a* | beta-1,4-N-acetyl-galactosaminyl transferase 4a | -1.214451 | -1.203155 | -0.297432 | -0.853982 |
| MSTRG.15433 | *RELN* | reelin precursor | -1.310399 | -1.529229 | -0.141867 | -0.167638 |
| ENSDARG00000058248 | *si:dkeyp-77h1.4* | si:dkeyp-77h1.4 | -1.482796 | -1.597635 | -0.367201 | -0.910667 |
| ENSDARG00000009493 | *MAP3K13* | si:ch211-45c16.2 | -2.284258 | -2.608046 | 0.013675 | -1.169925 |
| ENSDARG00000025206 | *syt2a* | synaptotagmin IIa | -1.709664 | -1.569913 | -0.445705 | -1.343212 |
| ENSDARG00000076213 | *DOCK4* | si:dkey-81e3.1 | -1.435095 | -1.341986 | -0.145064 | -0.79849 |
| ENSDARG00000079805 | *tagln3a* | transgelin 3a | -1.230808 | -1.460164 | -0.153023 | -0.50543 |
| MSTRG.14233 | *UNC13C* | protein unc-13 homolog C-like isoform X2 | -1.288927 | -1.318542 | -0.33358 | -1.305591 |
| ENSDARG00000063544 | *pip4k2ab* | phosphatidylinositol-5-phosphate 4-kinase, type II, alpha b | -1.308509 | -1.116493 | -0.044256 | -0.125732 |
| ENSDARG00000075815 | *slc12a5a* | solute carrier family 12 member 5a | -1.598605 | -1.902654 | -0.429061 | -1.065983 |
| ENSDARG00000027279 | *numb* | NUMB endocytic adaptor protein | -1.266538 | -1.521685 | -0.372146 | -0.822144 |
| ENSDARG00000094577 | *cdc42se2* | CDC42 small effector 2 | -1.276806 | -1.217033 | 0.016598 | -1.42228 |
| ENSDARG00000005670 | *ttbk2a* | tau tubulin kinase 2a | -1.453946 | -1.401837 | -0.325072 | -0.99269 |
| ENSDARG00000098631 | *CABZ01044277.1* | XP_021336603.1 dystonin | -1.473432 | -1.961857 | 0.164622 | -0.180752 |
| ENSDARG00000058473 | *st6galnac3* | ST6 (alpha-N-acetyl-neuraminyl-2,3-beta-galactosyl-1,3)-N-acetylgalactosaminide alpha-2,6-sialyltransferase 3 | -1.322373 | -1.411782 | -0.341393 | -0.462936 |
| ENSDARG00000052642 | *shisa9b* | shisa family member 9b | -2.978542 | -2.967226 | -1.106139 | -1.431951 |
| ENSDARG00000013389 | *gabra4* | gamma-aminobutyric acid (GABA) A receptor, subunit alpha 4 | -1.110683 | -1.099871 | -0.355313 | -0.457883 |
| ENSDARG00000002411 | *pde4cb* | phosphodiesterase 4C, cAMP-specific b | -1.321928 | -1.401436 | -0.465148 | -0.781481 |
| ENSDARG00000088898 | *caln1* | calneuron 1 | -1.399407 | -1.787903 | -0.464973 | -1.053946 |
| ENSDARG00000078645 | *si:ch73-62l21.1* | si:ch73-62l21.1 | -1.393825 | -1.038819 | -0.005516 | -0.749483 |
| ENSDARG00000013020 | *dtnbb* | dystrobrevin, beta b | -1.341719 | -1.185355 | -0.280262 | -0.835771 |
| ENSDARG00000078425 | *oat* | ornithine aminotransferase | -1.011387 | -1.057393 | -0.18305 | -1.042658 |
| ENSDARG00000078011 | *NAV1* | neuron navigator 1a | -1.137504 | -1.276407 | -0.21832 | -0.616378 |
| ENSDARG00000020718 | *slc25a22a* | solute carrier family 25 member 22a | -1.438035 | -1.283851 | -0.497894 | -1.343903 |
| ENSDARG00000020057 | *bmpr2b* | bone morphogenetic protein receptor, type II b (serine/threonine kinase) | -1.318553 | -1.344058 | -0.24064 | -0.632708 |
| ENSDARG00000059523 | *rims4* | regulating synaptic membrane exocytosis 4 | -1.495158 | -1.466708 | -0.338157 | -0.634447 |
| ENSDARG00000076014 | *ERC1* | si:dkey-222l13.1 | -1.185529 | -1.359873 | -0.069108 | -1.119532 |
| ENSDARG00000068582 | *rnf44* | ring finger protein 44 | -1.096138 | -1.157885 | -0.353141 | -1.061562 |
| ENSDARG00000011042 | *pgbd5* | piggyBac transposable element derived 5 | -1.172591 | -1.376628 | -0.419278 | -0.451413 |
| ENSDARG00000097017 | *mical3a* | microtubule associated monooxygenase, calponin and LIM domain containing 3a | -1.045676 | -1.195783 | -0.229544 | -0.415637 |
| ENSDARG00000091408 | *mtcl1* | microtubule crosslinking factor 1 | -1.329758 | -1.555016 | -0.097411 | -0.764559 |
| ENSDARG00000075230 | *tet1* | tet methylcytosine dioxygenase 1 | -2.176189 | -2.438397 | -0.380495 | -2.428515 |
| ENSDARG00000055672 | *map3k10* | mitogen-activated protein kinase kinase kinase 10 | -1.049687 | -1.356144 | 0.037089 | -0.872436 |
| ENSDARG00000093091 | *iqsec3b* | IQ motif and Sec7 domain ArfGEF 3b | -1.092987 | -1.476997 | -0.173605 | -0.705157 |
| ENSDARG00000076697 | *SIPA1* | zmp:0000001168 | -2.138337 | -1.191257 | -0.359202 | -1.226564 |
| ENSDARG00000105099 | *golga7bb* | golgin A7 family, member Bb | -1.319398 | -1.14847 | -0.260143 | -0.683581 |
| ENSDARG00000090170 | *rab11fip4a* | RAB11 family interacting protein 4 (class II) a | -1.079754 | -1.036671 | -0.09148 | -0.924871 |
| ENSDARG00000112431 | *LO018598.1* | NP_001233205.1 reelin precursor | -1.226401 | -1.517692 | -0.042022 | -0.090775 |
| ENSDARG00000060309 | *srgap3* | SLIT-ROBO Rho GTPase activating protein 3 | -1.04171 | -1.062376 | -0.123494 | -1.017548 |
| ENSDARG00000086856 | *stk35* | serine/threonine kinase 35 | -1.333694 | -1.382387 | -0.31105 | -2.62858 |
| ENSDARG00000069101 | *napbb* | N-ethylmaleimide-sensitive factor attachment protein, beta b | -1.188646 | -1.060557 | -0.234634 | -1.012997 |
| ENSDARG00000003449 | *pde10a* | phosphodiesterase 10A | -1.343079 | -1.830312 | 0.293319 | -0.596037 |
| ENSDARG00000079912 | *rapgefl1* | Rap guanine nucleotide exchange factor (GEF)-like 1 | -1.233991 | -1.06007 | -0.122311 | -1.026845 |
| ENSDARG00000068286 | *slc7a4* | solute carrier family 7 member 4 | -1.345775 | -1.869223 | -0.454513 | -1.354912 |
| ENSDARG00000076560 | *apbb1* | amyloid beta (A4) precursor protein-binding, family B, member 1 (Fe65) | -1.108685 | -1.046116 | -0.226584 | -1.126411 |
| ENSDARG00000098857 | *lifra* | LIF receptor subunit alpha a | -1.320875 | -1.246479 | -0.128888 | -0.990547 |
| ENSDARG00000032039 | *mxd1* | MAX dimerization protein 1 | -1.357776 | -1.346818 | 0.251424 | -1.098412 |
| ENSDARG00000098320 | *grm1b* | glutamate receptor, metabotropic 1b | -1.337099 | -1.325591 | -0.21884 | -1.014709 |
| ENSDARG00000104296 | *zgc:165508* | zgc:165508 | -1.30942 | -1.115289 | -0.72236 | -0.966654 |
| ENSDARG00000058015 | *ano3* | anoctamin 3 | -1.090386 | -1.137796 | -0.081861 | -0.239283 |
| ENSDARG00000067607 | *srebf1* | sterol regulatory element binding transcription factor 1 | -1.223864 | -1.098118 | -0.442754 | -1.389287 |
| ENSDARG00000101419 | *drp2* | dystrophin related protein 2 | -1.404802 | -1.677512 | -0.218895 | -0.425346 |
| ENSDARG00000114067 | *tspan17* | tetraspanin 17 | -1.166163 | -1.186024 | -0.168984 | -1.008816 |
| ENSDARG00000075456 | *pik3ca* | phosphatidylinositol-4,5-bisphosphate 3-kinase, catalytic subunit alpha | -1.114973 | -1.13312 | -0.15234 | -0.680799 |
| ENSDARG00000018743 | *scamp5a* | secretory carrier membrane protein 5a | -1.312879 | -1.128384 | -0.310538 | -0.981028 |
| ENSDARG00000022614 | *ddx43* | DEAD (Asp-Glu-Ala-Asp) box polypeptide 43 | -5.34493 | -5.830357 | -1.652439 | 0.365385 |
| ENSDARG00000012833 | *foxn3* | forkhead box N3 | -1.281056 | -1.199361 | -0.166022 | -0.777811 |
| ENSDARG00000092119 | *si:ch211-235f12.2* | si:ch211-235f12.2 | -11.58402 | -11.58402 | -3.323495 | -11.58402 |
| ENSDARG00000042518 | *hipk2* | homeodomain interacting protein kinase 2 | -1.479513 | -1.444975 | -0.171635 | -1.074841 |
| ENSDARG00000017254 | *kcnk1b* | potassium channel, subfamily K, member 1b | -1.533894 | -1.205839 | -0.731946 | -1.498327 |
| ENSDARG00000090496 | *thsd7ab* | thrombospondin, type I, domain containing 7Ab | -1.43504 | -1.535914 | -0.101913 | -0.752291 |
| ENSDARG00000023933 | *skila* | SKI-like proto-oncogene a | -1.291058 | -1.699414 | -0.422447 | -1.003288 |
| ENSDARG00000078908 | *fbxo41* | F-box protein 41 | -1.098506 | -1.17897 | -0.0385 | -0.793357 |
| ENSDARG00000016536 | *npas2* | neuronal PAS domain protein 2 | -2.830305 | -3.400621 | 0.836915 | -0.204224 |
| ENSDARG00000059888 | *ago3a* | argonaute RISC catalytic component 3a | -2.570607 | -2.542318 | 0.216196 | -2.00965 |
| ENSDARG00000071375 | *celf5a* | cugbp, Elav-like family member 5a | -1.150172 | -1.18725 | -0.070583 | -0.576739 |
| ENSDARG00000037415 | *dlgap1b* | discs, large (Drosophila) homolog-associated protein 1b | -1.415037 | -1.782409 | -0.392842 | -0.787226 |
| ENSDARG00000017108 | *kcna1b* | potassium voltage-gated channel, shaker-related subfamily, member 1b | -1.198015 | -1.389813 | -0.832809 | -0.580763 |
| ENSDARG00000073822 | *IQSEC1* | IQ motif and Sec7 domain ArfGEF 1a | -1.420332 | -1.501368 | -0.301129 | -0.977528 |
| ENSDARG00000099547 | *nbeal1* | neurobeachin-like 1 | -1.011353 | -1.027729 | -0.215677 | -0.780149 |
| ENSDARG00000061718 | *znf704* | zinc finger protein 704 | -1.939458 | -2.321928 | -0.202493 | -0.949374 |
| ENSDARG00000105178 | *erc2* | ELKS/RAB6-interacting/CAST family member 2 | -1.344446 | -1.36732 | -0.35013 | -1.202528 |
| ENSDARG00000079348 | *GRIN2B* | glutamate receptor ionotropic, NMDA 2B-like | -1.155463 | -1.192066 | -0.302788 | -0.671327 |
| ENSDARG00000003069 | *btbd7* | BTB (POZ) domain containing 7 | -1.166474 | -1.33931 | -0.455334 | -0.661238 |
| ENSDARG00000053122 | *clvs2* | clavesin 2 | -1.213458 | -1.088059 | 0.186805 | -1.037582 |
| ENSDARG00000077686 | *aak1b* | AP2 associated kinase 1b | -1.555519 | -1.483049 | -0.585334 | -1.073218 |
| ENSDARG00000045301 | *st8sia3* | ST8 alpha-N-acetyl-neuraminide alpha-2,8-sialyltransferase 3 | -1.844911 | -2.32812 | -0.735422 | -1.301225 |
| ENSDARG00000078839 | *LRRTM4* | si:dkey-28o19.1 | -1.683194 | -1.824809 | -0.565013 | 0.281182 |
| ENSDARG00000076520 | *mafb* | v-maf avian musculoaponeurotic fibrosarcoma oncogene homolog b (paralog b) | -1.281696 | -1.01503 | -0.436124 | -1.014167 |
| ENSDARG00000076622 | *atp13a3* | ATPase 13A3 | -1.560545 | -1.408542 | 0.847903 | -0.073152 |
| ENSDARG00000005834 | *gatad2b* | GATA zinc finger domain containing 2B | -1.262565 | -1.148823 | -0.075708 | -1.074718 |
| ENSDARG00000053389 | *jade2* | jade family PHD finger 2 | -1.457548 | -1.47142 | -0.632226 | -0.842979 |
| ENSDARG00000053326 | *gna11b* | guanine nucleotide binding protein (G protein), alpha 11b (Gq class) | -1.424054 | -1.272504 | -0.288196 | -0.746171 |
| ENSDARG00000090656 | *tomm20a* | translocase of outer mitochondrial membrane 20 | -1.171543 | -1.06619 | -0.061682 | -0.806546 |
| ENSDARG00000074258 | *gramd1bb* | GRAM domain containing 1Bb | -1.100532 | -1.013476 | -0.513271 | -0.773551 |
| ENSDARG00000076041 | *wwc1* | WW and C2 domain containing 1 | -1.491659 | -1.869296 | -0.427528 | -1.192645 |
| ENSDARG00000061363 | *GFOD1* | si:ch211-276f18.2 | -1.010454 | -1.561714 | -0.489251 | -0.823723 |
| ENSDARG00000004789 | *lrp1ba* | low density lipoprotein receptor-related protein 1Ba | -1.097611 | -1.241621 | -0.101222 | -1.282035 |
| ENSDARG00000098906 | *herc7* | hect domain and RLD 7 | -2.586146 | -2.867778 | -0.702661 | -0.323704 |
| MSTRG.19521 | *C14orf132* | chromosome 14 open reading frame 132, partial | -1.22281 | -1.484676 | -0.178238 | -1.14051 |
| ENSDARG00000005394 | *dnmt3aa* | DNA (cytosine-5-)-methyltransferase 3 alpha a | -1.329582 | -1.215286 | 0.014431 | -1.194173 |
| ENSDARG00000077582 | *ank3b* | ankyrin 3b | -1.30348 | -1.378374 | -0.235517 | -0.424697 |
| ENSDARG00000099841 | *prkcab* | protein kinase C, alpha, b | -1.36087 | -1.200552 | -0.353938 | -0.56668 |
| ENSDARG00000037069 | *zdhhc14* | zinc finger DHHC-type containing 14 | -1.271016 | -1.467955 | 0.208108 | -1.351244 |
| ENSDARG00000100908 | *lcor* | ligand dependent nuclear receptor corepressor | -1.896582 | -2.297681 | -0.643504 | -1.316296 |
| MSTRG.21108 | *SHC3* | SHC-transforming protein 3 | -1.483395 | -1.143855 | -0.12287 | -1.070449 |
| ENSDARG00000091130 | *kcnq2b* | potassium voltage-gated channel, KQT-like subfamily, member 2b | -1.027676 | -1.146261 | -0.24267 | -0.793211 |
| ENSDARG00000069484 | *dab2ipa* | DAB2 interacting protein a | -1.34504 | -1.390555 | -0.06986 | -1.360053 |
| ENSDARG00000063079 | *ago3b* | argonaute RISC catalytic component 3b | -1.855052 | -1.813231 | -0.965235 | -1.011777 |
| ENSDARG00000062550 | *rc3h1a* | ring finger and CCCH-type domains 1a | -1.108892 | -1.047877 | -0.323802 | -0.633392 |
| ENSDARG00000068830 | *zgc:172139* | zgc:172139 | -1.831912 | -1.399182 | -0.424635 | -1.234619 |
| ENSDARG00000040633 | *PRMT8* | protein arginine N-methyltransferase 8-B-like | -1.448308 | -1.1651 | 0.187302 | -1.358386 |
| ENSDARG00000090814 | *si:dkey-18a10.3* | si:dkey-18a10.3 | -1.692726 | -1.325216 | 0.097896 | -7.005095 |
| ENSDARG00000001881 | *cacnb3a* | calcium channel, voltage-dependent, beta 3a | -1.119299 | -1.79595 | -0.19031 | -0.619728 |
| ENSDARG00000100815 | *srsf3a* | serine and arginine rich splicing factor 3a | -1.156194 | -1.299762 | -0.289734 | -2.042971 |
| ENSDARG00000018399 | *rps6kb1a* | ribosomal protein S6 kinase b, polypeptide 1a | -1.100521 | -1.158337 | 0.100952 | -1.39318 |
| ENSDARG00000021996 | *ppp2r2aa* | protein phosphatase 2, regulatory subunit B, alpha a | -1.048103 | -1.207051 | -0.498693 | -0.714611 |
| ENSDARG00000019990 | *cabp1a* | calcium binding protein 1a | -1.083325 | -1.258324 | -0.468064 | -0.758158 |
| ENSDARG00000017220 | *otud7b* | OTU deubiquitinase 7B | -1.632738 | -1.575594 | -0.337282 | -0.659705 |
| ENSDARG00000005126 | *large1* | LARGE xylosyl- and glucuronyltransferase 1 | -1.285737 | -1.656425 | -0.144672 | -0.620251 |
| ENSDARG00000057633 | *cxcr4a* | chemokine (C-X-C motif) receptor 4a | -1.090581 | -2.058103 | -0.117357 | -2.192645 |
| ENSDARG00000031336 | *hsd20b2* | hydroxysteroid (20-beta) dehydrogenase 2 | -1.018408 | -1.268073 | 0.183327 | -2.025372 |
| ENSDARG00000054848 | *pdk4* | pyruvate dehydrogenase kinase, isozyme 4 | -1.079886 | -1.485825 | 0.70289 | -2.401814 |
| MSTRG.37189 | *GPR22* | probable G-protein coupled receptor 22 | -1.246858 | -1.101984 | -0.779443 | -1.530356 |
| ENSDARG00000071055 | *ZNF512B* | si:dkey-256k13.2 | -1.245422 | -1.257362 | 0.037946 | -0.442604 |
| ENSDARG00000074120 | *usp2b* | ubiquitin specific peptidase 2b | -1.084648 | -1.154762 | -0.199495 | -2.19875 |
| ENSDARG00000027423 | *igf1ra* | insulin-like growth factor 1a receptor | -1.029595 | -1.052196 | -0.118226 | -0.550589 |
| ENSDARG00000070076 | *actr2b* | actin related protein 2b | -1.056916 | -1.079322 | 0.021606 | -1.267554 |
| ENSDARG00000101949 | *numbl* | NUMB like endocytic adaptor protein | -1.194816 | -1.261517 | -0.427749 | -0.722798 |
| ENSDARG00000102610 | *r3hdm2* | R3H domain containing 2 | -1.215619 | -1.418436 | -0.128929 | -1.077877 |
| ENSDARG00000061699 | *sipa1l3* | signal-induced proliferation-associated 1 like 3 | -1.08573 | -1.096813 | 0.149747 | -0.836254 |
| ENSDARG00000074376 | *mdga1* | MAM domain containing glycosylphosphatidylinositol anchor 1 | -1.439984 | -1.497015 | -0.428843 | -1.624663 |
| ENSDARG00000018716 | *dgkh* | diacylglycerol kinase, eta | -1.340624 | -1.07569 | -0.38037 | -0.787371 |
| ENSDARG00000062305 | *rims3* | regulating synaptic membrane exocytosis 3 | -1.569532 | -1.140609 | -0.250957 | -1.351132 |
| ENSDARG00000098511 | *ntrk2b* | neurotrophic tyrosine kinase, receptor, type 2b | -1.412149 | -1.540831 | -0.219745 | -1.221429 |
| ENSDARG00000104727 | *ino80da* | INO80 complex subunit Da | -1.900101 | -2.142958 | -0.40239 | -0.951816 |
| ENSDARG00000040700 | *nab1b* | NGFI-A binding protein 1b (EGR1 binding protein 1) | -1.832997 | -1.171425 | -0.879523 | -1.804787 |
| ENSDARG00000043835 | *rab3ab* | RAB3A, member RAS oncogene family, b | -1.338188 | -1.031519 | -0.260398 | -0.750173 |
| ENSDARG00000005232 | *ccdc85ca* | coiled-coil domain containing 85C, a | -1.323634 | -1.234666 | -0.075258 | -1.787876 |
| ENSDARG00000043226 | *nfixa* | nuclear factor I/Xa | -1.756637 | -1.57898 | -0.391693 | -0.853202 |
| ENSDARG00000098801 | *sbno2a* | strawberry notch homolog 2a | -1.671377 | -1.796908 | -0.18786 | -0.942449 |
| ENSDARG00000110973 | *tln2b* | talin 2b | -1.043991 | -1.042091 | -0.24235 | -0.682689 |
| ENSDARG00000062462 | *shisa7a* | shisa family member 7a | -1.034727 | -1.022002 | 0.214797 | -1.648738 |
| ENSDARG00000018967 | *gabbr1a* | gamma-aminobutyric acid (GABA) B receptor, 1a | -1.574041 | -1.762031 | -0.172119 | -1.167187 |
| ENSDARG00000060901 | *trim62.1* | tripartite motif containing 62, tandem duplicate 1 | -1.087384 | -1.098101 | -0.234059 | -0.92713 |
| ENSDARG00000035732 | *arntl1b* | aryl hydrocarbon receptor nuclear translocator-like 1b | -1.047532 | -1.240931 | 0.63919 | 1.034857 |
| ENSDARG00000068787 | *slc6a17* | solute carrier family 6 member 17 | -1.45957 | -1.491028 | -0.886555 | -1.289137 |
| ENSDARG00000061687 | *atxn1a* | ataxin 1a | -1.253757 | -1.262783 | 0.062763 | -0.8238 |
| ENSDARG00000076757 | *ephb1* | EPH receptor B1 | -1.876039 | -2.002355 | -0.115085 | -0.38016 |
| ENSDARG00000060539 | *shank1* | SH3 and multiple ankyrin repeat domains 1 | -1.766112 | -1.494109 | -0.112475 | -1.177882 |
| ENSDARG00000089079 | *si:ch211-214b16.3* | si:ch211-214b16.3 | -1.773229 | -2.577309 | -0.594587 | -0.861102 |
| ENSDARG00000079581 | *nyap2a* | neuronal tyrosine-phosphorylated phosphoinositide-3-kinase adaptor 2a | -1.525256 | -1.505891 | 0.192081 | -0.286249 |
| ENSDARG00000061992 | *dot1l* | DOT1-like histone H3K79 methyltransferase | -1.272048 | -1.190033 | -0.186241 | -0.136007 |
| ENSDARG00000068421 | *ttc9b* | tetratricopeptide repeat domain 9B | -1.406038 | -1.269198 | -0.42625 | -1.112115 |
| ENSDARG00000088307 | *mrtfba* | myocardin related transcription factor Ba | -1.439506 | -1.375156 | -0.212782 | -0.714741 |
| ENSDARG00000099487 | *si:ch73-233f7.7* | si:ch73-233f7.7 | -1.025502 | -1.122046 | -0.702431 | -0.917226 |
| ENSDARG00000031588 | *si:dkey-239b22.1* | si:dkey-239b22.1 | -1.028014 | -1.490986 | -0.746243 | -0.656046 |
| ENSDARG00000060865 | *scai* | suppressor of cancer cell invasion | -1.253928 | -1.140291 | -0.36863 | -0.798608 |
| ENSDARG00000087260 | *mtss1lb* | MTSS I-BAR domain containing 2b | -1.931287 | -1.718513 | -0.162795 | -1.084534 |
| ENSDARG00000102067 | *grm5b* | glutamate receptor, metabotropic 5b | -1.974005 | -2.3484 | -0.103288 | -0.579726 |
| ENSDARG00000017785 | *zgc:158689* | zgc:158689 | -1.224197 | -1.685803 | 0.903866 | 0.358129 |
| ENSDARG00000032072 | *cdk16* | cyclin-dependent kinase 16 | -1.022403 | -1.060145 | -0.28747 | -0.211504 |
| ENSDARG00000103333 | *baiap2b* | BAR/IMD domain containing adaptor protein 2b | -2.324265 | -1.97854 | -0.496574 | -2.922717 |
| ENSDARG00000011855 | *aak1a* | AP2 associated kinase 1a | -1.505569 | -1.230077 | -0.502309 | -1.413888 |
| ENSDARG00000098566 | *si:ch73-233f7.4* | si:ch73-233f7.4 | -1.529891 | -1.398294 | -0.523825 | -1.119928 |
| ENSDARG00000089536 | *erbb4b* | erb-b2 receptor tyrosine kinase 4b | -1.233888 | -1.354182 | -0.438121 | -1.27684 |
| ENSDARG00000074742 | *elmod3* | ELMO/CED-12 domain containing 3 | -1.740702 | -1.360572 | -0.629368 | -1.281908 |
| ENSDARG00000075539 | *snphb* | syntaphilin b | -1.192104 | -1.448234 | -0.369076 | -0.858543 |
| ENSDARG00000060597 | *zgc:158659* | zgc:158659 | -1.142801 | -1.392553 | -0.508651 | -1.053867 |
| ENSDARG00000026988 | *tbc1d22b* | TBC1 domain family, member 22B | -1.459136 | -2.045727 | -1.133954 | -0.576106 |
| ENSDARG00000092644 | *ago1* | argonaute RISC component 1 | -1.508341 | -1.703357 | -0.392864 | -0.68919 |
| ENSDARG00000028469 | *rps6ka2* | ribosomal protein S6 kinase, polypeptide 2 | -1.242722 | -1.138311 | -0.393664 | -0.906218 |
| ENSDARG00000061368 | *klf13* | Kruppel-like factor 13 | -2.284939 | -2.311394 | -0.112204 | -2.416398 |
| ENSDARG00000054036 | *rnf34b* | ring finger protein 34b | -1.399985 | -1.884371 | -0.176106 | -0.982346 |
| ENSDARG00000098816 | *kcnc3b* | potassium voltage-gated channel, Shaw-related subfamily, member 3b | -1.135201 | -1.24017 | -0.540149 | -1.245625 |
| ENSDARG00000055565 | *cacnb2b* | calcium channel, voltage-dependent, beta 2b | -1.245756 | -1.106173 | -0.293877 | -1.069231 |
| ENSDARG00000102424 | *ARHGAP44* | si:ch211-114m9.1 | -1.09148 | -1.192645 | -0.61118 | -0.715225 |
| ENSDARG00000005586 | *zbtb20* | zinc finger and BTB domain containing 20 | -2.776494 | -2.887525 | -0.361456 | -1.055648 |
| ENSDARG00000104283 | *cdc42bpaa* | CDC42 binding protein kinase alpha (DMPK-like) a | -1.868562 | -1.744383 | -0.437405 | -0.868562 |
| ENSDARG00000074905 | *camk1da* | calcium/calmodulin-dependent protein kinase 1Da | -1.154051 | -1.187218 | -0.345135 | -0.797566 |
| ENSDARG00000102198 | *si:ch73-233f7.3* | si:ch73-233f7.3 | -1.499571 | -1.385566 | -0.707053 | -0.821978 |
| ENSDARG00000041239 | *wdr20b* | WD repeat domain 20b | -1.204358 | -1.889201 | 0.05142 | -0.057673 |
| ENSDARG00000026499 | *ppm1e* | protein phosphatase, Mg2+/Mn2+ dependent, 1E | -1.478134 | -1.170534 | -0.223611 | -1.223611 |
| ENSDARG00000104981 | *SLC45A4* | solute carrier family 45 member 4-like | -2.310183 | -2.290818 | -0.374978 | -1.390353 |
| ENSDARG00000036560 | *gpr185b* | G protein-coupled receptor 185 b | -1.079767 | -1.018936 | -0.592921 | -0.822128 |
| ENSDARG00000039901 | *sh3gl2b* | SH3 domain containing GRB2 like 2b, endophilin A1 | -1.350195 | -1.431523 | -0.534049 | -1.429484 |
| ENSDARG00000038121 | *si:ch211-13k12.2* | si:ch211-13k12.2 | -1.519778 | -1.745071 | -0.850424 | -1.59561 |
| ENSDARG00000061231 | *tinagl1* | tubulointerstitial nephritis antigen-like 1 | -1.468031 | -1.576708 | -0.353309 | -2.464763 |
| ENSDARG00000060641 | *prkaa2* | protein kinase, AMP-activated, alpha 2 catalytic subunit | -1.686501 | -1.931613 | -0.496676 | -0.716874 |
| ENSDARG00000103519 | *adamts1* | ADAM metallopeptidase with thrombospondin type 1 motif, 1 | -1.044139 | -1.12479 | -0.096918 | -0.811777 |
| ENSDARG00000100958 | *HECW1* | si:ch211-188f17.1 | -1.104469 | -1.192645 | -0.491273 | -1.026767 |
| ENSDARG00000069608 | *palm2akap2* | palm2 and akap2 fusion | -1.725335 | -1.823367 | -0.720079 | -2.016012 |
| ENSDARG00000090815 | *kcnj10a* | potassium inwardly rectifying channel subfamily J member 10a | -1.816119 | -1.775667 | -0.501045 | -1.900594 |
| ENSDARG00000090421 | *SH2B2* | SH2B adaptor protein 2 | -1.094408 | -1.228397 | -0.062769 | -0.462243 |
| ENSDARG00000070522 | *cacna1ia* | calcium voltage-gated channel subunit alpha1 Ia | -1.110807 | -1.277704 | -0.166421 | -0.104758 |
| ENSDARG00000076068 | *crtc1a* | CREB regulated transcription coactivator 1a | -1.237134 | -1.288322 | -0.488898 | -0.713163 |
| ENSDARG00000043553 | *ches1* | checkpoint suppressor 1 | -1.280956 | -1.380492 | -0.01392 | -1.079322 |
| ENSDARG00000062248 | *ptpn21* | protein tyrosine phosphatase non-receptor type 21 | -1.886132 | -2.169925 | -0.371559 | -1.154328 |
| ENSDARG00000075271 | *rapgef5a* | Rap guanine nucleotide exchange factor (GEF) 5a | -1.09287 | -1.304643 | -0.029073 | -0.126512 |
| ENSDARG00000061471 | *sema5ba* | sema domain, seven thrombospondin repeats (type 1 and type 1-like), transmembrane domain (TM) and short cytoplasmic domain, (semaphorin) 5Ba | -1.158531 | -1.062831 | -0.25079 | -1.29923 |
| ENSDARG00000052368 | *cltb* | clathrin, light chain B | -1.492914 | -1.612378 | -0.462192 | -0.949959 |
| ENSDARG00000053771 | *apbb3* | amyloid beta (A4) precursor protein-binding, family B, member 3 | -1.246747 | -1.639531 | -0.193545 | -1.104494 |
| ENSDARG00000104005 | *slc4a3* | solute carrier family 4 member 3 | -1.059906 | -1.142368 | 0.002022 | -0.638554 |
| ENSDARG00000018393 | *kank2* | KN motif and ankyrin repeat domains 2 | -1.117437 | -1.028475 | -0.524003 | -0.527385 |
| ENSDARG00000099265 | *slc7a5* | solute carrier family 7 member 5 | -1.731912 | -1.238341 | -0.261213 | -1.876833 |
| ENSDARG00000087247 | *kcnab2a* | potassium voltage-gated channel, shaker-related subfamily, beta member 2 a | -1.381487 | -1.252614 | -0.19603 | -0.645095 |
| ENSDARG00000054253 | *sobpa* | sine oculis binding protein homolog (Drosophila) a | -1.267366 | -1.536362 | -0.682403 | -1.072863 |
| ENSDARG00000002197 | *pygl* | phosphorylase, glycogen, liver | -1.227568 | -1.125413 | 0.133153 | -0.842325 |
| ENSDARG00000101933 | *CELF6* | si:dkey-205h23.2 | -1.153351 | -1.213931 | -0.730917 | -0.223808 |
| ENSDARG00000036626 | *sema6ba* | sema domain, transmembrane domain (TM), and cytoplasmic domain, (semaphorin) 6Ba | -1.570165 | -1.775484 | -0.709142 | -1.396973 |
| ENSDARG00000042259 | *tgfbr1b* | transforming growth factor, beta receptor 1 b | -1.402098 | -1.674864 | -0.785164 | -1.496016 |
| ENSDARG00000008727 | *ranbp3a* | RAN binding protein 3a | -1.364093 | -1.628225 | -0.709525 | -0.688767 |
| ENSDARG00000041926 | *dlg4a* | discs, large homolog 4a (Drosophila) | -1.017237 | -1.186295 | -0.134594 | -0.280965 |
| ENSDARG00000099199 | *itchb* | itchy E3 ubiquitin protein ligase b | -1.075884 | -1.11667 | -0.158642 | -1.085973 |
| MSTRG.26783 | *Wnk3* | serine/threonine-protein kinase WNK2-like | -1.111411 | -1.353486 | -0.411874 | -0.327708 |
| ENSDARG00000059850 | *slc35f3b* | solute carrier family 35 member F3b | -1.329758 | -1.80151 | -0.846436 | -0.846436 |
| ENSDARG00000078135 | *mrc2* | mannose receptor, C type 2 | -1.056413 | -1.255394 | -0.021437 | -0.069751 |
| ENSDARG00000075505 | *si:ch211-132g1.7* | si:ch211-132g1.7 | -1.641231 | -1.181799 | -0.596837 | -1.414162 |
| ENSDARG00000060085 | *kcnq3* | potassium voltage-gated channel, KQT-like subfamily, member 3 | -1.147161 | -1.228749 | -0.450923 | -1.069942 |
| ENSDARG00000098215 | *BICD2* | zgc:171572 | -1.250407 | -1.26939 | 0.046794 | -0.365688 |
| ENSDARG00000090727 | *celf5b* | cugbp, Elav-like family member 5b | -2.246161 | -2.221214 | -0.579668 | -1.160673 |
| ENSDARG00000074644 | *spock1* | SPARC (osteonectin), cwcv and kazal like domains proteoglycan 1 | -2.715631 | -2.396353 | 0.141893 | -2.483816 |
| ENSDARG00000079933 | *SLC46A3* | si:dkey-5g14.1 | -1.296075 | -1.551332 | 0.301827 | -0.310068 |
| ENSDARG00000101547 | *CR354395.2* | XP_030634258.1 receptor-type tyrosine-protein phosphatase gamma-like | -1.597563 | -1.636037 | -0.293974 | -0.541709 |
| ENSDARG00000086345 | *cux2b* | cut-like homeobox 2b | -2.137155 | -2.240532 | -0.310201 | -0.663838 |
| ENSDARG00000035273 | *tmtc2b* | transmembrane O-mannosyltransferase targeting cadherins 2b | -1.316474 | -1.062034 | -0.257217 | -0.429684 |
| ENSDARG00000078485 | *snpha* | syntaphilin a | -2.604402 | -1.906546 | -0.69975 | -1.472246 |
| ENSDARG00000078567 | *lonrf1l* | LON peptidase N-terminal domain and ring finger 1, like | -1.043427 | -1.43266 | 0.023009 | -2.147135 |
| ENSDARG00000069500 | *hs6st3b* | heparan sulfate 6-O-sulfotransferase 3b | -1.368029 | -1.357881 | -0.287171 | -0.589573 |
| MSTRG.4700 | *ADAP1* | PREDICTED: arf-GAP with dual PH domain-containing protein 1-like | -1.266551 | -1.23078 | -0.534423 | -0.233727 |
| ENSDARG00000077762 | *si:rp71-79p20.2* | si:rp71-79p20.2 | -1.204106 | -1.082206 | -0.600299 | -0.775741 |
| ENSDARG00000025468 | *bnip3la* | BCL2 interacting protein 3 like a | -1.437299 | -1.330877 | -0.518949 | -0.300214 |
| ENSDARG00000104483 | *gipc1* | GIPC PDZ domain containing family, member 1 | -1.270161 | -1.061347 | -0.280546 | -0.830211 |
| ENSDARG00000071904 | *pcdh2ab1* | protocadherin 2 alpha b 1 | -1.847125 | -1.550508 | -1.12276 | -0.589328 |
| ENSDARG00000073769 | *magi2b* | membrane associated guanylate kinase, WW and PDZ domain containing 2b | -2.173829 | -1.465292 | -0.789914 | -1.245979 |
| ENSDARG00000086931 | *CABZ01029822.1* | uncharacterized LOC100004582 | -1.569908 | -1.459432 | -0.326981 | -1.023735 |
| ENSDARG00000009733 | *kif21b* | kinesin family member 21B | -1.85561 | -1.21818 | -0.263034 | -0.821663 |
| ENSDARG00000099133 | *UNC5C* | si:ch73-72b7.1 | -1.214809 | -1.339137 | -0.349998 | -0.955088 |
| ENSDARG00000102142 | *ece2b* | endothelin converting enzyme 2b | -1.257616 | -1.20854 | -0.14022 | -1.14714 |
| ENSDARG00000057107 | *si:ch73-383g2.1* | si:ch73-383g2.1 | -1.696908 | -1.430351 | -0.44065 | -0.445087 |
| ENSDARG00000042859 | *slc5a6a* | solute carrier family 5 member 6a | -1.667017 | -1.433653 | -0.793549 | -0.938888 |
| ENSDARG00000089800 | *prr7* | proline rich 7 (synaptic) | -1.510308 | -1.252607 | -1.157829 | -1.570089 |
| ENSDARG00000069560 | *kcnh3* | potassium voltage-gated channel, subfamily H (eag-related), member 3 | -1.099536 | -1.078551 | -0.603578 | -0.925506 |
| ENSDARG00000037815 | *b4galt5* | UDP-Gal:betaGlcNAc beta 1,4- galactosyltransferase, polypeptide 5 | -1.838249 | -2.163825 | -0.609625 | -0.936224 |
| ENSDARG00000025920 | *tiam1b* | TIAM Rac1 associated GEF 1b | -1.389042 | -1.436592 | -0.902363 | -1.426055 |
| ENSDARG00000052978 | *mbnl1* | muscleblind-like splicing regulator 1 | -1.185692 | -1.023893 | -0.095062 | -1.093574 |
| ENSDARG00000003462 | *fech* | ferrochelatase | -1.900209 | -2.236493 | 0.081824 | -1.547193 |
| ENSDARG00000093498 | *MACIR* | zgc:194281 | -1.528255 | -1.332139 | -0.839028 | -0.839028 |
| ENSDARG00000031618 | *unm_sa1261* | un-named sa1261 | -1.423694 | -1.500163 | 0.056883 | -2.740609 |
| ENSDARG00000101322 | *tfr1a* | transferrin receptor 1a | -1.06517 | -1.024718 | -0.30447 | -1.024718 |
| ENSDARG00000019588 | *chico* | chico | -1.237715 | -1.193772 | -0.271574 | -1.133724 |
| ENSDARG00000100097 | *mafgb* | v-maf avian musculoaponeurotic fibrosarcoma oncogene homolog Gb | -1.270961 | -1.156402 | -0.759372 | -0.496338 |
| MSTRG.4521 | *TMEM63B* | CSC1-like protein 2 isoform X3 | -1.322697 | -1.201019 | 0.255839 | -0.540289 |
| ENSDARG00000098654 | *FQ976914.1* | raftlin-like | -3.834836 | -2.125884 | -0.82103 | -1.793808 |
| ENSDARG00000077207 | *lzts2b* | leucine zipper, putative tumor suppressor 2b | -2.537134 | -1.738768 | 0.01612 | -2.812768 |
| ENSDARG00000015057 | *ube2d4* | ubiquitin-conjugating enzyme E2D 4 (putative) | -1.453718 | -1.119299 | -0.135215 | -1.027321 |
| ENSDARG00000034605 | *zgc:153169* | zgc:153169 | -1.228659 | -1.031027 | -0.74152 | -0.013707 |
| ENSDARG00000094491 | *si:ch211-202m22.1* | si:ch211-202m22.1 | -3.899473 | -3.076351 | 0.365871 | -0.410088 |
| ENSDARG00000102380 | *cnnm4a* | cyclin and CBS domain divalent metal cation transport mediator 4a | -1.480432 | -1.239871 | -0.769292 | -1.259703 |
| ENSDARG00000109726 | *PLCB1* | si:ch1073-140o9.2 | -1.666757 | -1.260764 | -0.177042 | -0.24273 |
| ENSDARG00000060944 | *rnf217* | ring finger protein 217 | -1.549339 | -1.514573 | -0.590986 | -0.60921 |
| ENSDARG00000061723 | *tmem64* | transmembrane protein 64 | -1.192645 | -1.085117 | -0.390206 | -1.085117 |
| ENSDARG00000025797 | *abhd2a* | abhydrolase domain containing 2a | -1.485427 | -1.117523 | -0.163499 | -1.418313 |
| ENSDARG00000070536 | *creb5b* | cAMP responsive element binding protein 5b | -1.260867 | -1.038474 | -0.251151 | -1.620654 |
| ENSDARG00000078775 | *RASA2* | RAS p21 protein activator 2 | -1.369908 | -1.186878 | 0.141176 | -1.019001 |
| ENSDARG00000109769 | *LO017835.1* | tumor protein p53 inducible nuclear protein 2 | -1.61471 | -1.536368 | -0.624403 | -1.244645 |
| ENSDARG00000037691 | *pcdh2ab2* | protocadherin 2 alpha b2 | -1.608018 | -2.079567 | 0.056922 | -0.563136 |
| ENSDARG00000060246 | *slc16a6b* | solute carrier family 16 member 6b | -1.053328 | -1.216184 | 0.503009 | -2.655397 |
| ENSDARG00000077728 | *supv3l1* | SUV3-like helicase | 1.478646 | 1.286071 | 0.629565 | 1.449791 |
| ENSDARG00000111102 | *camk2n2* | calcium/calmodulin-dependent protein kinase II inhibitor 2 | 1.249927 | 1.226137 | 0.385318 | 0.790448 |
| ENSDARG00000010770 | *sox19a* | SRY-box transcription factor 19a | 1.33631 | 1.517381 | 0.16665 | 1.022574 |
| ENSDARG00000100803 | *zgc:113443* | zgc:113443 | 1.709291 | 1.446256 | 0.24894 | 0.679574 |
| ENSDARG00000030463 | *tppp3* | tubulin polymerization-promoting protein family member 3 | 1.57301 | 1.736251 | 0.627191 | 0.230532 |
| ENSDARG00000095580 | *si:ch211-67e16.11* | si:ch211-67e16.11 | 1.015689 | 1.130184 | 0.061759 | 0.170244 |
| ENSDARG00000060264 | *si:dkey-37g12.1* | si:dkey-37g12.1 | 1.119111 | 1.00197 | 0.600644 | 0.301284 |
| ENSDARG00000054906 | *ier5l* | immediate early response 5-like | 1.037846 | 1.078155 | 0.223426 | 1.247394 |
| ENSDARG00000073944 | *si:ch73-386h18.1* | si:ch73-386h18.1 | 1.30648 | 1.086961 | 0.517984 | -0.052151 |
| ENSDARG00000036344 | *calb2b* | calbindin 2b | 1.571885 | 1.227516 | 0.328319 | 0.355436 |
| ENSDARG00000079055 | *si:dkey-85a20.4* | si:dkey-85a20.4 | 1.532341 | 1.385515 | 0.596584 | 0.681662 |
| ENSDARG00000031136 | *moxd1* | monooxygenase, DBH-like 1 | 1.497212 | 1.403777 | 0.286994 | 0.554736 |
| ENSDARG00000094557 | *nupr1b* | nuclear protein 1b | 1.306851 | 1.551851 | 0.566996 | 0.779541 |
| ENSDARG00000077504 | *si:ch211-103n10.5* | si:ch211-103n10.5 | 1.232057 | 1.072093 | 0.001394 | -0.38149 |
| ENSDARG00000057974 | *parp12b* | poly (ADP-ribose) polymerase family, member 12b | 1.108524 | 1.110883 | -0.027159 | 1.490301 |
| ENSDARG00000022531 | *ntn1b* | netrin 1b | 1.335603 | 1.133483 | 0.090347 | -0.177305 |
| ENSDARG00000045904 | *nr2e3* | nuclear receptor subfamily 2, group E, member 3 | 1.550197 | 1.437405 | 0.251539 | 1.337303 |
| ENSDARG00000069998 | *si:ch211-145b13.6* | si:ch211-145b13.6 | 1.344335 | 1.578676 | 0.776562 | 1.049328 |
| ENSDARG00000006868 | *trh* | thyrotropin-releasing hormone | 1.196491 | 1.042495 | 0.394614 | -0.057611 |
| ENSDARG00000024325 | *cert1a* | ceramide transporter 1a | 1.187151 | 1.434403 | 0.862009 | 0.767827 |
| ENSDARG00000071562 | *mtus1a* | microtubule associated tumor suppressor 1a | 1.374691 | 1.32935 | 0.759931 | 0.949522 |
| ENSDARG00000051914 | *slc14a2* | solute carrier family 14 member 2 | 1.824428 | 2.01495 | 0.824428 | 0.841302 |
| ENSDARG00000098293 | *si:dkey-27i16.2* | si:dkey-27i16.2 | 1.038668 | 1.117172 | 0.434501 | 0.749161 |
| ENSDARG00000100560 | *zfpm2b* | zinc finger protein, FOG family member 2b | 1.400461 | 1.113924 | 0.641029 | 0.092794 |
| ENSDARG00000063670 | *gtf2a1l* | general transcription factor IIA, 1-like | 1.397839 | 1.069497 | 0.832298 | 1.437117 |
| ENSDARG00000101952 | *pigv* | phosphatidylinositol glycan anchor biosynthesis, class V | 1.117695 | 1.278976 | 0.278976 | 1.34861 |
| ENSDARG00000077382 | *hcn5* | hyperpolarization activated cyclic nucleotide-gated potassium channel 5 | 2.166192 | 1.687477 | 1.412939 | -0.086415 |
| ENSDARG00000010630 | *hoxb6a* | homeobox B6a | 1.108607 | 1.293207 | 0.472641 | -0.265691 |
| ENSDARG00000095930 | *myha* | myosin, heavy chain a | 5.43105 | 3.478047 | 3.099536 | -1.807355 |
| ENSDARG00000031658 | *si:ch211-207d6.2* | si:ch211-207d6.2 | 1.419903 | 2.080565 | 0.504792 | 0.130397 |
| ENSDARG00000034896 | *ldb2b* | LIM domain binding 2b | 1.695546 | 1.186963 | 0.053439 | 0.218499 |
| ENSDARG00000000212 | *krt97* | keratin 97 | 2.25164 | 3.871485 | 1.454839 | 0.331982 |
| ENSDARG00000093233 | *si:ch211-215d8.2* | si:ch211-215d8.2 | 1.927396 | 1.865591 | 1.056413 | 1.073337 |
| ENSDARG00000004643 | *cdhr1a* | cadherin-related family member 1a | 1.463656 | 1.31259 | 0.472068 | 0.753163 |
